# Standardizing a human TBMNK cell assay across instrument platforms: a new framework for producing high-quality, AI-ready reference dataset

**DOI:** 10.3389/fimmu.2026.1946801

**Published:** 2026-09-28

**Authors:** Yu-Fen Wang, Huan-Yu Chen, Bor-Sheng Ko, Ningchun Xu, Nathan Dwarshuis, James Wood, Paul DeRose, Santosh Putta, Yu Qian, John Quinn, John T. Elliott, Virginia Litwin, Robert Hoffman, Sheng Lin-Gibson, Lili Wang, The Flow Cytometry Standards Consortium Working Group 2

**Affiliations:** 1 AHEAD Medicine Corporation, San Jose, CA, United States; 2 Department of Internal Medicine, National Taiwan University Hospital, Taipei, Taiwan; 3 Biosystems and Biomaterials Division, Material Measurement Laboratory, National Institute of Standards and Technology (NIST), Gaithersburg, MD, United States; 4 Cytometry and Biophotonics Consultant, Galax, VA, United States; 5 BioLegend/Revvity, San Mateo, CA, United States; 6 J. Craig Venter Institute, San Diego, CA, United States; 7 BD Biosciences, San Jose, CA, United States; 8 International Society for Advancement of Cytometry (ISAC), Bethesda, MD, United States; 9 Independent Consultant, Livermore, CA, United States

**Keywords:** antigen expression, calibration and standardization, cell subset percentage and count, cytometer detection sensitivity, equivalent reference fluorophores (ERF), manual and automated data analysis, standard operating procedure (SOP), TBMNK cell assay

## Abstract

**Introduction:**

Recent advances in regenerative medicine advanced therapies (RMAT) are revolutionizing health care by providing curative treatments for previously untreatable diseases. Flow cytometry assays have been used to measure critical quality attributes, including viability, identity, purity, strength, and potency of the RMAT. However, the lack of reproducibility, comparability, and measurement confidence of results across various flow cytometry platforms, sites, and over time remain a major challenge.

**Method:**

Through a large-scale interlaboratory study (ILS), coordinated by the NIST Flow Cytometry Standards Consortium, a public-private partnership, we carried out study to identify T/B/Monocyte/Natural Killer (TBMNK) cells and to measure cell viability and health status. This ILS used common study samples, associated instrument control- and assay-reagents, and four standard operating procedures (SOPs) to enable end-to-end assay standardization across 42 cytometers from 7 manufacturers across 21 sites.

**Results:**

We present the first demonstration for achieving standardized cytometric assays across a wide range of instrument platforms. The results of the ILS were reported in quantitative units with metrological traceability to the SI (International System of Units): number of cells per µL for cell count and Equivalent Reference Fluorophore (ERF) units for expression levels of CD markers. The standardization approach puts flow cytometric data from different instrument platforms on the standardized ERF scale.

**Discussion:**

To overcome the challenge of instrument-driven variability, this ILS utilizes a human TBMNK cell assay to establish and validate a new standardization framework. By reporting results in SI-traceable units, this work demonstrates that comparable measurements of viable, non-apoptotic TBMNK subsets are achievable across different cytometers. The resulting high-quality, artificial intelligence (AI)-ready reference dataset provides a critical foundation for the validation of AI/Machine Learning (ML) technologies in translational immunology. Additionally, this study underscores that the integration of automated sample preparation is a necessary next step to further minimize assay uncertainty and enhance cross-platform reproducibility.

## 1 Introduction

Flow cytometry is one of the most powerful, high-throughput single cell analysis techniques and plays a critical role in immunological research, vaccines and therapeutics discovery, development and approval of drugs and devices, disease diagnosis, and therapeutic treatment and monitoring. Over the past few years, the technology has evolved from a gold standard method for CD4^+^ lymphocyte counting for monitoring of HIV/AIDS (1) and for CD34^+^ cell counting for hematopoietic stem cell transplant (2), a critical tool for diagnosing and classifying various types of blood cancers (3, 4) and hematological and autoimmune diseases (5), to an essential technique for characterizing advanced live cell-based drugs (6) with the ability to measure up to 50 markers at the single cell level (7).

The 2024 US Food and Drug Administration (FDA) Guidance Document on Considerations for the Development of Chimeric Antigen Receptor (CAR) T Cell Products (8) recognizes flow cytometry as a critical analytical tool for cell viability, identity, purity, and potency. FDA recommends that the initial investigational new drug (IND) submission includes detailed information on flow cytometric assays, such as description of assay, instrument calibration and quality control (QC), assay controls, assay standard operating procedure (SOP), antibody titration, direct detection of CAR, and comprehensive assay validation for lot release to support licensure. These recommendations have drastically increased the need for high quality, robust, and validated flow cytometric assays.

Despite many efforts toward flow cytometry instrument calibration and standardization as well as assay harmonization (9, 10), challenges remain due to the complexity of the technology and the lack of reference standards, and particularly with respect to measurement confidence and comparability from different instruments, sites and over time. These challenges hinder consistent critical decision-making in research, manufacturing, and clinical applications. Furthermore, high-quality, well-annotated flow cytometric datasets are sparse in the public domain which limits the development of artificial intelligence and machine learning (AI/ML) approaches. It also impedes integration of flow cytometry datasets with omics datasets to support system biology approaches for precision medicine.

To address these unmet challenges, NIST launched the Flow Cytometry Standards Consortium (FCSC) (11) in 2020 to work with key stakeholders to jointly develop standards and measurement solutions to support the broad adoption of quantitative flow cytometry technologies. At present, five working groups (WGs) are working to advance 1) instrument calibration and standardization, 2) cytometric assay standardization, 3) centralized data analysis, 4) measurement assurance issue on various gene delivery systems, and 5) AI/ML approaches to flow cytometry applications.

This manuscript presents the findings of the first interlaboratory study (ILS) led by Working Group 2 (WG2) to standardize cell count and health assays, utilizing a flow cytometry-based T/B/Monocyte/Natural Killer (TBMNK) as the primary model (**Figure 1**). Four objectives were set for the ILS: 1) to design the assay with fixed variables including test samples, assay panel, and instrument control materials, 2) to develop common study SOPs that will enable result comparability in standardized units across instrument platforms, 3) to generate data for evaluating suitability of test materials as potential future reference standards, and 4) to produce high-quality dataset for data analysis studies to identify reference data standard. Here, we present a comparative analysis of the data produced and subsequently analyzed by participants following provided SOPs (Supplemental documents). The site-reported manual analysis results on percentages of cell subsets are compared with those from centralized automated data analysis (CDA) procedures conducted by WG3 (Working Group 3) that is ongoing to demonstrate approaches to standardizing cell population identification from cross-platform flow cytometry data. Expected ILS outputs include assay standardization approach, SOPs, and reference dataset.

**Figure 1:**
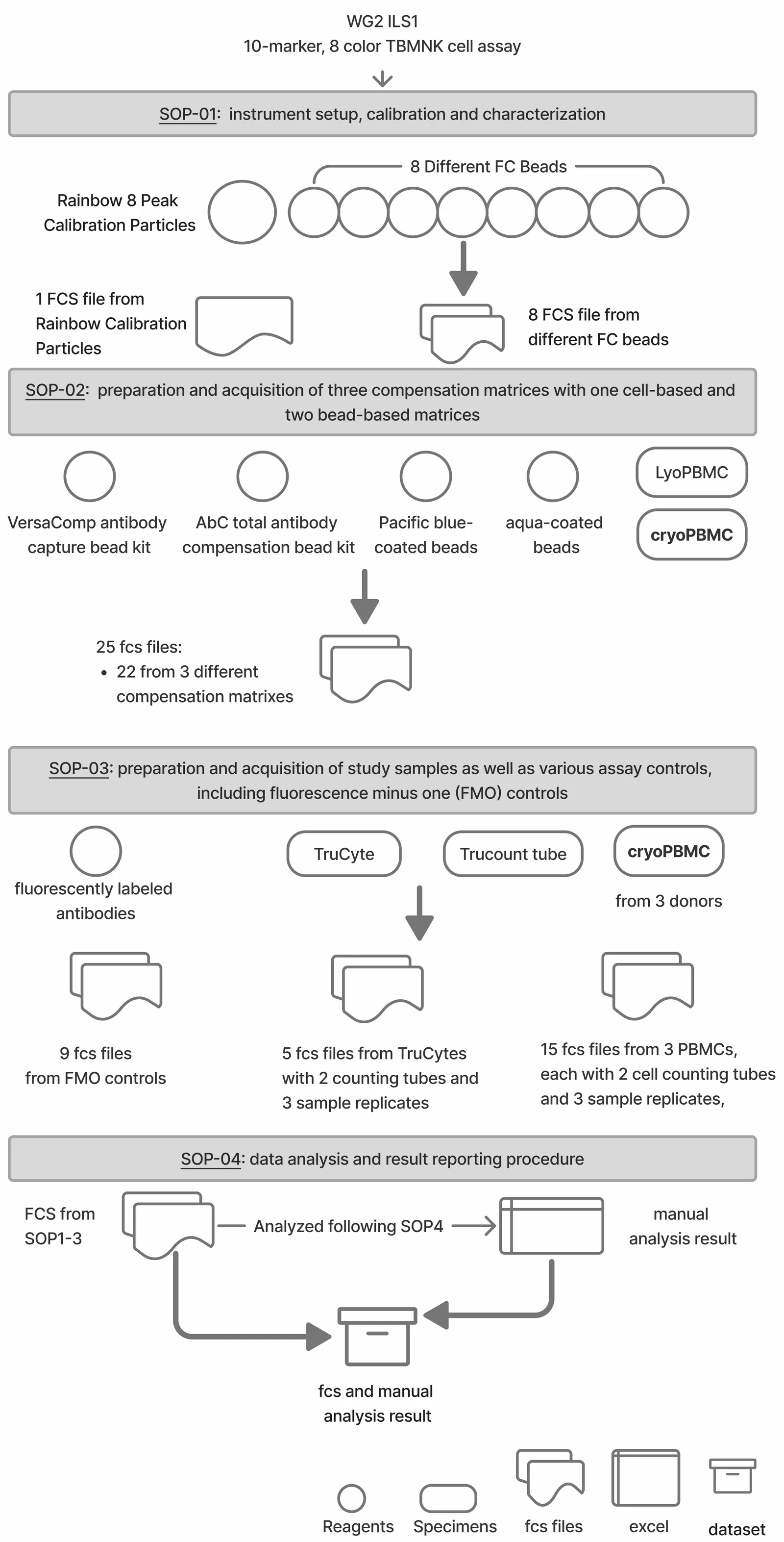
Standardized operating protocols (SOPs) for the TBMNK inter-laboratory study (ILS). Working Group 2 (WG2) developed four SOPs to ensure cross-site harmonization and standardization of TBMNK cell count and health analysis: a) SOP-01: Instrument Setup, Characterization and Calibration using Rainbow 8-peak calibration and 8 different FC beads. b) SOP-02: Preparation and acquisition of 3 compensation matrices using cryoPBMC, lyoPBMC, VersaComp, AbC Total, Pacific Blue, and Aqua beads. c) SOP-3: Preparation and acquisition of study samples, including 3 donors’ cryoPBMC samples and TryCytes, integrated with absolute count controls (TruCount) and FMO controls. d) SOP-04: Data analysis pipeline comparing automated FCS file processing with manual analysis results exported to standardized reporting formats. This framework minimizes inter-instrument variability and standardizes marker expression levels in SI-traceable, instrument independent units: cell count in number of cells per µL and expression level of a CD marker in ERF unit, ensuring comparable, high-quality dataset across all participating laboratories. cryoPBMC, cryopreserved peripheral blood mononuclear cell; FC, fluorescent compensation; ILS, inter-laboratory study; lyoPBMC, lyophilized peripheral blood mononuclear cell; SOP, standard operation protocol; WG, working group.

## 2 Methods

### 2.1 Study designs

This study used a 10-marker, 8-color TBMNK cell assay including viability and apoptosis markers. As shown in **Figure 1**, four SOPs guided the sequential experiments:

1. WG2 SOP-01: Fixed assay detectors and detailed instrument calibration and characterization.
2. WG2 SOP-02: It details the preparation and acquisition of three compensation matrices, comprising one cell-based and two bead-based matrices.
3. WG2 SOP-03: Defined protocols for sample preparation, FMO (Fluorescence Minus One) controls, and WG3-standardized file naming.
4. WG2 SOP-04: Established gating strategies for phenotyping donors’ peripheral blood mononuclear cells (PBMCs) and TruCytes™.

Cytometer sensitivity (Q) and CD marker expression were calculated in Equivalent Reference Fluorophore (ERF) units using a modified automated spreadsheet (12–14). Results were recorded in the study reporting spreadsheet in accordance with SOP-04.

### 2.2 Materials and instruments

The materials and reagents utilized in the study are listed in **Table 1**. In SOP-01, Rainbow 8 Peak Calibration Particles (Spherotech, Lake Forest, IL, United States) were used, along with 8 different Fluorescent Control (FC) beads (BD Biosciences, San Jose, CA, United States) as the reagents. In SOP-02, cryo-preserved PBMCs (cryoPBMC) from one of three healthy donors procured from AllCells (Alameda, CA, United States), and lyophilized PBMCs (lyoPBMC) from National Institute for Biological Standards and Control (NIBSC, Potters Bar, United Kingdom) were used as the tested cells for cell-based compensation. Two compensation bead kits (VersaComp antibody capture bead kit (Beckman Coulter, Miami, FL, United States) and AbC total antibody compensation bead kit (Thermo Fisher, Eugene, OR, United States) as well as Pacific blue-coated and aqua-coated beads (Thermo Fisher) were used for the purpose of bead-based compensation. For SOP-03, TruCytes synthetic cells from Slingshot Biosciences (Emeryville, CA, United States) served as a QC sample, and 3 healthy donors’ cryopreserved PBMCs were sent directly from AllCells to participants in a liquid nitrogen tank that included a temperature tracker as the study samples to build a solid foundation for future assay studies. Fluorescently labeled antibodies from BioLegend (San Diego, CA, United States) and TruCount Counting Tubes from BD Biosciences were used for assay testing.

**Table 1:** Materials and reagents used in the ILS along with their abbreviated names and suppliers.

SOP	Reagents/materials	Abbreviation	Supplier
SOP-01	Rainbow 8 Peak Calibration Particles	RCP-30-5A	Spherotech
SOP-01	FC FITC	FC-FITC	BD Biosciences
SOP-01	FC PerCP-Cy5.5	FC-PCP-C5	BD Biosciences
SOP-01	FC PE	FC-PE	BD Biosciences
SOP-01	FC PE-Cy7	FC-PC7	BD Biosciences
SOP-01	FC APC	FC-APC	BD Biosciences
SOP-01	FC APC-Cy7	FC-APC7	BD Biosciences
SOP-01	FC V450	FC-V450	BD Biosciences
SOP-01	FC V500-C	FC-V500C	BD Biosciences
SOP-02	CryoPBMC	cryoPBMC	AllCells
SOP-02	Lyophilized PBMC	lyoPBMC	National Institute for Biological Standards and Control (NIBSC)
SOP-02	VersaComp Antibody Capture Bead Kit	Versacmp	Beckman Coulter Life Sciences
SOP-02	AbC Total Antibody Compensation Bead Kit	AbCTotal	Thermo Fisher Scientific
SOP-02	Aqua-coated Bead	AqBead	Thermo Fisher Scientific
SOP-02	Pacific Blue-coated Bead	PBBead	Thermo Fisher Scientific
SOP-03	CryoPBMC	cryoPBMC	AllCells
SOP-03	TruCytes TBMNK synthetic cells	TruCytes	Slingshot Biosciences
SOP-03	CD45 PerCP-Cy5.5	CD45	BioLegend
SOP-03	CD3 FITC	CD3	BioLegend
SOP-03	CD19 APC	CD19	BioLegend
SOP-03	CD14 APC	CD14	BioLegend
SOP-03	CD56 PE	CD56	BioLegend
SOP-03	CD16 PE	CD16	BioLegend
SOP-03	CD4 PE-Cy7	CD4	BioLegend
SOP-03	CD8 APC-Cy7	CD8	BioLegend
SOP-03	Annexin Pacific Blue	AxPB	Thermo Fisher Scientific
SOP-03	Aqua	AqLD	Thermo Fisher Scientific
SOP-03	TruCount Counting Beads	TruCount	BD Biosciences

ILS, Inter-laboratory study; SOP, Standard operating protocol; NIBSC, National Institute for Biological Standards and Control.

The study was approved by the institutional research protection office of NIST (Project #MML-2019-0125) and involved seventeen consortium member organizations across 21 sites, utilizing a total of 42 different cytometers from 7 instrument companies. The types of cytometers used, including the instrument model, number under each instrument type, type of detector, and their manufacturers, are detailed in **Supplementary Table 1**. The diverse instrument platforms consist of conventional, spectral, and imaging cytometers and are equipped with different types of photodetectors, photomultiplier tube (PMT) or silicon photomultiplier (SiPM), avalanche photodiode (APD), and charge-coupled device (CCD), respectively. Among all instrument platforms, Amnis cytometers are equipped with high power lasers (> 100 mW) compared to most flow cytometers with laser power in the range of 30 mW to 50 mW.

### 2.3 Assignment of ERF and Absolute Reference Intensity values to FC beads

The ERF and ARI value assignment was performed according to published SOPs (15, 16). The ERF values assigned are excitation wavelength dependent and traceable to 8 NIST reference fluorophore solutions with defined uncertainties. This process establishes an absolute, emission filter-independent fluorescence scale, determined by reference fluorophore solutions with known concentrations, which is proportional to the fluorescence intensity measured by the cytometer’s detector. The second fluorescence intensity values referred to as ARI assigned to microbead suspensions are emission filter dependent (15). Importantly, ERF values are assigned under standard filters, defined by NIST, that have large filter bandwidths commonly used in different flow cytometers. This instrument independent ERF concentration scale can be used to assign values under an appropriate laser excitation to different calibration microbead sets for diverse cytometers that use different emission filters. ARI values are most useful for characterizing fluorescence characteristics for a particular flow cytometer.

### 2.4 The TBMNK assay

The procedures in SOP-03 were structured to address two main sample types: thawed PBMC samples (3 separate vials from 3 different normal donors) and reconstituted synthetic TruCytes QC samples (3 separate vials of the same production lot). A thawing protocol for cryoPBMC samples is also included in the SOP-03. Once cryoPBMC samples were thawed, cells were first stained with Aqua (live/dead stain), followed by two washes to remove excess dye. Upon reconstitution of Aqua-stained PBMCs, a specific volume was added to a TruCount tube. Anti-CD45 PerCP-Cy5.5 and Annexin V Pacific Blue were then added to the counting tube. The sample was then incubated for 30 min at room temperature, followed by the addition of 0.5 mL of a buffer solution. For PBMC immunophenotyping tubes and FMO controls, labeled antibodies were added to specified volumes of Aqua-stained PBMCs based on the SOP-03. After staining and two washes, stained samples were then acquired using a flow cytometer. All antibodies were titrated to ensure sample staining under saturated conditions.

Based on the data analysis and gating strategy provided in the SOP-04, percentages of different cell subsets and event counts as well as median fluorescence intensity (MdFI) and associated uncertainties of all CD markers were determined. The concentration of the viable, non-apoptotic CD45^+^ mononuclear cells was obtained from the PBMC counting tube. This concentration was applied to the TBMNK assay tube results to calculate the concentrations of different cell subsets relative to the viable, non-apoptotic CD45^+^ mononuclear cells. In the study, participants were only required to reconstitute TruCytes based on a procedure recommended by the manufacturer. For the cell counting tube and TBMNK assay tube of TruCytes, the staining procedures were essentially the same as thawed PBMCs except for no Aqua staining and washing step and no Annexin V Pacific Blue in the TBMNK antibody panel.

### 2.5 Data acquisition and manual analysis

Based on the three experimental SOP-01 to SOP-03, participants were expected to produce a total of 60 data files in the flow cytometry standard (FCS) format per instrument. These FCS files are composed of 1 from Rainbow 8 peak beads, 8 from different FC beads, 22 from 3 different compensation matrixes, 9 from FMO controls, and 15 from 3 healthy individual’s PBMCs, each with 2 cell counting tubes and 3 sample replicates, as well as 5 from a single TruCytes QC sample with 2 counting tubes and 3 sample replicates (**Figure 2**). Besides submitting all 60 raw FCS files for CDA, participants were asked to follow step instructions in SOP-04 and perform their own analyses to report percentage and count of defined cell populations, expression levels of CD markers, and detector sensitivity (Q).

**Figure 2:**
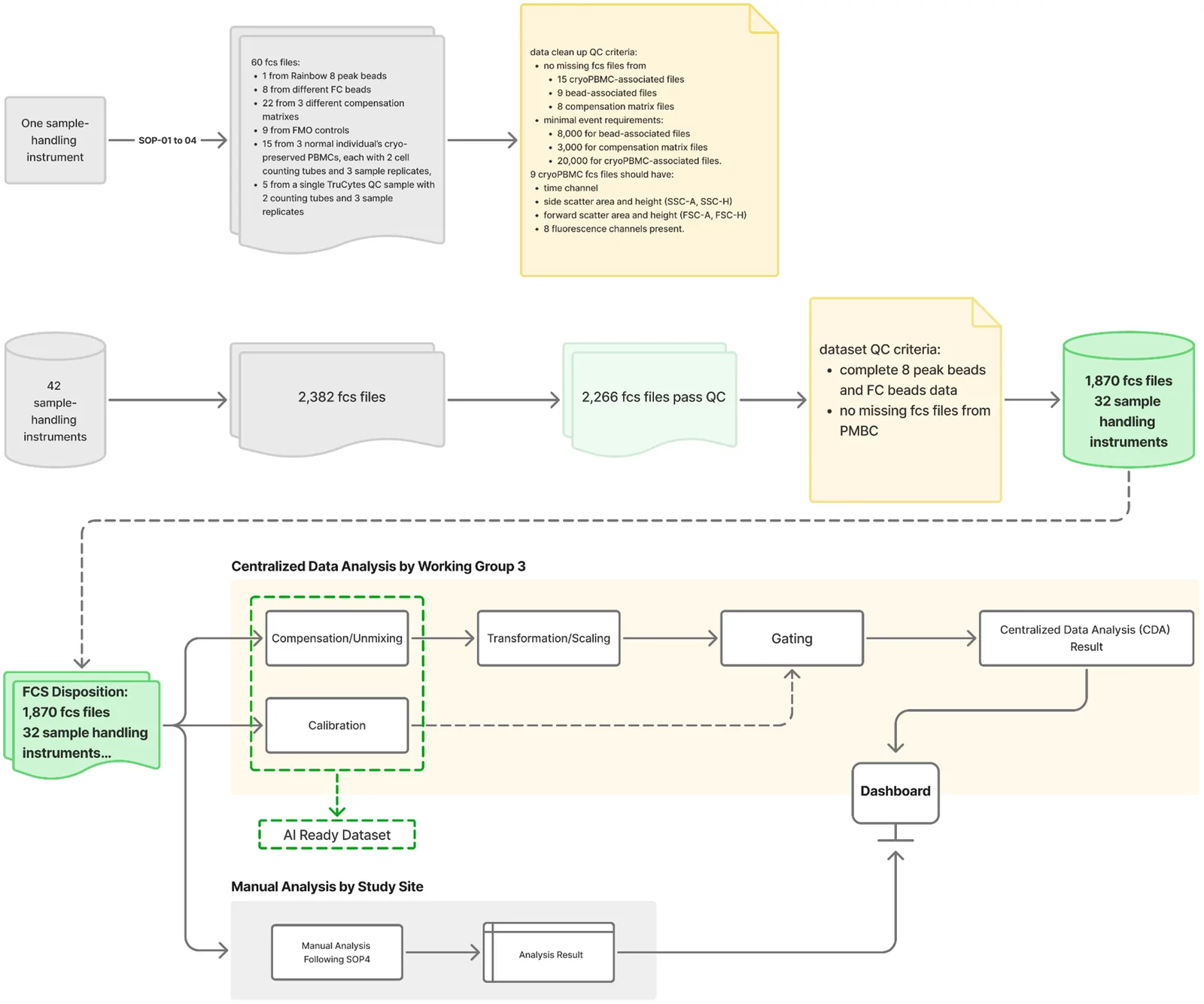
Standardized QC workflow for manual and centralized data analyses. A standardized data quality control workflow was applied to FCS files generated by each participating site in the WG2 ILS. The process consisted of (1): Verifying that the correct number of FCS files were produced according to the SOP and sample requirements; for a complete dataset from a single instrument, 60 FCS files are expected. (2) Assessing each FCS file for QC criteria, including a total event count greater than the established threshold for the given sample type and the absence of missing channel information. (3) Compiling a list of FCS files that passed QC for each instrument and determining the number of sample handling-instruments with a complete set of FCS files meeting all QC criteria. Following the file quality check, a total of 32 sample handling -instrument datasets (1,870 FCS files) were subsequently processed with detector/parameter naming standardization, compensation/unmixing, transformation/scaling, automated gating, and calibration. The calibrated or compensated/unmixed dataset is planned for a separate manuscript as an AI-ready dataset. The automated gating results from the CDA were compared against the manual analysis results provided by the study participants following SOP-04. The aggregated results are presented in an interactive visualization dashboard hosted online at a dedicated project website. FC, fluorescent compensation; QC, quality control; SOP, standard operation protocol.

### 2.6 File cleaning before analysis of the reported results

Owing to experimental issues, some participants submitted less than 60 raw FCS files. Additionally, issues were identified in the submitted raw data files, such as missing time channels and having insufficient events. Therefore, three QC criteria were used to select technically valid data files; reported results associated only with these clean-up data files by participants are analyzed and compared with CDA performed on the same selected data files. Since this is TBMNK assay study on healthy donor PBMCs (1), the dataset must be complete, specifically containing all PBMC-associated files (n=15), all bead files (n=9), and one complete compensation matrix (n=8). After passing the first criterion (2), the files must meet minimal event requirements, 8000 for bead-associated files, 3000 for compensation matrix files, and 20000 for PBMC-associated files (3). FCS files from PBMC samples should have a time channel, side scatter area and height, and forward scatter area and height as well as all 8 fluorescence channels present (**Figure 2**). Only FCS files passing all 3 selection criteria are analyzed by the CDA pipeline. The results from these files analyzed and reported by participants are presented in this report. Additionally, participants’ manual analysis results on cell subset percentages are compared with the results from the same instrument platforms by automated CDA.

### 2.7 Assay result visualization

The automated QC to check against the criteria specified above was conducted on submitted FCS files using two orthogonal methods, one with Python programming language (version 3.12) and another by a snakemake (version 7.32.4) pipeline with a combination of Python (version 3.12.6) and R (version 4.4.3) scripts. The results from the QC, which included information on the FCS files from each site instrument, the SOP used, and any failed criteria, were compiled using the gradio package. These results were then presented through an interactive visualization dashboard deployed to a dedicated project website at https://miganalytics.nist.gov/content/4269cf8d-b270-44ae-a4e4-b1f8d7d9ee96/. This dashboard is designed to enable consortium members and the broader community to explore the interlaboratory study results, perform comparative analyses, and leverage the high-quality dataset for future reference standard and AI/ML development efforts. For the expression analysis of 7 CD markers, the ERF values from sample triplicates per donor per instrument were first averaged; the mean ERF values were then used to compare the mean values across different instruments.

### 2.8 Statistical analysis

All analyses were descriptive and aimed at quantifying measurement variability across sites and instrument platforms. Analyses were carried out in Python (version 3.12) using numpy (version 1.26.4) package and statsmodels (version 0.14.6) package. Only results from the 32 cytometers that passed the QC were included. For antigen expression, ERF values from the sample triplicates acquired per donor per instrument were averaged before cross-instrument comparison, and dispersion is reported as the coefficient of variation (CV), calculated as the standard deviation divided by the mean.

Outliers were defined as instrument values deviating by more than 1.96 standard deviations from the mean (|z| > 1.96), calculated per marker per donor across the 32 cytometers. Outliers were reported rather than removed: their effect on the CV of the ERF values is stated explicitly in the Results, and no values were excluded from the reported summaries on statistical grounds. Variance in CD3^+^ T-cell population measurements, from site-reported manual analysis was apportioned among four sources — combined sample handling, instrument and manual analysis; compensation control; sample replicate; and residual — by analysis of variance (ANOVA). The CV of the CD3^+^ lymphocyte percentage from site-reported manual analysis was compared descriptively with that obtained by centralized data analysis (CDA) on the same QC-passed files.

## 3 Results

### 3.1 The QC inspection of data files

A total of 2382 FCS data files, collected from 42 instruments, was inspected based on QC criteria detailed in the ‘Methods,’ of which 2266 files passed. However, only 32 out of 42 instruments passed the overall quality check, that required completed 8-Peak Calibration Particles, FC beads data, and no missing FCS files from PBMC samples. Hence, only results reported by these 32 cytometers were analyzed. The number of passing cytometers is indicated in the third column of **Supplementary Table 1**. The QC process workflow is shown in **Figure 2**, and the visualization of the QC inspection results for all 42 flow cytometers is provided in **Figure 3** (where instruments with missing files are represented by orange dots and those with file errors by colored hexagons). The complete inspection results are accessible on the interactive online dashboard given in Section 2.7 of the ‘Methods’.

**Figure 3:**
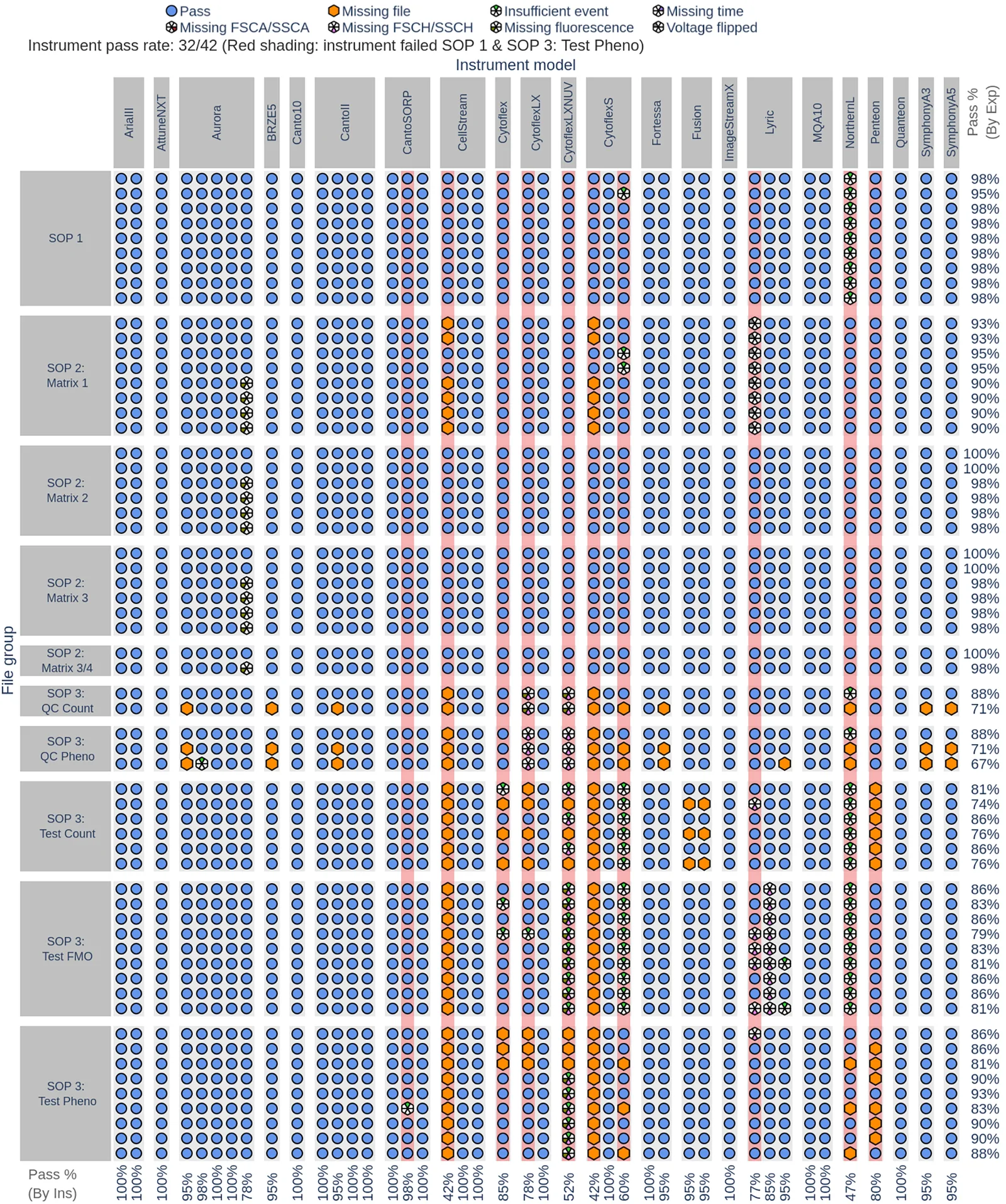
Dashboard visualization of QC results across samples and instruments following different SOPs. The dataset is visualized as an array of symbols, with 42 columns representing sample handling instruments and 60 rows representing SOP-specified experiments. Each symbol corresponds to the QC status of an individual FCS file. Blue circles indicate files that passed quality control (QC), while orange hexagons denote missing FCS files. Multicolored hexagons indicate specific QC failure modes, including missing scatter or fluorescence channels (FSC-A/SSC-A, FSC-H/SSC-H, or fluorescence), missing time channel, insufficient events, and voltage inversion. An instrument is considered to have passed QC if all experiments within both the SOP-1 block and the SOP-3: Test Pheno block passed QC. Based on this criterion, 32 out of 42 site instruments passed QC and were included in downstream analyses. Red shaded column backgrounds indicate instruments that failed this SOP-based QC criterion (i.e., at least one required experiment within SOP-1 or SOP-3: Test Pheno did not pass QC).

### 3.2 Percentage of cell subsets and count of cell subsets

Study SOP-04 (Supplemental document), which provides stepwise gating strategy and formula for participants to compute the concentration of viable, non-apoptotic CD45^+^ mononuclear cells. Participants were encouraged to determine cell concentration volumetrically when appropriate.

**Figures 4A, B** shows the percentages and absolute cell counts of different cell subsets from a donor’s PBMC and TruCytes across different cytometers, respectively. Note that only participants submitted their results from manual analysis (Columns 4 and 5 of **Supplementary Table 1**) and cell counts calculated using TruCount beads as an internal standard are shown. Overall, the results show cell percentages and counts are consistent but vary by sample-handling instruments. Additionally, TruCytes samples display greater consistency than PBMCs in major cell subset populations except for NK cells across instruments. **Figure 4C** shows CD3^+^ T cell percentages obtained from 3 healthy donors’ PBMCs under 3 compensation matrices across instruments. As described in Section 2.2 of the ‘Methods’ and shown in **Table 1**, compensation matrix 1 is cell based using a donor’s cryoPBMC and lyoPBMC whereas matrices 2 and 3 are bead-based using VersaComp antibody capture bead kit and AbC total antibody compensation bead kit, respectively. Clearly, this study demonstrates that both cell- and bead-based matrices work equally well.

**Figure 4:**
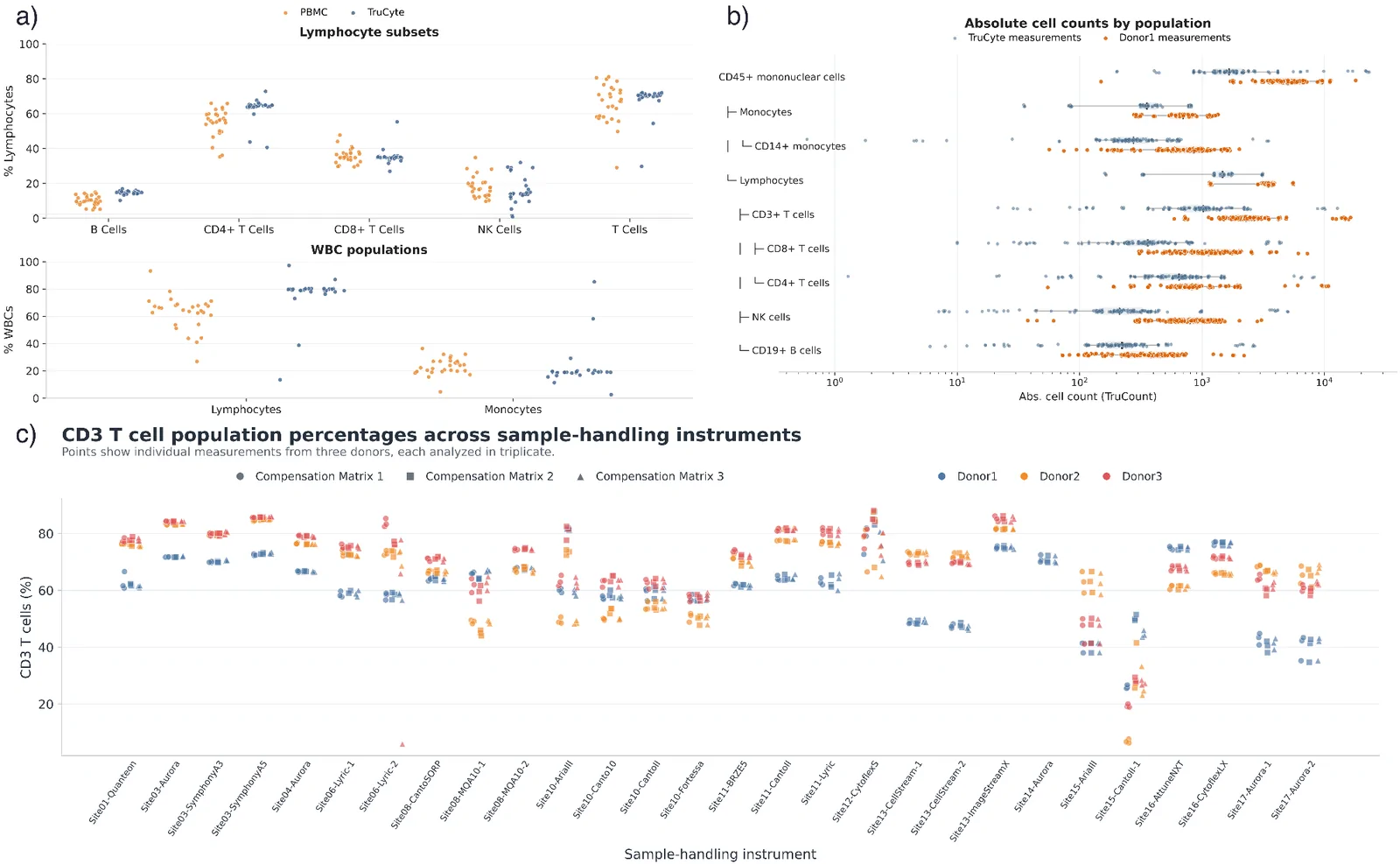
Comparison of cell subset population frequencies and absolute cell counts in PBMC and TruCytes samples. **(A)** Percentages of lymphocyte subsets (B cells, CD4^+^ T cells, CD8^+^ T cells, NK cells, and total T cells; top) and major white blood cell populations (lymphocytes and monocytes; bottom) measured in PBMC samples (orange) and TruCytes samples (blue). Each point represents results from an individual instrument. **(B)** Absolute counts of CD45^+^ mononuclear cells and their subsets measured using TruCount beads as the internal counting standard. Results from PBMC donor 1 (orange) and TruCytes samples (blue) are shown across instruments. **(C)** CD3^+^ T-cell percentages measured in PBMC samples from 3 healthy donors under 3 compensation matrices across instruments. Compensation matrix 1 is cell-based whereas matrices 2 and 3 are bead-based using two bead kits from two different vendors. Colors indicate donors and symbols indicate the 3 independent compensation matrices. Points represent triplicate measurements.

### 3.3 Analysis of CD marker expression

Central to this study is the standardization of CD marker expression analysis across different cytometers, which is accomplished through the ERF calibration approach. Linearities of fluorescence detectors are established using Rainbow 8 Peak Calibration Particles. Subsequently, the use of fluorophore-specific FC beads assigned with ERF values by NIST enables the scale transformation of detector channels to an instrument independent ERF scale. NIST assigned ERF values for 8 different FC beads are provided in **Supplementary Table 2**. Based on the established ERF scales, **Figure 5** shows the resulting expression values of 7 CD markers averaged in ERF units over the 32 flow cytometers.

**Figure 5:**
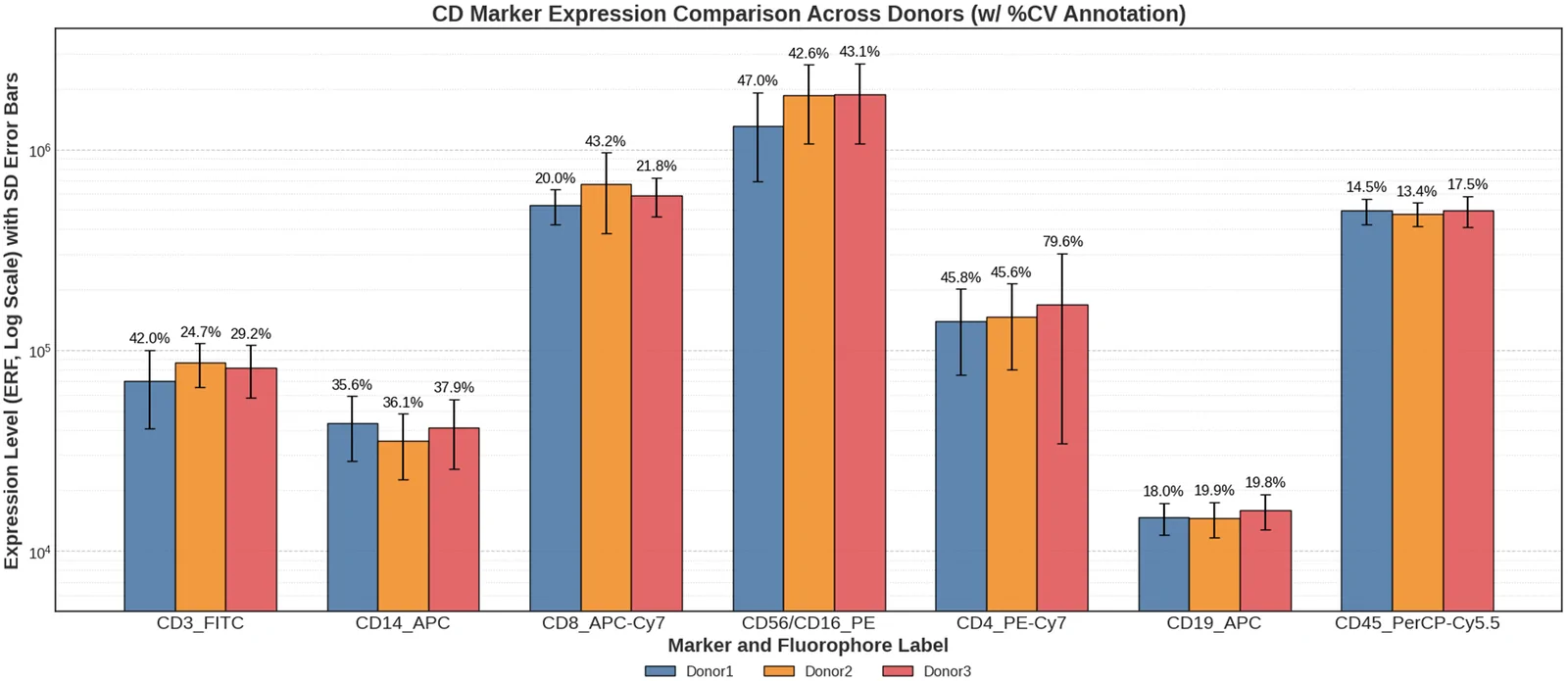
Mean expression level of 7 CD markers in equivalent number of reference fluorophores (ERF) units across 32 cytometers. The figure presents the mean expression values of 7 CD markers: CD3 FITC, CD14 APC, CD8 APC-Cy7, CD15/CD56 PE, CD4 PE-Cy7, CD45 PerCPCy5.5, CD19 APC, reported in ERF units and averaged over 32 flow cytometers. For each CD marker, ERF values from sample triplicates per donor per instrument were initially averaged. The final mean ERF values and % CV were then calculated across the participating instrument platforms.

The expression levels of 7 markers per donor, except for CD4 PE-Cy7 on donor #3, are with Coefficient of Variations (CVs) of < 50% and values for CD19 APC and CD45 PerCP-Cy5.5 are with CVs below 20% across 32 cytometers. Here, the expression levels of CD3-FITC, CD14-APC, and CD19-APC are determined in the units of Molecules of Equivalent Soluble Fluorophore (MESF), a special case of ERF where the reference fluorophore shown in the **Supplementary Table 2** and fluorophore on microbeads and cells are identical. The reference fluorophore for CD45 PerCP-Cy5.5, PE, and PE-Cy7 is Nile Red whereas the reference fluorophore for CD8 APC-Cy7 is Alexa Fluor 700 (AF700) (17). The consistent expression results of CD19 APC and CD45 PerCP-Cy5.5 suggest the ERF approach works as well as MESF methodology.

A few outliers (N ≤ 3) per donor PBMC, identified by z-score, inflated the CVs of the ERF values to be > 30% for CD3 FITC, CD14 APC, and CD8 APC-Cy7. The applicability of the ERF-based approach for antigen expression analysis across different cytometers is manifest in the CD19 APC expression on donor #2 shown here as an example (**Figure 6**). The MdFI values of CD19 APC on donor #2 differ by more than 42-fold with a CV of 122%; however, the ERF values of the same donor are consistent with a CV of < 19.9% across instruments. Similar expression results in MdFI and ERF units including CD19 APC on donor #2 shown in **Figure 6** appear across all CD markers and donors. These results show that ERF unit is a more optimal approach for standardizing flow cytometer platforms considering cytometer manufacturers currently use MdFI-based method to standardize their own instrument platforms.

**Figure 6:**
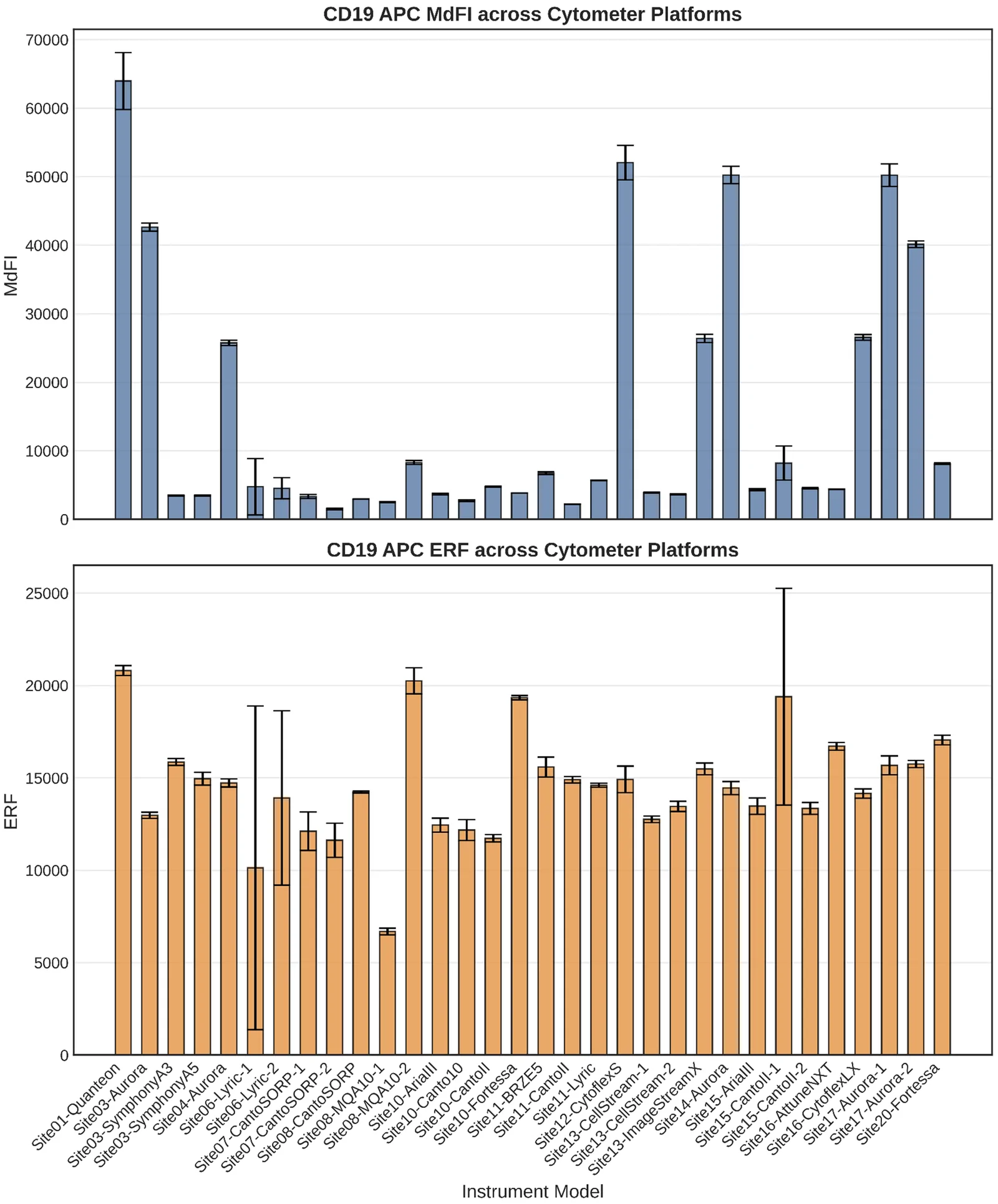
Platform-dependent variability of CD19 APC signals before and after ERF standardization. Expression level of CD19 APC on donor #2 measured across different flow cytometer platforms that passed quality control (QC) and outlier inspection. Data are presented as median fluorescence intensity (MdFI; top panel) and as ERF units (bottom panel). Each bar represents the median signal obtained from an individual instrument model, illustrating platform-dependent variability in raw fluorescence intensity and the improved comparability after ERF standardization.

### 3.4 Detector sensitivity

Study participants were instructed to compute Q values of the cytometer detectors using the provided spreadsheet built for this task following instructions provided by SOP-04. This automated calculation spreadsheet was modified from the original quantiFlash™ approach (12–14). Specifically, the spreadsheet computed Q values of the 8 fluorescence detectors after participants entered values of MdFI, rCV(robust coefficient of variation), and rSD (robust standard deviation) of the Rainbow 8-peak beads for the 8 fluorescence channels used, values of MdFI and rSD of the respective 8 FC beads, as well as ARI values of the 8 FC beads for specific flow cytometer (**Supplementary Tables 3**, **4** as examples). The Q values in a comparable and standardized unit of the statistical photon electron per ARI had been determined for the 32 cytometers that passed the initial QC inspection and are still under investigation. Values thought to be reliable from two most represented cytometer platforms, FACSCanto and Aurora are reported here and shown a consistent trend in **Supplementary Figure 1**. The Aurora cytometers equipped with avalanche photodiode (APD) detectors have higher Q values than the FACSCanto cytometers with PMT detectors across all 8 fluorescence detectors.

### 3.5 Comparison of results from manual analysis and CDA

The comparison of CD3^+^ lymphocyte frequency, derived from both site-reported manual analysis and CDA, was performed to evaluate variation introduced by instrument, specimen, and analysis process. As shown in **Figure 7B**, the CV of CD3^+^ lymphocyte percentage on 3 donors’ PBMCs reduced to ~15% by the CDA compared to >30% of CV by site-reported manual analysis.

**Figure 7:**
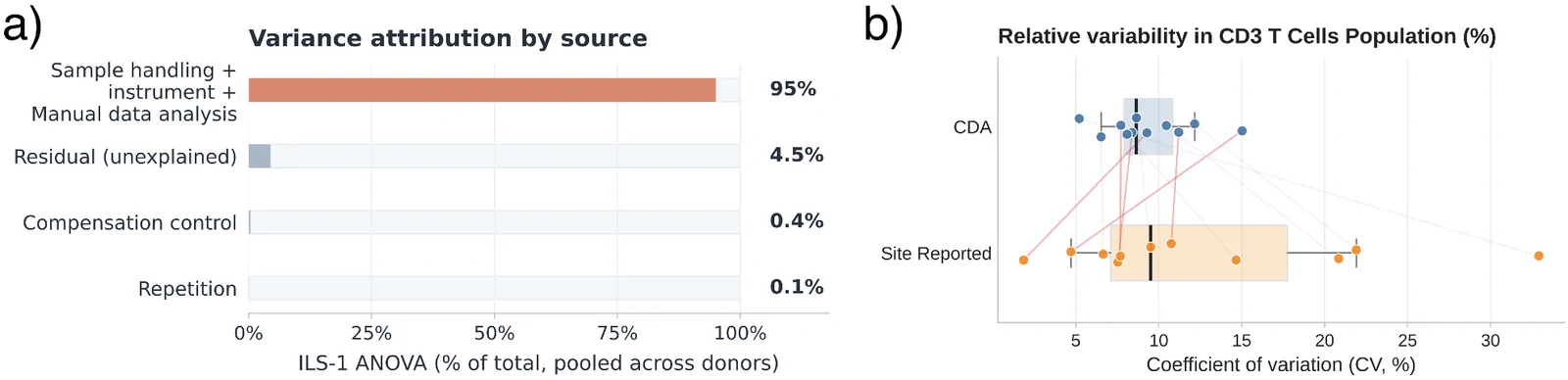
Sources of variability in CD3^+^ T-cell population measurements. **(A)** Variance contributions to CD3^+^ T-cell percentages determined by ANOVA and pooled across 3 donor PBMC samples. Variance was partitioned into sample handling–instrument–manual analysis, compensation control, measurement repetition, and residual components. **(B)** Coefficients of variation (CVs) of CD3^+^ T-cell percentages obtained from site-reported manual analysis and centralized data analysis (CDA). Each point represents a donor–compensation matrix combination, and lines connect paired measurements.

## 4 Discussion

This large-scale interlaboratory study examined 42 cytometers from 7 manufacturers to demonstrate the feasibility to standardize sample preparation, data acquisition and analysis with robust cross-platform consistency. Our methodology, distinct from approaches detailed in previous literature (10, 18), provides a scalable and reliable path for cross-platform data harmonization by leveraging existing calibration standards through the NIST ERF measurement service (15, 16). Cytometer calibration was performed using the same lot of 8-peak Rainbow beads and 8 FC beads to benchmark consistency across these diverse instruments. Standardization of the human TBMNK cell count and health assay was achieved using fixed study samples consisting of 3 healthy donors’ PBMCs, control samples, a TBMNK assay panel, and four SOPs. Assay results were reported in quantitative and traceable units, number of cells per µL for cell count, ERF unit for the expression level of a CD marker, and Spe/ARI (statistical photoelectron/ARI) for the detector sensitivity.

To resolve ERF value uncertainties caused by varying emission filters, an amended SOP (15) was implemented. Specifically, the spectrum of a reference fluorophore solution is integrated over a bandwidth of the main peak area down to ~10% of the maximum intensity, serving as a standard light bulb to quantify the fluorescence intensity of a calibration bead suspension. The known concentration of the reference fluorophore solution is used to quantify the fluorescence intensity in fluorophore concentration units to the bead suspension. When a standard filter is used, the intensity is expressed in instrument-independent ERF units, enabling standardization across different cytometers. Moreover, the exported FCS files of the study lack critical detector filter information and the necessary linkage between markers and detectors. This underscores the importance of adhering to the MIFlowCyt (Minimum Information about a Flow Cytometry Experiment) standard established by the International Society for Advancement of Cytometry (ISAC). MIFlowCyt requires comprehensive documentation of instrument identification and configuration, including fluidic, optical, and electronic subsystems, as well as detailed specifications of the optical path and flow cell (19).

This study demonstrates the applicability of the ERF-based approach for antigen expression analysis across diverse cytometer platforms. Although ERF values are not inherently linked to biological content such as Antibodies Bound per Cell (ABC) for antigen expression analysis commonly used in clinics, the ERF-based method mitigates certain limitations found in ABC-based methodologies, such as QuantiBrite PE calibration and the CD4 reference marker method (20). QuantiBrite PE calibration is limited to the PE fluorescence channel. The CD4 reference marker method, on the other hand, necessitates the use of reference PBMC samples and requires approximately identical quality of labeled antibodies for both the CD4 reference marker and the unknown antigen (21). Nonetheless, linking an ERF unit to an ABC unit using CD4 as the reference marker remains a feasible option that is under further investigation (21, 22).

Larger uncertainties of >40% CV are observed for the expression levels of CD4 PE-Cy7 and CD16/CD56 PE for all donors (**Figure 5**). One complexity is that both PE and PE-Cy7 fluorescence detectors can be under either 488 nm, 561 nm, or 532 nm laser excitation (Column 4 of **Supplementary Table 5**). The two CD markers, CD16 and CD56, and low NK cell number could contribute to the large variation observed. It is known that the CD4 expression level on human T helper cells is consistent between donors across literature reports (20, 22, 23). Here, the suitability of Nile Red as the reference fluorophore for PE-Cy7 (**Supplementary Table 2**) and different laser excitation wavelengths likely lead to the large uncertainty observed for ERF values of CD4 PE-Cy7. The PE-Cy7 emission spectrum peaks around 780 nm, while Nile Red shows minimal fluorescence at 780 nm. We are investigating the combinations of reagent choices and detection schemes to further improve result comparability in the next ILS. Additionally, three CD markers, CD19 APC, CD14 APC, and CD8 APC-Cy7 are under the red laser excitation. As shown in column 3 of **Supplementary Table 5**, the red laser wavelengths vary significantly from 628 nm to 642 nm across 32 cytometers. However, the variations of this laser wavelength and laser power do not contribute significantly to their expression results.

We present the first determination of cytometer detector sensitivities in the standardized, traceable unit of Spe/ARI, enabling cross-platform comparisons. This standardization is vital for high-sensitivity applications, including low-abundance biomarker immunophenotyping (24), functional assays (25), and rare event detection (5). Consistent with prior findings (26), we found that APD-based cytometers exhibit higher Q values than PMT-based instruments and that substantial sensitivity differences exist across detectors, even within the same model (**Supplementary Figure 1**). Note that Q values are only comparable between detectors sharing the same reference fluorophore (e.g., PE and PerCP-Cy5.5 via Nile Red), whereas using different references (e.g., APC vs. AF700) precludes direct comparison (**Supplementary Table 2**). Because TBMNK cells are sufficiently abundant in normal PBMCs, all 32 instruments met the sensitivity requirements for the study.

Manual gating analysis reported by individual sites show comparable results across replicates and different compensation matrices (**Figure 4**). ANOVA analysis was employed to evaluate technical variability from sample handing-instrument-manual analysis, compensation control, sample replicate, and residual (**Figure 7A**). Across all donors, the “sample handing-instrument-manual analysis” variable was the dominant source of uncertainty. The high consistency in percentages of major cell subset populations from TruCytes across instruments (**Figure 4A**) suggests that instrument was not the primary source of this uncertainty. Instead, cell percentages and counts from PBMC samples and cell counts from TruCytes (**Figures 4A, B**) infer that sample preparation across the 21 sites was the primary contributor. PBMC thawing, washing, and staining (SOP-02/03) are delicate procedures highly susceptible to operator-to-operator variability; techniques and timing are known to impact cell viability and non-specific antibody binding (27). The large variability in sample preparation highlights the challenge of data harmonization across sites and underscores the necessity for automated sample preparation. Moreover, we demonstrated that CDA was able to reduce overall variation (**Figure 7B**), showing that automating the data analysis workflow can further eliminate technical variability in the gating process and improve cross-sample comparison of cell populations (28, 29).

## 5 Conclusion

The lack of a standardized approach for achieving quantitative and traceable cytometric results across diverse platforms has historically hindered the development of reference datasets essential for AI/ML evaluation in immunology. This interlaboratory study successfully addresses this gap by utilizing a human TBMNK cell assay to validate a novel standardization framework. By reporting results in SI-traceable units—specifically cells per µL for absolute counts and ERF for CD marker expression—this work demonstrates that comparable measurements of viable, non-apoptotic TBMNK subsets are achievable across different cytometers. The resulting high-quality, AI-ready reference dataset provides a critical foundation for the validation of AI/ML technologies in translational immunology. Finally, this study underscores that the integration of automated sample preparation is a necessary next step to further minimize assay uncertainty and enhance cross-platform reproducibility.

## Group members of the flow cytometry-standards consortium working group 2

Elke Bergmann-Leitner, Walter Reed Army Institute of Research, 503 Robert grantAve, Silver Spring, MD 20910; Nicholas Battaglia, AstraZeneca, 1 MedImmune Way, Gaithersburg, MD 20878; Raffaello Cimbro, AstraZeneca, 1 MedImmune Way, Gaithersburg, MD 20878; Jeffrey Cobb, Beckman Coulter, Inc., 11800 SW147th Ave, Miami, FL 33196; Sandra Diebold, Medicine and Healthcare Products Regulatory Agency, National Institute for Biological Standards and Control, Blanche Lane, South Mimms, Potters Bar, Hertfordshire EN6 3QG,United Kingdom; Jennifer Fox, AstraZeneca, 1 MedImmune Way, Gaithersburg, MD 20878; Christele Gonneau, International Society for Advancement of Cytometry (ISAC),9650 Rockville Pike, Bethesda, MD 20814; Christina Grigoriadou, AstraZeneca, 1 MedImmune Way, Gaithersburg, MD 20878; Christopher Groves, IQVIA-TSAIL, 2400 Ellis Road, Durham, NC 27703; Garret Guenther, Agilent technologies, 11011 N. Torrey Pines Rd, Ste 150, LaJolla, CA 92037; Jerilyn Izac, Biosystems and Biomaterials Division, Material MeasurementLaboratory, National Institute of Standards and Technology(NIST), Gaithersburg, MD 20899; Jennifer Mantle, National Institute for Innovation in ManufacturingBiopharmaceuticals, 590 Avenue 1743, Newark, DE 19711; Mohamed Moustafa, AstraZeneca, 1 MedImmune Way, Gaithersburg, MD 20878; Anh-Tuan Nguyen, Slingshot Biosciences, 1250 45th Street, Ste 330, Emeryville,CA 94608; John Nolan, Cellarcus Biosciences, 6888 Nancy Ridge Dr, San Diego,CA 92121; Petra Pham, Bristol Myers Squibb, 400 Dexter Ave N, Ste 1200, Seattle,WA 98109; Haley Pugsley, Cytek Biosciences, 645 Elliott Ave W, Ste 100, Seattle,WA 98119; Deepa Rajagopal, Medicine and Healthcare Products Regulatory Agency,National Institute for Biological Standards and Control, BlancheLane, South Mimms, Potters Bar, Hertfordshire EN6 3QG,United Kingdom; Sara Rizk, Bristol Myers Squibb, 400 Dexter Ave N, Ste 1200, Seattle,WA 98109; Tatiana Santos, Agilent technologies, 11011 N. Torrey Pines Rd, Ste 150, LaJolla, CA 92037; Kaimei Song, AstraZeneca, 1 MedImmune Way, Gaithersburg, MD 20878; Aparna Subramanian, Kite Pharma, 1800 Stewart Street, Santa Monica, CA 90404; Jason Talia, Bristol Myers Squibb, 400 Dexter Ave N, Ste 1200, Seattle,WA 98109; Linhua Tian, Biosystems and Biomaterials Division, Material MeasurementLaboratory, National Institute of Standards and Technology(NIST), Gaithersburg, MD 20899; Jeroen van Velzen, BD Biosciences, 2350 Qume Drive, San Jose, CA 94131; Sandrine Vessillier, Medicine and Healthcare Products Regulatory Agency,National Institute for Biological Standards and Control, BlancheLane, South Mimms, Potters Bar, Hertfordshire EN6 3QG,United Kingdom; Xiuyan Wang, Memorial Sloan Kettering Cancer Center, 411 East 67th StreetS1022, New York, NY 10065

## Data Availability

The original contributions presented in the study are included in the article/**Supplementary Material**. Further inquiries can be directed to the corresponding author.
