# Supplementary material for: Standardizing a human TBMNK cell assay across instrument platforms: a new framework for producing high-quality, AI-ready reference dataset
Source: Front Immunol. 2026 Sep 28;17:1946801. doi: 10.3389/fimmu.2026.1946801 (PMC13655140; doi:10.3389/fimmu.2026.1946801)
Supplement: Supplementary file 1 [file SupplementaryFile1.pdf]

Supplemental Information

- WG2 SOP-01: Flow cytometer setup and performance characterization
- WG2 SOP-02: Compensation setup
- WG2 SOP-03: TBMNK Assay
- WG2 SOP-04: Data Analysis
- Supplemental Table 1: Number of different flow cytometer platforms used in the ILS and their manufacturers.
- Supplemental Table 2: ERF values assigned to the FC beads using reference fluorophores and under the standard filters commonly used in flow cytometer platforms and defined by NIST.
- Supplemental Table 3: ARI values assigned by NIST to the FC beads using reference fluorophores and under the filters used in the 8 FACSCanto flow cytometers by the study participants.
- Supplemental Table 4: ARI values assigned by NIST to the FC beads using reference fluorophores and under the filters used in the 5 Aurora flow cytometers by the study participants.
- Supplemental Table 5: Different flow cytometer platforms from which the FCS files have passed the QC step are equipped with red lasers with different wavelengths and different lasers for detecting PE and PE-Cy7.
- Supplemental Figure 1 - Comparison of Cytometer Detector Sensitivity (Q Value) in Standardized Spe/ARI Units for FACSCanto and Aurora Platforms.

For NIST Consortium member use only

WG2 SOP-01 Flow Cytometer Setup and Performance Characterization

1. Subtitle

Verification of Flow cytometer Performance Using Rainbow 8 Peak Beads and FC beads

2. Purpose and Introduction

The Spherotech 8 Peak Rainbow Calibration Particles (RCPs) contain a mixture of particles with different fluorescence intensities. One particle population is unstained and 7 populations contain a mixture of fluorophores that allows excitation at any wavelength from 365 to 650 nm. As a result, the Rainbow 8 Peak Beads can be used for the evaluation of most channels in the flow cytometer, including the calibration of the intensity scale in unites of statistical photoelectrons (Spe). Eight different preparations of BD Biosciences FC beads, each labeled with one specific fluorophore and assigned with an ERF (equivalent reference fluorophore) value, are used to calibrate the fluorescence intensity scales in units of ERFs. This SOP is used to prepare for the calibration of the fluorescence response, in units of Spe and ERF, and to estimate the background (B, in units of Spe and ERF) and the detection efficiency (Q, units of Spe/ERF), which can be used to estimate the limit of detection and to predict instrument performance in resolving particles of different brightness.

3. Reagents and Materials

Name/Material Code	Vendor	Catalog#<br>Lot/Batch #	Storage Conditions	Reconstitution	Supplied by
Rainbow 8 Peak Calibration Particles RCP-30-5A	Spherotech	RCP-30-5A	2-8°C, protected from light	See below	NIST
FC-FITC	BD Biosciences	661615<br>2046553	2-8°C, protected from light	See below	NIST
FC-PerCP-Cy5.5	BD Biosciences	661619<br>2101886	2-8°C, protected from light	See below	NIST
FC-PE	BD Biosciences	661616<br>2061631	2-8°C, protected from light	See below	NIST
FC-PE-Cy7	BD Biosciences	661617<br>2073743	2-8°C, protected from light	See below	NIST
FC-APC	BD Biosciences	661620<br>2073756	2-8°C, protected from light	See below	NIST
FC-APC-Cy7	BD Biosciences	661622<br>2070363	2-8°C, protected from light	See below	NIST
FC-V450	BD Biosciences	661623<br>2115671	2-8°C, protected from light	See below	NIST
FC-V500-C	BD Biosciences	661624<br>1348847	2-8°C, protected from light	See below	NIST

FC Beads Reconstitution Buffer	BD Biosciences		2-8°C, protected from light	See below	NIST
Milli Q Water					

3.1. Supplies

- Staining Tubes: 5 mL Polystyrene Round-Bottom Tubes (BD Falcon Cat. No. 352008 or equivalent) or Microwell Plates (Sarstedt Cat No 82.1583, Corning Cat No 351172, or equivalent)
- Pipet tips [P1000]
- Sterile serological pipette (1mL)

3.2. Equipment

- Pipettor P1000
- Pipette-aid
- Flow Cytometer

3.3. Reagent Preparation

Rainbow 8 Peak Calibration Particles

1. Add 2-3 drops of Rainbow 8 Peak Calibration Particles to 1 mL of Milli Q Water.

FC Beads

2. Add 0.5 mL of FC bead reconstitution buffer to a FC bead tube and vortex gently for 20 second.

4. Procedure

4.1. Instrument Setup

1. Perform the daily instrument QC & calibration per your institution's SOP (Note: cytometer fluorescence detector gain/voltage and laser power settings are the same as those used for immunophenotyping per laboratory SOP).

Turn off compensation if it is not already off after daily QC.

Note for spectral cytometers: *data will be acquired as raw and not an unmixed data file.*

#1 Recommendation: The detector gain settings used for immunophenotyping are the preferred settings (baseline settings), however the three conditions also have to be met: 1) at least 5 fluorescent Rainbow bead populations of the 8-peak beads are distinguishable for all 8 fluorescence channels; 2) all 8 fluorescent FC beads are on scale in their respective fluorescence channel; 3) a set of bead compensation controls, either AbC total antibody compensation kit plus pacific blue beads and Aqua beads, or Versacomp antibody capture bead kit plus pacific blue beads and Aqua beads (see SOP-2 for their preparations), are on scale. Final detector gain settings require that the fluorescent FC bead population and compensation beads are all on scale with 5 distinguishable Rainbow bead populations shown in each fluorescence channel. If necessary, reduce detector gains to ensure all three conditions are met.

#2 Recommendation: If there is not a setting used for routine immunofluorescence analysis for human blood phenotyping, please take the following steps: 1) adjust the detector gains so that the brightest population of the 8-peak Rainbow beads is on scale; 2) decrease or increase the detector's gain settings with FC beads to ensure all FC beads are on scale; 3) double check the detector gains with a set of bead compensation controls, either AbC total antibody compensation kit plus pacific blue beads and Aqua beads, or Versacomp antibody capture bead kit plus pacific blue beads and Aqua beads (see SOP-2 for their preparations), are on scale. Again, the final detector gain settings require that the fluorescent FC bead population and compensation beads are all on scale with 5 distinguishable Rainbow bead populations shown in each fluorescence channel.

Example plots of 8-peak Rainbow beads, FC beads, and Versacomp antibody capture beads plus pacific blue- and Aqua-coated beads are shown in Figure 1, Figure 2, and Figure 3 in Appendix A, respectively.

Notes:

1) A set of bead compensation controls should be evaluated to optimally set up gain settings for the entire study. The fluorescence intensities of all 8 bead compensation controls are higher than the respective cell-based compensation controls. In particular, the pacific blue- and Aqua-coated beads are highly fluorescent. Though it is not mandated to acquire the set of bead compensation controls, the bead compensation controls stored in dark in 0-4 °C refrigerator are stable and can be used on the second day and monitor the consistency of the compensation matrix determined (also see Note #2 below).

2) The 8-peak Rainbow beads serve as the monitoring beads for the day-to-day instrument stability under the optimized gain settings. An acceptable range of %CV is dependent of fluorescence channel, e.g., 1.4% for FITC channel and 3.4% for PE-Cy7 channel and should be established. Participant can choose one of middle fluorescence intensity populations of the 8-peak beads as the monitoring bead population to define your instrument tolerance range of the variability in Day 1 and day 2, e.g., CV of 2% for FITC channel, when the same compensation bead controls, e.g., Versacomp beads plus PB- and Aqua-coated beads, are run and the compensation matrixes are obtained and compared. Only under the acceptable range of variability, no new compensation controls need to be run on different experimental days.

3) FC beads after reconstitution with a 0.5 mL of the reconstitution buffer can be run for a few tests and are stable for at least two days in dark in 0-4 °C refrigerator.

Note for spectral cytometers: *Altering individual detector settings with spectral cytometers is not recommended. Instead, alteration of all detector channels should be made through "All Channels %" found in instrument control of Aurora cytometers. For spectral cytometers it's highly advised to fully characterize your instrument across the commonly used parameter channels in order to establish routine detector settings for immunofluorescence analysis for immunophenotyping. [A*

bioassay manuscript on the settings for immunofluorescence analysis could serve as a reference (https://pubmed.ncbi.nlm.nih.gov/34708658)]

2. Create a WG2 Rainbow 8 Peak Acquisition Template (Figure 1).

3. Acquire the bead in setup mode at a relatively slow flow rate.

4. Note that beads are smaller than blood cells, therefore the FSC and SSC gains will need to be increased in order to place the beads on scale in the FSC vs SSC plot. Set a gate for the singlet beads population on the FSC vs SSC bivariate plot to exclude background particles/noise, and bead doublets and higher order aggregates (Figure 1).

5. Note that the Relative Channel Number of the initial dot display screen may look cluttered due to the presence of background, bead doublets and higher order aggregates. However, after setting a gate on singlet beads using their FSC vs SSC intensities, the dot display screen as well as FSC histogram (Figure 1) reflects the intensities of single beads.

   Gating on the bead population in the FSC histogram, it's desirable to resolve as many fluorescent bead populations as possible for each of the 8 fluorescence channels shown in the fluorescent histograms (Figure 1, Use pulse area, not pulse height) under the PMT/gain setting for either Recommendation #1 or #2 depending on participants' situation. Some flow cytometers may not be able to resolve all 8 intensity populations in every detector. However, there should be detectors, e.g., detectors for FITC, APC, and APC-Cy7 where all or most of the populations can be resolved (Figure 1). A minimum of 5 intensity populations is required for sensitivity determination; the mean or median, robust SD, and robust CV of these resolvable populations can be determined. Also see Figure 1 for example of Y763-PC7 (PE-Cy7) detector that does not resolve the dimmest populations but has shown 5 distinct populations for statistical analysis on cytometer performance.

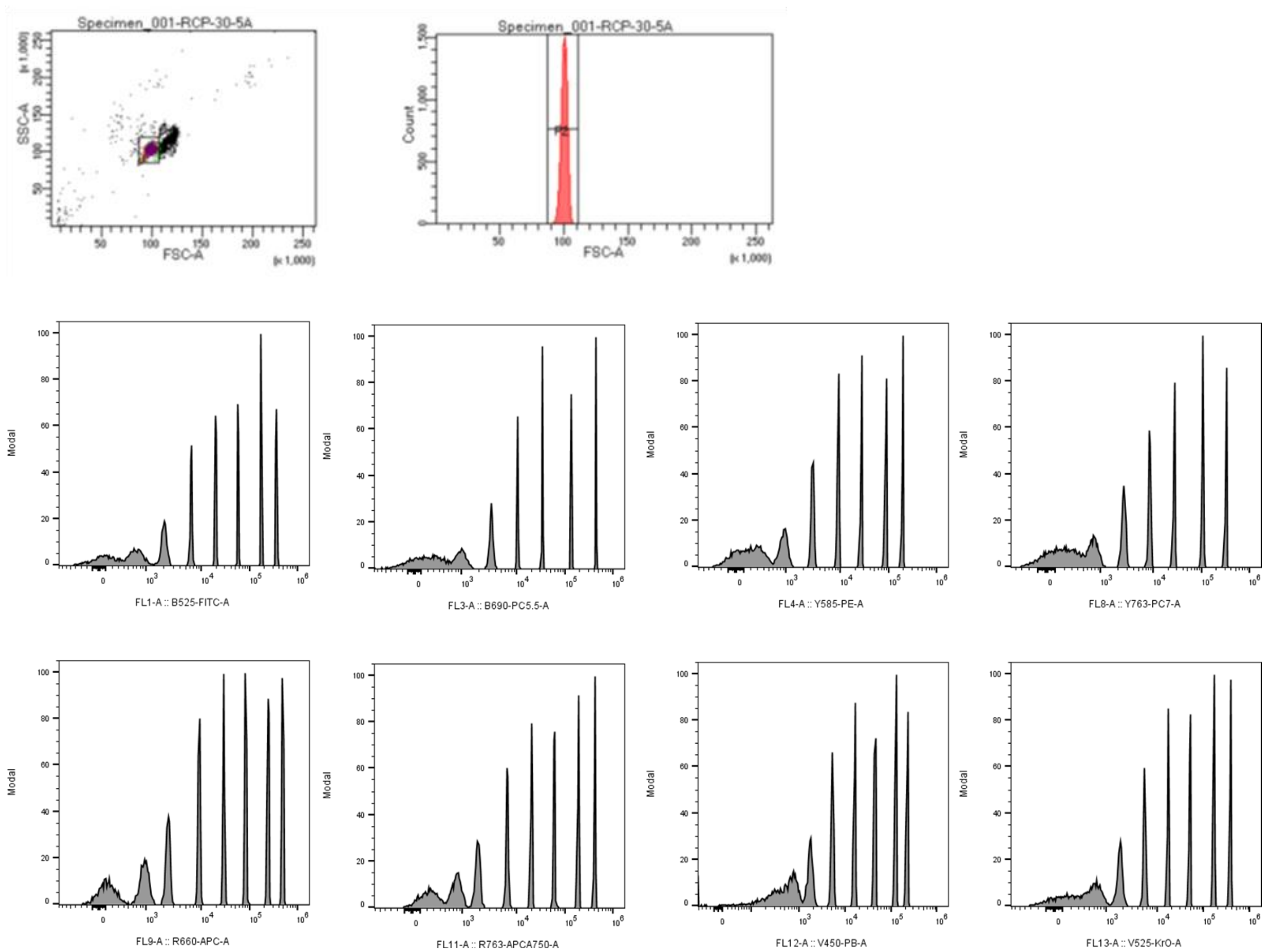

Figure 1. Example of Rainbow 8 Peak Bead Acquisition made by using a CytoFLEX LX. In this example the brightest fluorescence intensity population is on scale for all 8 fluorescence channels, however, not all the populations are resolved. The protocol requires that at least 5 intensity populations are distinguishable for the analysis of flow cytometer performance characteristics. For CytoFLEX LX: B525-FITC and B690-PC5.5 (PerCP-Cy5.5) are under 488 nm laser excitation; Y585-PE and Y763-PC7 (PE-Cy7) are with 561 nm laser excitation; R660-APC and R763-APCA750 (APC-Cy7) are under 638 nm excitation; V450-PB and V525-KrO (Aqua) are with 405 nm excitation.

6. Repeat the Step #4 above on 8 FC beads to ensure FSC and SSC gains are properly set as shown in Figure 2. The FSC and SSC gains can be adjusted if needed. Verify all 8 FC beads on the 8 different fluorescence channels are on scale in their respective histograms, and if necessary, reduce the detector voltage so the FC population is on scale. **It is essential that the FC beads and Rainbow beads are acquired under the same fluorescence gain**

settings for the 8 fluorescence channels. Example histograms for FC beads are provided in Figure 2.

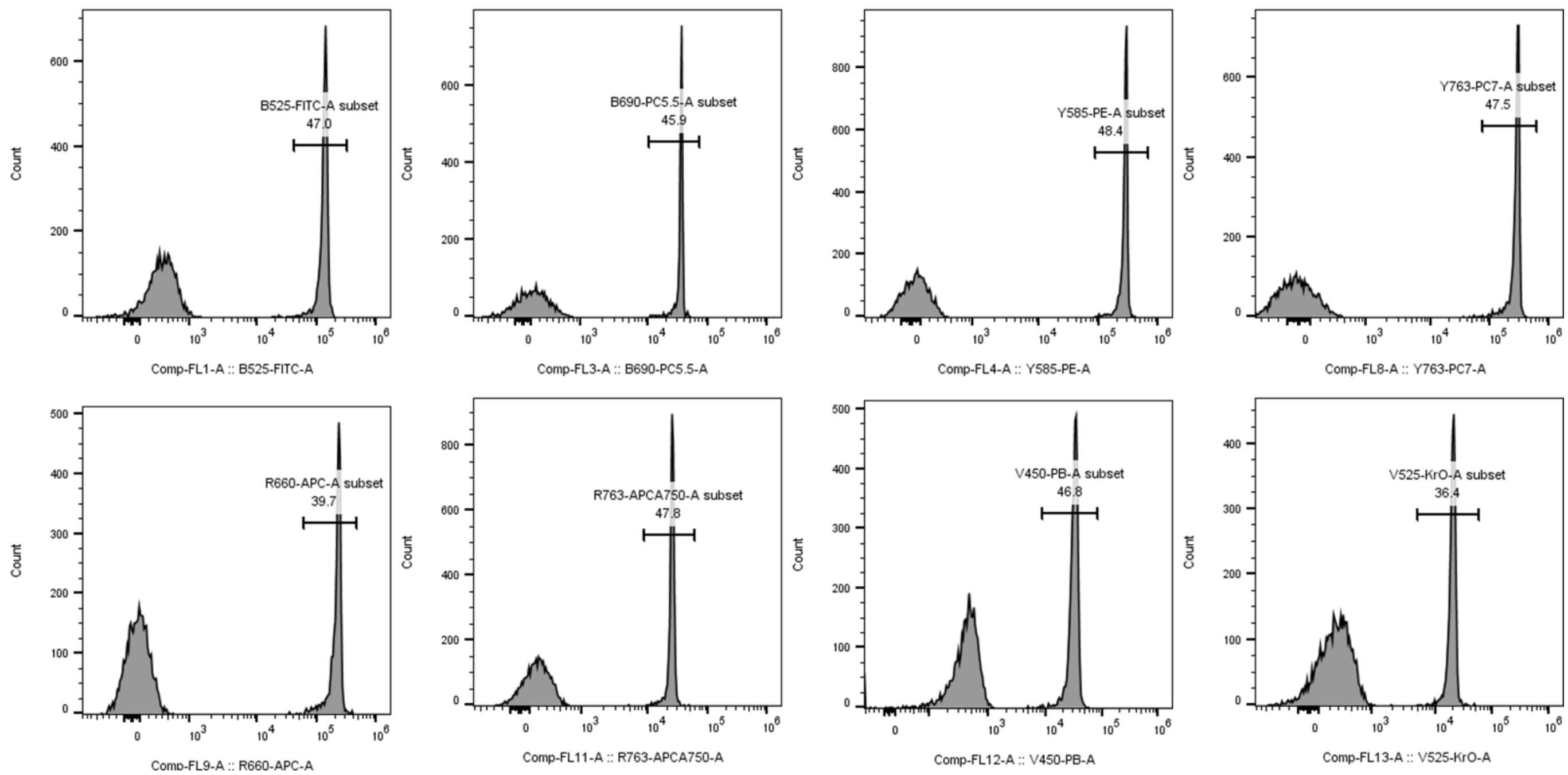

Figure 2. Example of FC Bead Acquisition made by using CytoFLEX LX. For CytoFLEX LX: B525-FITC and B690-PC5.5 (PerCP-Cy5.5) are under 488 nm laser excitation; Y585-PE and Y763-PC7 (PE-Cy7) are with 561 nm laser excitation; R660-APC and R763-APCA750 (APC-Cy7) are under 638 nm excitation; V450-PB and V525-KrO (Aqua) are with 405 nm excitation.

7. Repeat the Step #6 above on a set of bead compensation controls, e.g., Versacomp beads plus PB- and Aqua-coated beads to ensure all positive bead populations in respective fluorescence channels are on scale as shown in Figure 3 of Appendix A. Only FSC and SSC gains can be adjusted and if necessary, reduce the detector gain so the positive bead population is on scale.

8. Use the scatter gates for Rainbow Beads and the final detector gain settings to create an acquisition sample tube/well for Rainbow beads.

9. Use the scatter gates for FC beads and the final detector gain settings to create 8 acquisition sample tubes/wells for FC beads. Assure that the detector gain settings are the same for both the Rainbow and FC bead acquisition templates.

4.2. Acquisition (Exp ID-e1)

1. Open the WG2 Rainbow 8 Peak Acquisition Template (or Experiment workflow) and 9 sample tubes with local file names for Rainbow 8-peak beads and 8 different FC beads.

2. Acquire a minimum of 20,000 events in the singlet/major population gate, e.g., P2 gate in Figure 1 and 8 fluorescent FC bead gates in Figure 2.

4.3. Local Filename Entry on the WG2 Instrument Reporting Spreadsheet

1. Open the WG2 Reporting Workbook and select the “Instrument” Tab. Verify that the Study ID, Site ID, Instrument ID and Protocol ID at the top of the Spreadsheet describe the current execution of the protocol. See Figure 4 for example. One Workbook is required for each instrument.

2. For each FCS file collected (9 total files) find the corresponding material code row using the terms in “Column C”. See Figure 4.

3. Identify the material associated “Get Local Filename” button in Column O to identify the local FCS file location. See Figure 4.

4. Press the button and use the file dialog box to identify the local FCS filename corresponding to the files collected in section 4.2.

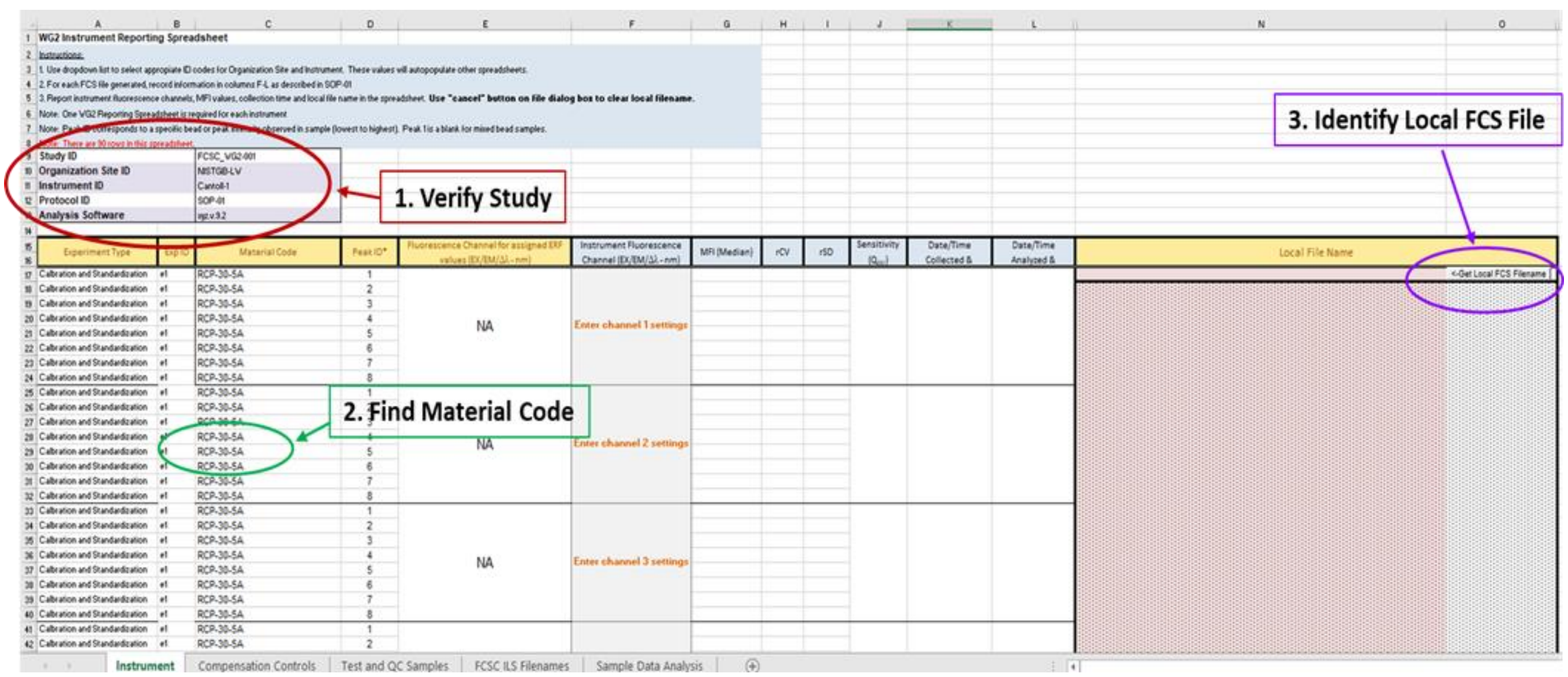

Figure 4. WG2 Instrument Reporting Spreadsheet.

4.4. Data Analysis and Reporting for Instrument Setup and Performance Characterization

1. Import the .fcs files of Rainbow 8-peak beads and 8 FC beads from the acquisition to a data analysis software.

2. Use the gating strategies shown in Figure 1 and 2 and create a statistical report table to obtain Median Fluorescence Intensity (MdFI), robust standard deviation (rSD), and robust coefficient of variation (rCV) for all measurable Rainbow bead peaks and fluorescent FC bead peak. Note: for Rainbow 8-peak beads, peak 8 is defined as the most fluorescent bead population and peak 1 is for the blank bead population.

3. Record MdFI values and associated rCV and rSD for bead populations/peaks in the Column 'G', Column 'H', and Column 'I', respectively, in the 'Instrument' sheet of the WG2 Reporting Spreadsheet.

4. Fill in information on the instrument fluorescence channels, dates for data acquisition and analysis, and operator(s) in Columns 'F', 'K', and 'L', respectively in the 'Instrument' sheet.

5. Attachments

1. Spherotech Rainbow 8 Peak Calibration Particles
2. BD FC Bead Information

Appendix A: Plots of a set of bead compensation controls under the same detector gain setting for 8-peak Rainbow beads (Figure 1) and FC beads (Figure 2)

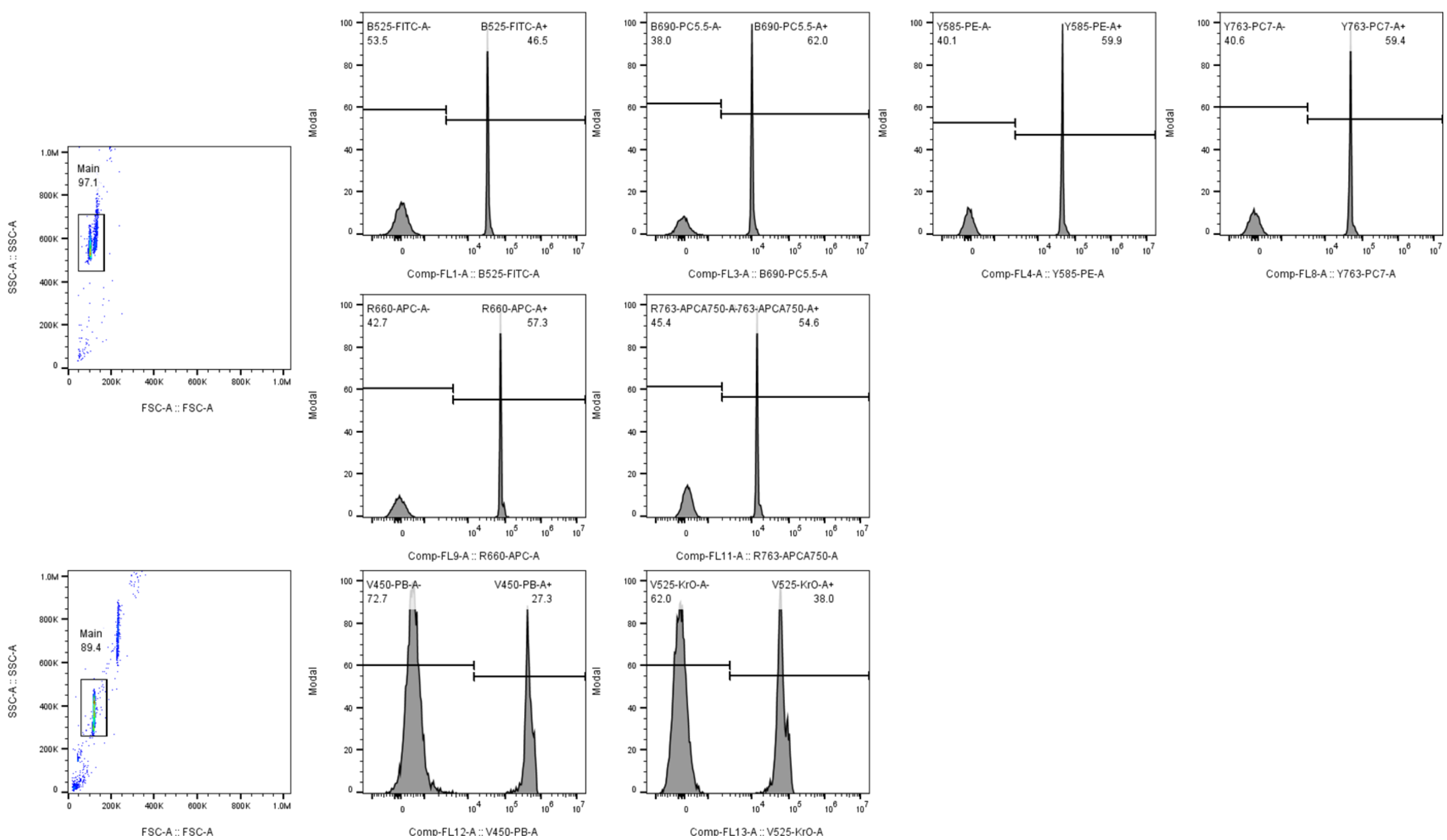

Figure 3. Example plots of Versacomp antibody capture beads (7 plots on the upper rows) plus pacific blue- and Aqua-coated beads (3 plots on the bottom row) acquired by using CytoFLEX LX. **Note**: the sizes of Versacomp antibody capture beads and Pacific blue-/Aqua- beads are different, and hence the gains for FSC and SSC need to be adjusted for proper detection (See the respective plots of FSC-A vs. SSC-A).

For NIST Consortium member use only

WG2 SOP-02 Compensation Setup

1. Overview

This Standard Operating Procedure (SOP) describes the creation of the spectral compensation matrices to be used for Working Group 2 (WG2) Inter-Laboratory Study (ILS) TBMNK Assay. Three different compensation matrices will be created as summarized in Figure 1. All three matrices will be run on each individual instrument enrolled in WG2 ILS. The single colors files acquired will be used to calculate compensation matrices that will be applied to the QC and test sample files generated per SOP-03.

Note 1: The TBMNK Assay spectral compensation will be applied post-sample acquisition, thus the creation of the compensation matrices (WG2 SOP-02) can be performed either before or after sample acquisition as described in WG2 SOP-03.

Note 2: Creation of the compensation matrices should be conducted only after the procedures described in WG2 SOP-01, Flow Cytometer Setup and Performance Characterization have been successfully completed.

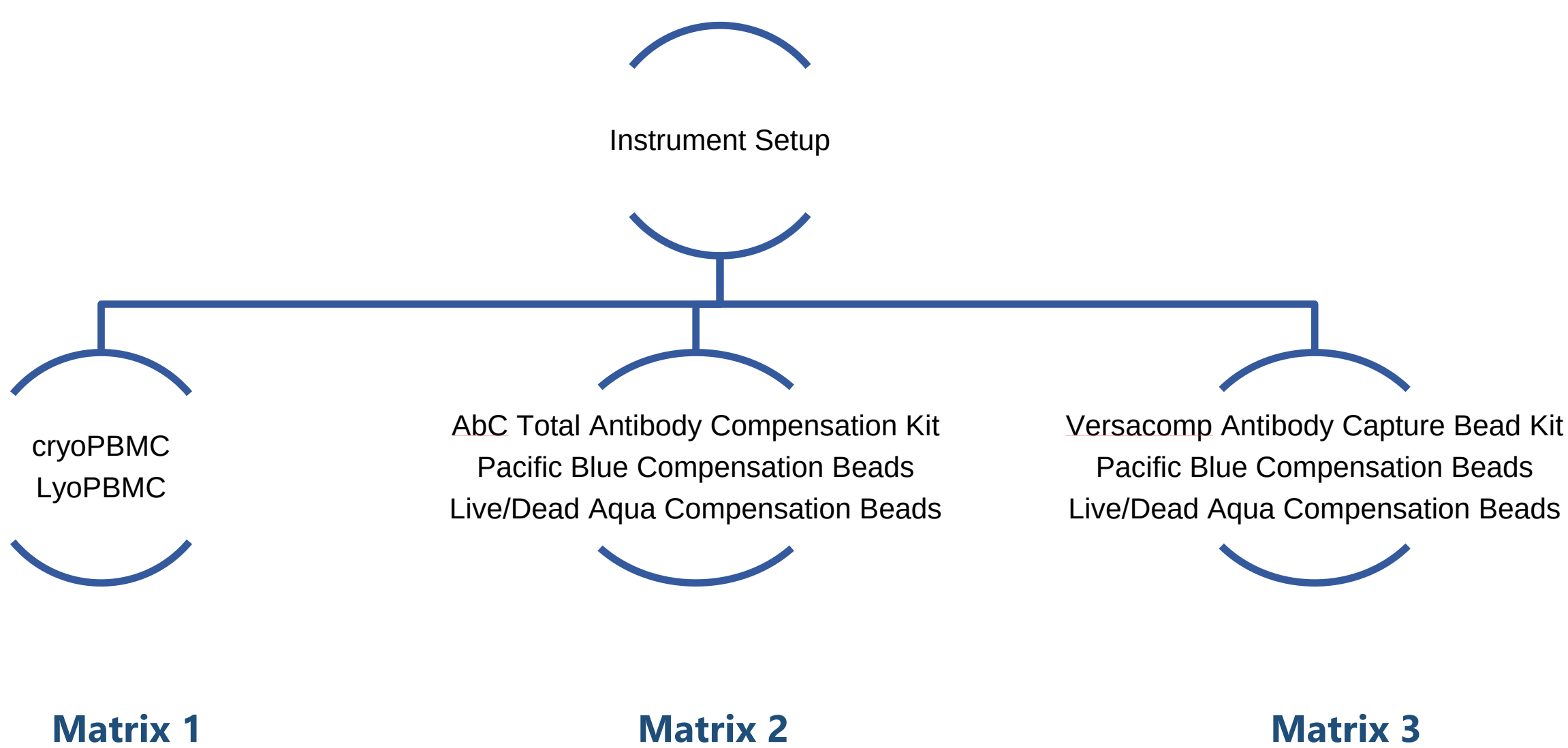

Figure 1. Compensation Workflow.

-**Compensation Matrix 1** will use both cryopreserved PBMC (cryoPBMC) -and lyophilized leukocytes (lyoLeuk), (Reference Reagent CD4 T-cells, NIBSC). Note that the cryoPBMC can be from any source, e.g., cryoPBMC from AllCells provided for the study and cryoPBMC from other sources participants possess. It is not necessary that each laboratory use the same source and/or donor for the cryoPBMC. Note also, that it would be permissible to use leftover test samples from WG2 SOP-3 and based on expected yields, this would be feasible.

-**Compensation Matrix 2** will use AbC™ Total Antibody Compensation Bead (Thermo Fisher) as well as Pacific Blue and Aqua compensation beads (Thermo Fisher). The AbC™ Total Antibody Compensation Bead Kit contains two types polystyrene microspheres- capture beads that bind all isotypes of mouse, rat, hamster, and rabbit immunoglobulin, and negative beads that have no antibody binding capacity. The Pacific Blue and Aqua Compensation Bead products were made exclusively for WG2 ILS and are ~6.0-micron microsphere surfaces directly functionalized with either Pacific Blue™ or Aqua dye. These microspheres will be used to setup compensation for the Annexin Pacific Blue™ and Aqua LIVE/DEAD products for Compensation Matrix 2 and Compensation Matrix 3.

-**Compensation Matrix 3** will use VersaComp antibody capture beads (Beckman Coulter Life Sciences) as well as Pacific Blue and Aqua compensation beads (Thermo Fisher). The VersaComp Antibody Capture Bead Kit contains two vials of 3.0-3.4 µm beads in suspension at a concentration of approximately 1×10^7 particles/mL. VersaComp Antibody Capture Negative Beads act as a negative control that do not bind fluorochrome-conjugated antibodies. VersaComp Antibody Capture Positive Beads contains beads coated with an IgG-binding agent that bind all isotypes of mouse, rat, hamster, and rabbit immunoglobulin. The Pacific Blue™ and Aqua compensation beads products as described above will also be used with Compensation Matrix 3.

2. Safety

2.1. General

- Follow appropriate biosafety procedures per your institution's SOPs and Policies.
- Safety precautions for Universal Blood precautions should be used under Biological Safety Level-2 (BSL-2) conditions (see 29 CFR 1910.1030, Bloodborne Pathogens).
- Wear appropriate personal protective equipment at all times. Gloves, safety glasses or goggles, and a laboratory coat are required personal protective equipment when handling human biological fluids including human blood (see 29 CFR 1910.1030, Bloodborne Pathogens).

2.2. Liquid Nitrogen Handling

- Cryogloves/waterproof thermal insulated gloves: Hands should be protected with waterproof thermal insulated gloves that can be quickly removed if LN2 is spilled on them. Cryogloves/waterproof thermal insulated gloves are not intended for submerging hands into LN2.
- Body must be protected with pants, lab coats, and closed-toe shoes.
- Safety goggles: Eyes are sensitive to the extreme cold of LN2 and its vapors.
- Chemical splash goggles should be used when handling LN2 and when handling cryovials in LN2.

2.3. Sodium Azide

Some reagents contain sodium azide. Sodium azide under acid conditions yields hydrazoic acid, an extremely toxic compound. Azide compounds should be flushed with running water while being discarded. These precautions are recommended to avoid deposits in metal piping in which explosive conditions can develop. If skin or eye contact occurs, wash excessively with water.

3. Reagents and Materials

3.1. Reagents

Table 2. Compensation Materials

Compensation Matrix	Name	Vendor	Catalog #	Expiry	Storage Conditions	Reconstitution
1	Cryopreserved PBMC	Any source	NA	NA	Liquid nitrogen vapor phase	See WG2 SOP-03 section 4.3.1
	LyoLeuk (Reference Reagent CD4 T-cells)	NIBSC	15/270	Per certificate of analysis	-20°C or below	See section 4.3.1
2	Pacific Blue™ Compensation Bead	ThermoFisher Scientific	NA/Custom Product	6 months after receipt	2-8°C	Ready to use
	Aqua Compensation Bead	ThermoFisher Scientific	NA/Custom Product	6 months after receipt	2-8°C	Ready to use
	AbC™ Total Antibody Compensation Bead Kit	ThermoFisher Scientific	A10497	6 months after receipt	2-8°C	Ready to use
3	VersaComp Antibody Capture Bead Kit	Beckman Coulter Life Sciences	B22804	As indicated on product vial	2-8°C	Ready to use.

Table 3. Monoclonal Antibodies

Vendor	Catalog #	Marker	Format	Clone	Storage Conditions	Reconstitution	Supplied by
BioLegend	900005606	CD45	PCP-Cy5.5	2D1	2-8°C, protected from light	Ready to use	NIST
BioLegend	900005607	CD3	FITC	SK7	2-8°C, protected from light	Ready to use	NIST
BioLegend	900005609	CD14	APC	M5E2	2-8°C, protected from light	Ready to use	NIST
BioLegend	900005611	CD16	PE	3G8	2-8°C, protected from light	Ready to use	NIST
BioLegend	900005613	CD4	PE-Cy7	SK3	2-8°C, protected from light	Ready to use	NIST

BioLegend	900005606	CD8	APC-Cy7	SK1	2-8°C, protected from light	Ready to use	NIST

Table 4. Other Reagents

Name	Vendor	Catalog #	Storage Conditions	Reconstitution	Supplied by
PBS without Ca^{++}/Mg^{++}	GIBCO/ any	10010-023 or equivalent	Unopened RT (18-25°C)<br>Opened 2-8°C	Ready to use	Lab to purchase
FBS	Any	N/Ap	Unopened -20°C<br>Opened at 2-8°C	Ready to use	Lab to purchase
Ethanol, e.g. 200 proof	Any	N/Ap	RT (18-25°C)	See below	Lab to purchase
$CaCl_2$	Any	N/Ap	RT (18-25°C)	See below	Lab to purchase
LIVE/DEAD™ Fixable Aqua Dead Cell Stain	Thermo Fisher	L34957	-20°C	See below	NIST
Pacific Blue™ Annexin V	Thermo Fisher	A35136	-2-8 °C	Ready to use	NIST

3.2. Supplies

- Staining Tubes: 5 mL Polystyrene Round-Bottom Tubes
- Pipet tips (P10, P20, P100, P200, P1000)
- 15 mL and 50 mL polypropylene conical tubes with screw cap lids
- 96-well plates, per your institution's SOP (for testing labs that stain in plates, only)
- Sterile serological pipettes (e.g., 2 mL, 5 mL, 10 mL, 25 mL)

3.3. Equipment

- Pipettors P10, P20, P100, P200, P1000
- Pipette-aid
- Water Bath
- Centrifuge
- Biosafety Cabinet
- Cytometer (as described in WG2 Study Plan)

3.4. Reagent Preparation

70% Ethanol

Prepare per your institution's SOPs.

Staining Buffer

Prepare a solution of 1X PBS ≥3.5 mM CaCl2, containing a final volume of 2% FBS, pH 7.4, per your institution's SOPs.

LIVE/DEAD™ Fixable Aqua Dead Cell Stain

Note this kit contains:

- Component A – Aqua dye
- Component B - Dimethylsulfoxide (DMSO), anhydrous

Bring 1 vial of fluorescent reactive dye (Component A) and 1 vial of anhydrous DMSO (Component B) to RT before removing the caps.

Add 50 µL of DMSO to the vial of reactive dye. Mix well and visually confirm that all of the dye has dissolved. Keep reconstituted dye solution in dark at room temperature and use it within 2 hours of reconstitution.

4. Procedure

4.1. Instrument Setup

Use the instrument setting from SOP-1 except that FSC and SSC will be adjusted as appropriate for different compensation matrices.

4.2. Worklist Creation

Create a worklist containing sample tubes and a local file naming scheme to save files described in the 'Compensation Controls' of the WG2 Reporting Spreadsheet.

4.3. Compensation Materials Preparation and Staining Procedure

Stained beads or cells will be acquired as compensation samples per Tables 5-8. In total 22 files will be generated.

4.3.1. Compensation Matrix 1

Lyophilized PBMC, CD4 T-cells (lyoLeuk) (Exp ID: e1)

Proper aseptic and biohazard technique should be used when handling and manipulating cells. Perform in biosafety cabinet.

- Remove 2 ampoules out of storage, place in a tube holder and allow to adjust to RT, break ampoule seal.
- Pipette 1 mL sterile distilled water in each of 2 lyophilized PBMC vials (each lyophilized PBMC vial contains 1.5 x 10^6 PBMC) and allow 15-30 min for rehydration.
- Mix cell suspension well by gently pipetting up and down and transfer 1 mL of lyophilized PBMC to a capped tube.
- Add 3 mL of PBS to each tube.
- Centrifuge at RT for 7 minutes at 350 × *g*.
- Aspirate the supernatant. Resuspend the cell pellet in 200 µL of PBS in one tube and in 200 µL of the staining buffer in the second tube.
- Add 100 µL cell suspension in PBS in previous step, to the Aqua compensation tube, comp tube 7 (Table 5) and then 900 µL PBS to make up 1 mL of cell suspension.
- Add 100 µL cell suspension in the staining buffer in previous step, to the Annexin V Pacific Blue compensation tube, comp tube 8 (Table 5).
- Add appropriate dilution of LIVE/DEAD™ Fixable Aqua Dead Cell Stain or Pacific Blue™ Annexin V (as indicated in Table 5). Take care to deposit the stain to the cell suspension. Vortex immediately.
- Incubate at RT for 30 min, protected from light.
- Add 3 mL of PBS to each tube.
- Centrifuge for 7 minutes at 350 × *g*.
- Aspirate the supernatant. Resuspend the cell pellet.
- Add 3 mL of PBS to each tube.

- Centrifuge for 7 minutes at 350 × *g*.
- Aspirate the supernatant. Resuspend the cell pellet.
- Add 400 µL staining buffer and then add 100 µL cells resuspended in either PBS or staining buffer from the step bullet #6 above, serving as unstained cells.
- Note: Lyophilized PBMCs (lyoLeuk) are dead cells and are stained with Aqua or Pacific Blue 100%, and therefore unstained cells are needed to serve as negative cell controls. During acquisition/analysis, participants need to identify more approporiate cell population, lymphocyte or monocyte, that gives better separation of the fluorescence peaks from positive and negative cell populations.

cryoPBMC (Exp ID: e1)

Proper aseptic and biohazard techniques should be used when handling and manipulating cells. Perform in biosafety cabinet.

- Refer to WG2 SOP-03 for instructions on cell thawing (In the cell thawing process, participants need to perform cell counting).
- Resuspend thawed cells in an appropriate volume of Staining Buffer to achieve cell concentration of 5 x 10^6/mL.
- Label tubes as indicated in Table 5.
- Add 100 µL cell suspension to each compensation tube.
- Add appropriate dilution of individual mAb (as indicated in Table 5). Take care to deposit the mAb to the cell suspension. Vortex immediately.
- Incubate at RT for 30 min, protected from light.
- Add 3 mL of PBS to each tube.
- Centrifuge for 7 minutes at 350 × *g*.
- Aspirate the supernatant. Resuspend the cell pellet.
- Add 3 mL of PBS to each tube.
- Centrifuge for 7 minutes at 350 × *g*.
- Aspirate the supernatant. Resuspend the cell pellet.
- Add 400 µL staining buffer and then add 100 µL unstained cells.
- Note: Only comp tube #2 (CD45 PerCP-Cy5.5) and tube #5 (CD14 APC) absolutely require the addition of unstained cells.

Acquire Compensation Matrix 1 Samples (cryoPBMC and lyoLeuk) on the flow cytometer as described in Section 4.5.

4.3.2. Compensation Matrix 2

AbC™ Total Antibody Compensation Bead (Exp ID: e2)

- Label tubes as indicated in Table 6.
- Completely resuspend the AbC™ Total Compensation capture beads (Component A) and negative beads (Component B) by gently vortexing for 10 seconds before use.
- Add 1 drop of AbC™ Total Compensation capture beads (Component A) to each tube.
- Add appropriate dilution of individual mAb (as indicated in Table 6). Take care to deposit the mAb to the cell suspension. Vortex immediately.
- Incubate for 15 minutes at RT, protected from light.
- Add 3 mL of the staining buffer to each tube.
- Centrifuge for 7 minutes at 250 × *g*.
- Decant the supernatant and resuspend the beads in 500 µL of staining buffer.
- Add one drop of negative beads (Component B) to each stained tube and mix well.
- Acquire on the instrument within 30 minutes of preparation.

- Vortex the thoroughly (at low speed) immediately prior to acquisition.

Acquire Compensation Matrix 2 AbC™ Total Antibody Compensation Bead on the flow cytometer as described in Section 4.5.

4.3.3. Compensation Matrix 3

VersaComp antibody capture beads (Exp ID: e3)

- Ensure proper resuspension of the individual kit components by vortexing vials prior to use.
- Label tubes as indicated in Table 7.
- Add one drop of negative beads and one drop of positive beads to each tube.
- Add mAb to the designated tube as indicated in Table 7. Take care to deposit the antibody directly to the bead suspension. Vortex immediately.
- Incubate at RT for 20 minutes, protected from light.
- Add 1 mL of the staining buffer to each test tube, vortex and centrifuge at 300 x *g* for 6 minutes.
- Decant the supernatant and resuspend the beads in 600 µL of staining buffer.

Acquire Compensation Matrix 3 VersaComp antibody capture beads on the flow cytometer as described in Section 4.5.

4.3.4. Pacific Blue™ and Aqua Compensation Bead Products (Exp ID: e4)

- Gently vortex the Pacific Blue™ or Aqua Compensation Bead products, and Component B from the AbC Total Antibody Compensation Bead kit (Component B) 10 seconds to completely resuspend before use.
- Label tubes as indicated in Table 8.
- Add 1 drop of the Pacific Blue™ or Aqua Compensation Bead product to the appropriate sample tube.
- Allow Pacific Blue™ or Aqua Compensation beads to sit in the tube for 5 minutes to warm to RT.
- Add 500 µL of the staining buffer to each tube.
- Add one drop of negative beads (Component B in the AbC™ Total Antibody Compensation Bead Kit) to sample tubes.
- Vortex the tubes before analyzing.
- Perform flow cytometry on the samples as described in Section 4.5.

Acquire Compensation Matrix 3 VersaComp antibody capture beads on the flow cytometer as described in Section 4.5.

4.4. Compensation Staining Maps

Table 5. Compensation Matrix-1 Staining

Experiment ID	Tube ID	Material /Bead	Antibody (fluorochrome)	Material Code	Volume of antibody to be added
e1	comp tube 1	cryoPBMC	CD3 (FITC)	FITC-cryoPBMC	5 µL
e1	comp tube 2	cryoPBMC	CD45 (PerCP-Cy5.5)	PerCP-Cy5.5-cryoPBMC	5 µL

e1	comp tube 3	cryoPBMC	CD16 (PE)	PE-cryoPBMC	5 µL
e1	comp tube 4	cryoPBMC	CD4 (PE-Cy7)	PE-Cy7-cryoPBMC	5 µL
e1	comp tube 5	cryoPBMC	CD14 (APC)	APC-cryoPBMC	5 µL
e1	comp tube 6	cryoPBMC	CD8 (APC-Cy7)	APC-Cy7-cryoPBMC	5 µL
e1	comp tube 7	lyoLeuk	live/dead fixable Aqua	AqLD-lyoLeuk	1 µL
e1	comp tube 8	lyoLeuk	Annexin V Pacific Blue	AxPB-lyoLeuk	5 µL

Table 6. AbC Total Antibody Beads Compensation Tubes and Staining volumes

Experiment ID	Tube ID	Material /Bead	Antibody (fluorochrome)	Material Code	Volume of antibody to be added
e2	comp tube 1	AbC total antibody compensation bead	CD3 (FITC)	FITC-AbCTotal	5 µL
e2	comp tube 2	AbC total antibody compensation bead	CD45 (PerCP-Cy5.5)	PerCP-Cy5.5-AbCTotal	5 µL
e2	comp tube 3	AbC total antibody compensation bead	CD16 (PE)	PE-AbCTotal	5 µL
e2	comp tube 4	AbC total antibody compensation bead	CD4 (PE-Cy7)	PE-Cy7-AbCTotal	5 µL
e2	comp tube 5	AbC total antibody compensation bead	CD14 (APC)	APC-AbCTotal	5 µL
e2	comp tube 6	AbC total antibody compensation bead	CD8 (APC-Cy7)	APC-Cy7-AbCTotal	5 µL

Table 7. VersaComp antibody capture bead Compensation Tubes and Staining volumes

Experiment ID	Tube ID	Material /Bead	Antibody (fluorochrome)	Material Code	Volume of antibody to be added
e3	comp tube 1	VersaComp antibody capture bead	CD3 (FITC)	FITC-Versacmp	5 µL
e3	comp tube 2	VersaComp antibody capture bead	CD45 (PerCP-Cy5.5)	PerCP-Cy5.5-Versacmp	5 µL

e3	comp tube 3	VersaComp antibody capture bead	CD16 (PE)	PE-Versacmp	5 µL
e3	comp tube 4	VersaComp antibody capture bead	CD4 (PE-Cy7)	PE-Cy7-Versacmp	5 µL
e3	comp tube 5	VersaComp antibody capture bead	CD14 (APC)	APC-Versacmp	5 µL
e3	comp tube 6	VersaComp antibody capture bead	CD8 (APC-Cy7)	APC-Cy7-Versacmp	5 µL

Table 8. Aqua and Pacific Blue Beads Compensation Tubes and Staining volumes

Experiment ID	Tube ID	Material /Bead	Antibody (fluorochrome)	Material Code	Volume of antibody to be added
e4	comp tube 7	Live/Dead Aqua compensation bead	live/dead fixable Aqua	AqBead	Per section 4.3.4
e4	comp tube 8	Pacific Blue compensation bead	Annexin V Pacific Blue	PBBead	Per section 4.3.4

4.5. Acquisition

- Create an acquisition template for the Compensation Matrices
- Use the gain settings for all fluorescence detectors finalized in the SOP-01. The gain settings for both FSC and SSC can be adjusted for different compensation control types to place them on scale if needed.
- Note: Because VersaComp antibody capture beads are 3-3.4 µm and both Aqua and Pacific blue compensation beads are 6 µm in diameter, the FCS and SSC gains must be adjusted for the size differences prior to acquisition of the Compensation Matrix 3.
- Acquire samples without compensation and with 15 000-20 000 events in the singlet gate
- Verify the gains setup in SOP-01 are appropriate for compensation controls. If the signal is too bright, re-stain the comp matrices with less antibody (e.g., 3-4 µL of antibody instead of 5 µL of antibody).

4.6. Local Filename Entry on the WG2 Compensation Control Reporting Spreadsheet

1. Open the WG2 Reporting Workbook and select the “Compensation Control” Tab. Verify that the Study ID, Site ID, Instrument ID and Protocol ID at the top of the Spreadsheet describe the current execution of the protocol. See Figure 2 for example. One Workbook is required for each instrument.
2. For each file collected (22 total files) find the corresponding material code row using the terms in “Column D”. See Figure 2.
3. Identify the material associated “Get Local Filename” button in Column F to identify the local file location. See Figure 2
4. Press the button and use the file dialog box to identify the local FCS filename corresponding to the files collected in Section 4.5.

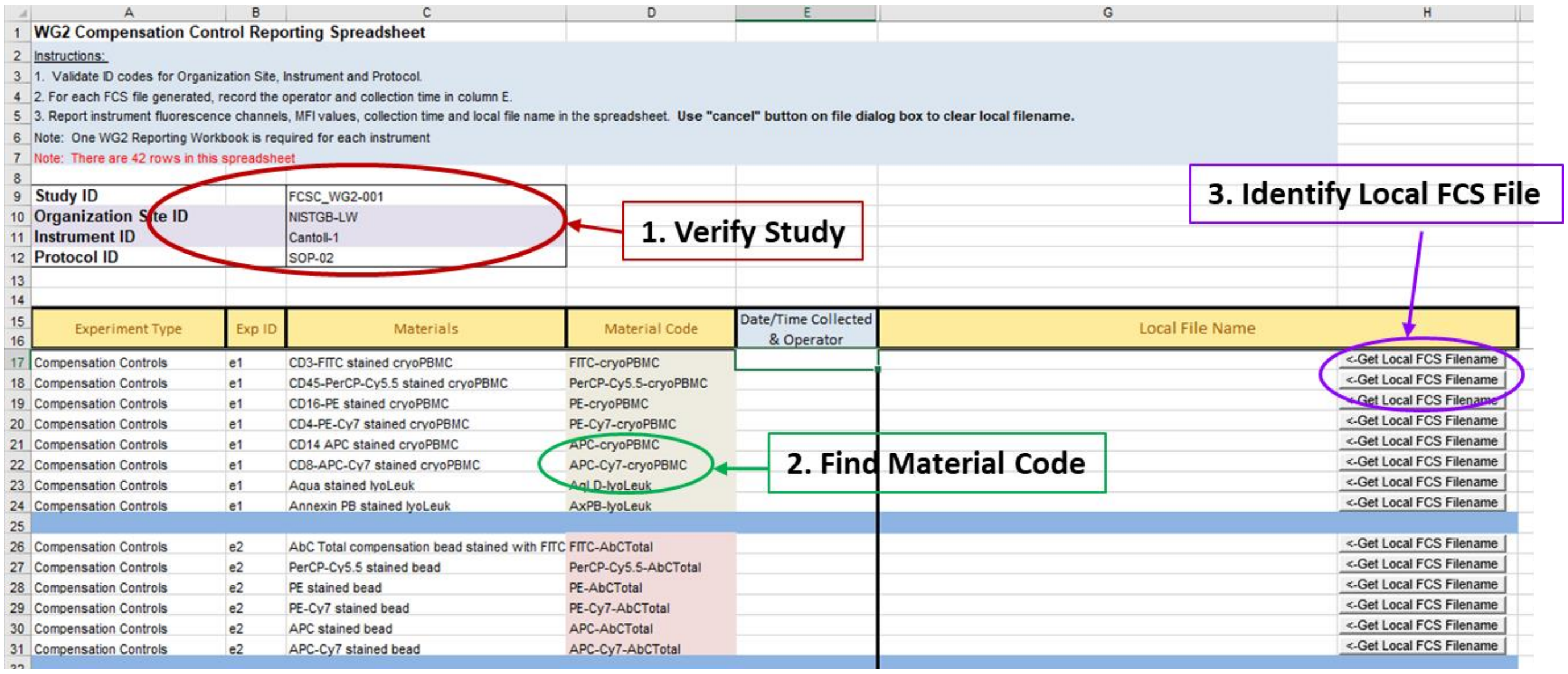

WG2 Compensation Control Reporting Spreadsheet

Instructions:
1. Validate ID codes for Organization Site, Instrument and Protocol.
2. For each FCS file generated, record the operator and collection time in column E.
3. Report instrument fluorescence channels, MFI values, collection time and local file name in the spreadsheet. **Use "cancel" button on file dialog box to clear local filename.**
Note: One WG2 Reporting Workbook is required for each instrument
Note: There are 42 rows in this spreadsheet

**Study ID**	FCSC_WG2-001
**Organization Site ID**	NISTGB-LW
**Instrument ID**	Cantoll-1
**Protocol ID**	SOP-02

Experiment Type	Exp ID	Materials	Material Code	Date/Time Collected & Operator	Local File Name	
Compensation Controls	e1	CD3-FITC stained cryoPBMC	FITC-cryoPBMC			<-Get Local FCS Filename
Compensation Controls	e1	CD45-PerCP-Cy5.5 stained cryoPBMC	PerCP-Cy5.5-cryoPBMC			<-Get Local FCS Filename
Compensation Controls	e1	CD16-PE stained cryoPBMC	PE-cryoPBMC			<-Get Local FCS Filename
Compensation Controls	e1	CD4-PE-Cy7 stained cryoPBMC	PE-Cy7-cryoPBMC			<-Get Local FCS Filename
Compensation Controls	e1	CD14 APC stained cryoPBMC	APC-cryoPBMC			<-Get Local FCS Filename
Compensation Controls	e1	CD8-APC-Cy7 stained cryoPBMC	APC-Cy7-cryoPBMC			<-Get Local FCS Filename
Compensation Controls	e1	Aqua stained lyoLeuk	AqLD-lyoLeuk			<-Get Local FCS Filename
Compensation Controls	e1	Annexin PB stained lyoLeuk	AxPB-lyoLeuk			<-Get Local FCS Filename
Compensation Controls	e2	AbC Total compensation bead stained with FITC	FITC-AbCTotal			<-Get Local FCS Filename
Compensation Controls	e2	PerCP-Cy5.5 stained bead	PerCP-Cy5.5-AbCTotal			<-Get Local FCS Filename
Compensation Controls	e2	PE stained bead	PE-AbCTotal			<-Get Local FCS Filename
Compensation Controls	e2	PE-Cy7 stained bead	PE-Cy7-AbCTotal			<-Get Local FCS Filename
Compensation Controls	e2	APC stained bead	APC-AbCTotal			<-Get Local FCS Filename
Compensation Controls	e2	APC-Cy7 stained bead	APC-Cy7-AbCTotal			<-Get Local FCS Filename

Figure 2- WG2 Compensation Control Reporting Spreadsheet.

5. Attachments

1. Information for Lyophilized Leuokcytes (Reference Reagent CD4 T-cells, NIBSC)
2. Information for Annexin Pacific Blue
3. Information for Aqua LIVE/DEAD Stain
4. Information for Pacific Blue and Aqua Coated Compensation Beads
5. Information for AbC Total Product
6. Information for VersaComp Beads

For NIST Consortium member use only

WG2 SOP-03 TBMNK Assay

1. Overview

This Standard Operating Procedure (SOP) describes the sample processing steps for the TBMNK Assay to be used for the Working Group 2 (WG2) Inter-Laboratory Study (ILS). The TBMNK Assay is a two-tube immunofluorescent panel that includes a three-color counting tube and an eight-color immunophenotyping tube (Tables 1A and 1B) . In addition to fluorescent conjugated monoclonal antibodies (mAb) each tube probes for excluding non-viable and apoptotic cells.

The scope of the WG2 ILS includes the evaluation of three Test Samples (cryopreserved PBMC) and quality control (QC) material (synthetic TBMNK cells).

This SOP describes all steps related to the processing of the Test Samples and QC materials.

NOTE 1: This procedure should be conducted only after the processes described in WG2 SOP-1, Flow Cytometer Setup and Performance Characterization has been successfully completed.

NOTE 2: Given that the compensation matrices will be applied post-acquisition, this procedure may be conducted before or after the processes described in WG2 SOP-2, Compensation Setup have been completed.

An overview of the workflow is shown in Figure 1. Briefly, Test Samples will first be stained with a viability dye (LIVE/DEAD™ Fixable Aqua). Next; an aliquot of cells will be stained in singlicate with CD45 and Annexin V in a Trucount™ tube using a No Wash method (Table 1A). The remaining cells will be stained in triplicate with fluorophore labeled mAb and Annexin V followed by a washing step (Table 1B). For all three Test Samples the Counting Tube will be prepared in duplicate while the Immunophenotyping Tube will be prepared in triplicate. Nine FMO tubes will be prepared in singlicate from a single Test Sample. It is not necessary to prepare FMO tubes for each Test Sample (Table 5).

NOTE 3*:* A single set of FMO tubes from one of the donor cryoPBMCs will be sufficient.

NOTE 4*:* Left-over cryoPBMC from the Test Samples can be used for preparation of cell-based compensation controls as described in the SOP-02.

The synthetic TBMNK cells (TruCytes TBMNK) will serve as the QC material and will be processed as described above with the exception that the viability and Annexin V staining and FMO controls will not be included; and the full panel will be stained in singlicate (Figure 1).

All samples may be processed on the same day or over a period of different testing days so long as the span of the testing days does not exceed two weeks. Please review the notes in SOP-01 for instrument gain settings across different experimental days.

A local data file naming scheme to be used for saving the .fcs files should be developed ahead of time. The process for naming files to for transfer to NIST and the file transfer process are described in Sections 4.6 and 4.7.

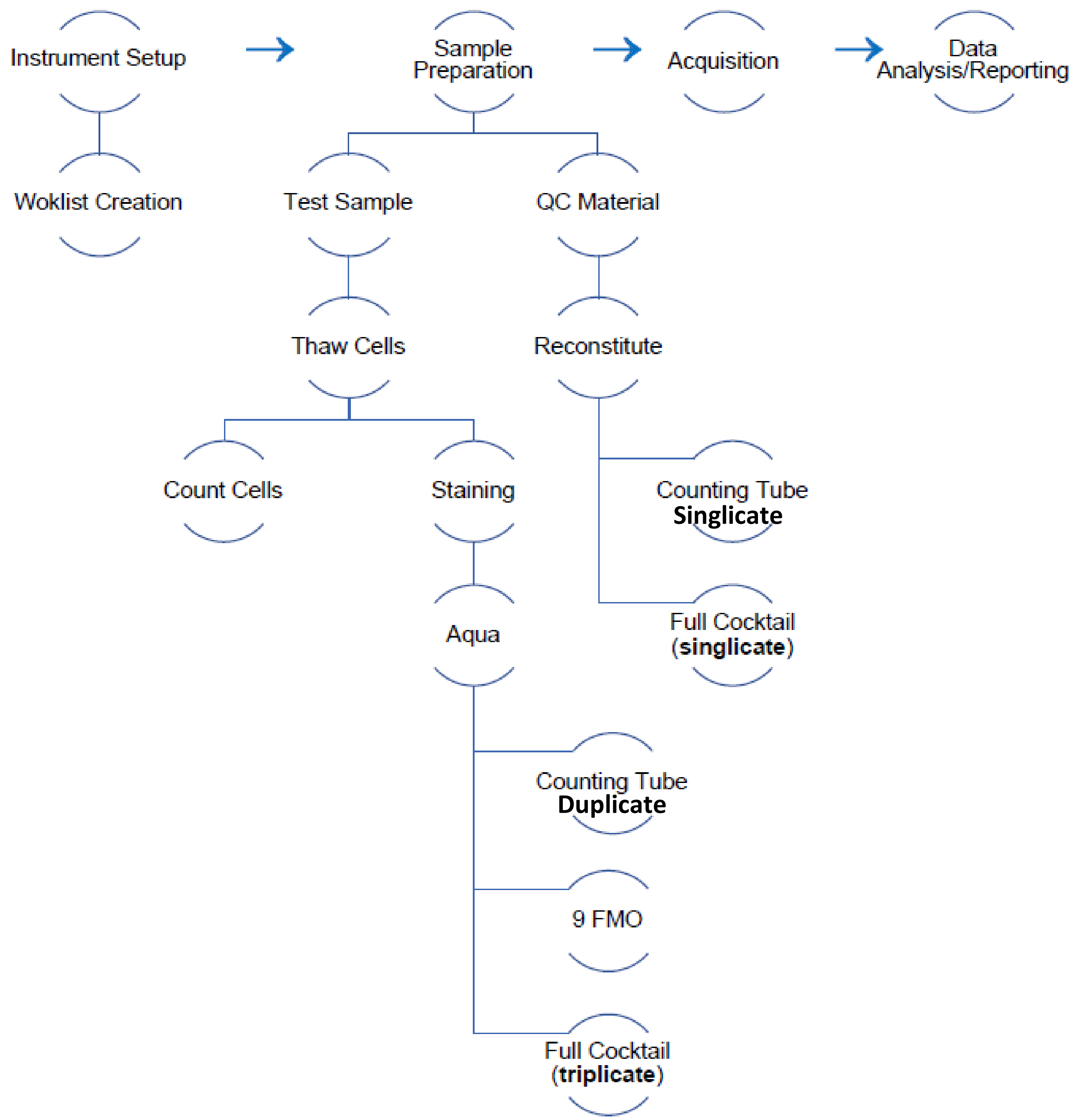

Figure 1. Working Group 2 Inter-Laboratory Study TBMNK Assay Workflow

Table 1A. TBMNK Panel Configuration- Counting Tube

Laser	Violet	Violet	Blue	Blue or Yellow Green	Blue	Blue or Yellow Green	Red	Red
	405mn	405mn	488nm	488nm	488nm	488nm	640nm	640nm
Approximate Filters	448/45	528/45	527/32	586/42	700/54	783/56	660/10	783/56
Probe	Pacific Blue/ Annexin V	Aqua/ cellular amines	-	-	PerCP-Cy5.5	-	APC	-
Marker	Phospha-tidylserine	Non-viable cells	-	-	CD45	-	Counting Beads*	-
Clone	N/Ap	N/Ap	-	-	2D1	-	N/Ap	-

*Counting beads can be detected in many different channels.

Table 1B. TBMNK Panel Configuration- Immunophenotyping Tube

Laser	Violet	Violet	Blue	Blue or Yellow Green	Blue	Blue or Yellow Green	Red	Red
	405mn	405mn	488nm	488nm	488nm	488nm	640nm	640nm
Approximate Filters	448/45	528/45	527/32	586/42	700/54	783/56	660/10	783/56
Probe	Pacific Blue/ Annexin V	Aqua/ cellular amines	FITC	PE	PerCP-Cy5.5	PE-Cy7	APC	APC-Cy7
Marker	Phospha-tidylserine	Non-viable cells	CD3	CD16/CD56	CD45	CD4	CD19/CD14	CD8
Clone	N/Ap	N/Ap	SK7	3G8/HCD56	2D1	SK3	SJ25C1/M5E2	SK1

2. Safety

2.1. General

- Follow appropriate biosafety procedures per your institution's SOPs and Policies.
- Safety precautions for Universal Blood precautions should be used under Biological Safety Level-2 (BSL-2) conditions (see 29 CFR 1910.1030, Bloodborne Pathogens).
- Wear appropriate personal protective equipment at all times. Gloves, safety glasses or goggles, and a laboratory coat are required personal protective equipment when handling human biological fluids including human blood (see 29 CFR 1910.1030, Bloodborne Pathogens).

2.2. Liquid Nitrogen Handling

- Cryogloves/waterproof thermal insulated gloves: Hands should be protected with waterproof thermal insulated gloves that can be quickly removed if LN2 is spilled on them. Cryogloves/waterproof thermal insulated gloves are not intended for submerging hands into LN2.
- Body must be protected with pants, lab coats, and closed-toe shoes.
- Safety goggles: Eyes are sensitive to the extreme cold of LN2 and its vapors.
- Chemical splash goggles should be used when handling LN2 and when handling cryovials in LN2.

3. Reagents and Materials

3.1. Reagents

Table 2. Test Samples and QC Material

Name	Description	Vendor	Cat #	Storage Conditions	Preparation	Supplied by
Cryopreserved PBMC*	Test Sample	AllCells	LP, CR, MNC, 10M<br>Donor #1: 3063593<br>Donor #2: 3066774<br>Donor #3: 3069118	Liquid nitrogen vapor phase	See below	NIST
TruCytes TBMNK	QC	Slingshot	SSB-14-A	-20°C	See below	NIST

*Donor # of cryoPBMC shall be consistent between experiment(s) and study reporting spreadsheet.

Table 3. Monoclonal Antibodies

Vendor	Catalog #	Marker	Format	Clone	Storage Conditions	Reconstitution	Supplied by
BioLegend	900005606	CD45	PCP-Cy5.5	2D1	2-8°C, protected from light	Ready to use	NIST
BioLegend	900005607	CD3	FITC	SK7	2-8°C, protected from light	Ready to use	NIST
BioLegend	900005608	CD19	APC	SJ25C1	2-8°C, protected from light	Ready to use	NIST
BioLegend	900005609	CD14	APC	M5E2	2-8°C, protected from light	Ready to use	NIST
BioLegend	900005610	CD56	PE	HCD56	2-8°C, protected from light	Ready to use	NIST
BioLegend	900005611	CD16	PE	3G8	2-8°C, protected from light	Ready to use	NIST
BioLegend	900005613	CD4	PE-Cy7	SK3	2-8°C, protected from light	Ready to use	NIST
BioLegend	900005606	CD8	APC-Cy7	SK1	2-8°C, protected from light	Ready to use	NIST

Table 4. Other Reagents

Name	Vendor	Catalog #	Storage Conditions	Reconstitution	Supplied by
RPMI-1640	Any	N/Ap	2-8°C	Ready to use	Testing Laboratory to purchase
PBS without Ca^{++}/Mg^{++}	GIBCO/ any	10010-023 or equivalent	Unopened RT (18-25°C)<br>Opened 2-8°C	Ready to use	Testing Laboratory to purchase
FBS	Any	N/Ap	Unopened -20°C<br>Opened at 2-8°C	Ready to use	Testing Laboratory to purchase
Penicillin-Streptomycin (10,000 U/mL)	Any	N/Ap	Unopened at -20°C<br>Opened at 2-8°C	Ready to use	Testing Laboratory to purchase
Trypan Blue Solution 0.2%	Any	N/Ap	2-8°C	Ready to use	Testing Laboratory to purchase
Ethanol, e.g. 200 proof	Any	N/Ap	RT (18-25°C)	See below	Testing Laboratory to purchase
$CaCl_2$	Any	N/Ap	RT (18-25°C)	See below	Testing Laboratory to purchase
LIVE/DEAD™ Fixable Aqua Dead Cell Stain	Thermo Fisher	L34957	-20°C	See below	NIST

Pacific Blue™ Annexin V	Thermo Fisher	A35136	-2-8 °C	Ready to use	NIST
Trucount™ Tubes (# beads/tube: 46800)	BD Biosciences	340334	2-8 °C	Ready to use	NIST
Type II water, or better	N/Ap	N/Ap	Per Testing Laboratory's SOP	N/Ap	Testing Laboratory to purchase

3.2. Supplies

- Staining Tubes: 5 mL polystyrene round-bottom tubes w/o snap caps
- Pipet tips (P10, P20, P100, P200, P1000)
- 15 mL and 50 mL polypropylene conical tubes with screw caps
- 96-well plates, per your institution's SOP (for testing labs that stain in plates, only)
- Sterile serological pipettes, as required (e.g., 2 mL, 5 mL, 10 mL, 25 mL)

3.3. Equipment

- Pipettors P10, P20, P100, P200, P1000
- Pipette-aid
- Water Bath
- Cell counting equipment
- Centrifuge with swinging bucket rotor
- Biosafety Cabinet, class II, appropriate for BSL-2 per your institution's policies
- Your institution's cytometer(s), as described in Study Plan WG2-01, Appendix A

3.4. Reagent Preparation

70% Ethanol

Prepare per your institution's SOP.

RPMI-1640 Complete Cell Culture Media

Prepare a solution of RPMI-1640 containing a final volume of 10% FBS and 1% Penicillin-Streptomycin per your institution's SOP.

Staining Buffer

Prepare a solution of 1X PBS, 3.5 mM $CaCl_2$, containing a final volume of 2% FBS, pH 7.4, per your institution's SOP.

LIVE/DEAD™ Fixable Aqua Dead Cell Stain

This kit contains:

- Component A – Aqua dye
- Component B - Dimethylsulfoxide (DMSO), anhydrous

Bring 1 vial of fluorescent reactive dye (Component A) and 1 vial of anhydrous DMSO (Component B) of Aqua Live/Dead Staining kit to room temperature before removing the caps.

Add 50 µL of DMSO to the vial of reactive dye. Mix well and visually confirm that all of the dye has dissolved. Keep the dye solution in dark at RT and use it within 2 hours of reconstitution.

FMO Cocktails

For Test Samples label nine polypropylene tubes FMO-TS-1, FMO-TS-2.... FMO-TS-9 as indicated in Table 5

NOTE 5: Note that FMO controls will NOT be used for the QC material.

The mAb have been titrated and bottled so that 5 µL of each mAb is used for optimal staining.

Calculate the total number of samples to be processed per analytical run (n).

Prepare enough cocktail for n+1 samples.

> ***Example***
>
> Number of samples = 3
> n+1=4
> Add 20 µL (i.e., 4 × 5 µL) of each mAb EXCEPT for one as indicated in Table 5 to the appropriate tube.

Keep at 2-8 °C protected from light. Use within 60 minutes of preparation.

Immunophenotyping Tube Cocktail

For the Test Sample, label a polypropylene tube FC-TS-# as indicated in Table 6.

For the QC material, label a polypropylene tube FC-QC as indicated in Table 8.

The mAbs have been titrated and bottled so that 5 µL of each mAb is used for optimal staining.

Calculate the total number of samples to be processed per analytical run (n).

Prepare enough cocktail for n+1 samples.

Example
Number of samples = 3
n+1=4
Add 20 µL (i.e., 4 × 5 µL) of each mAb to a polypropylene tube labeled FC

Keep at 2-8 °C protected from light. Use within 60 minutes of preparation.

Counting Tube Cocktail

For the Test Sample, label a polypropylene tube TS-TC-# as indicated in Table 7.

For the QC material, label a polypropylene tube QC-TC as indicated in Table 9. The mAbs have been titrated and bottled so that 5 µL of each mAb is used for optimal staining.

Calculate the total number of samples to be processed per analytical run (n).

Prepare enough cocktail for n+1 samples.

Example
Number of samples = 3
n+1=4
Add 20 µL (i.e., 4 × 5 µL) of each mAb to a polypropylene tube labeled FC

Keep at 2-8 °C protected from light. Use within 60 minutes of preparation.

Table 5. Test Sample FMO Staining Map. NOTE: Cells are prelabeled with Aqua. EXCEPT for FMO-TS-1 which will use PBMC not prelabeled with Aqua. No additional Aqua will be added to the FMO Staining Cocktails.

Tube #	Material Code	Aqua	CD3	CD4	CD8	CD14	CD16	CD56	CD19	CD45	Annexin
**FMO-TS-1**	cryoPBMC-AqLD-fmo	-	5 µL	5 µL	5 µL	5 µL	5 µL	5 µL	5 µL	5 µL	5 µL
**FMO-TS-2***	cryoPBMC-FITC-fmo	1 µL	-	5 µL	5 µL	5 µL	5 µL	5 µL	5 µL	5 µL	5 µL
**FMO-TS-3***	cryoPBMC-PE-Cy7-fmo	1 µL	5 µL	-	5 µL	5 µL	5 µL	5 µL	5 µL	5 µL	5 µL
**FMO-TS-4***	cryoPBMC-APC-Cy7-fmo	1 µL	5 µL	5 µL	-	5 µL	5 µL	5 µL	5 µL	5 µL	5 µL
**FMO-TS-5***	cryoPBMC-APC-CD14-fmo	1 µL	5 µL	5 µL	5 µL	-	5 µL	5 µL	5 µL	5 µL	5 µL
**FMO-TS-6***	cryoPBMC-PE-fmo	1 µL	5 µL	5 µL	5 µL	5 µL	-	-	5 µL	5 µL	5 µL

**FMO-TS-7***	cryoPBMC-APC-CD19-fmo	1 µL	5 µL	5 µL	5 µL	5 µL	5 µL	5 µL	-	5 µL	5 µL
**FMO-TS-8***	cryoPBMC-PerCP-Cy5.5-fmo	1 µL	5 µL	5 µL	5 µL	5 µL	5 µL	5 µL	5 µL	-	5 µL
**FMO-TS-9***	cryoPBMC-AxPB-fmo	1 µL	5 µL	5 µL	5 µL	5 µL	5 µL	5 µL	5 µL	5 µL	-

Table 6. Test Sample Immunophenotyping Tube Staining Map. Cells are prelabeled with Aqua. No additional Aqua will be added to the FC Staining Cocktail. Number sign is Test Sample number. Three replicates are prepared for each donor.

Tube #	Material Code	CD3	CD4	CD8	CD14	CD16	CD56	CD19	CD45	Annexin
**FC-TS-#**	panel1-cryoPBMC-#	5 µL	5 µL	5 µL	5 µL	5 µL	5 µL	5 µL	5 µL	5 µL

Table 7. Test Sample Counting Tube Staining Map. Cells are prelabeled with Aqua and added to a Trucount tube. No additional Aqua will be added to the Cell Counting Staining Cocktail. Number sign is Test Sample number. Two replicates are prepared for each donor.

Tube #	Material Code	CD45	Annexin
**TS-TC-#**	AqLD-TruCount-CD45-AxPB-cryoPBMC-#	5 µL	5 µL

Table 8. QC Immunophenotyping Tube Staining Map. No Aqua or Annexin will be used for the QC FC synthetic cell Trucytes Staining Cocktail. Number sign is QC material number. Three replicates are prepared, one replicate per vial of QC material.

Tube #	Material Code	CD3	CD4	CD8	CD14	CD16	CD56	CD19	CD45
**FC-QC**	panel1-TruCytes-#	5 µL	5 µL	5 µL	5 µL	5 µL	5 µL	5 µL	7.5 µL

Table 9. QC Counting Tube Staining Map. No Aqua or Annexin will be used for the QC FC synthetic cell TruCytes Staining Cocktail. Two replicates are prepared, one replicate per vial of QC material.

Tube #	Material Code	CD45
**QC-TC**	TruCount-CD45-TruCytes-#	7.5 µL

4. Procedure

4.1. Instrument Setup

Perform the daily instrument calibration per your institution's SOP.

4.2. Worklist Creation

Create a worklist containing sample tubes with appropriate local file names consistent with 'Materials' and 'Material Code' in the 'Test and QC Samples' of the WG2 Reporting Spreadsheet. Staining samples include FMO controls and test and synthetic cell samples in the 'Test and QC Samples' sheet.

4.3. Test Sample Preparation

4.3.1. PBMC Thawing

Proper aseptic technique should be used when handling and manipulating cells. Perform in biosafety cabinet. Cells are to be thawed as rapidly as possible. A high concentration of DMSO can be toxic to the cells at room temperature or at 37°C.

- Warm RPMI-1640 Complete Cell Culture Media to room temperature (18-25°C).
- Removed vials from liquid nitrogen storage and keep on dry ice until ready for thawing.
- Check that the vial is tightly capped and place in a 37°C water bath.
- After 60 seconds at 37°C, remove the vial from the water bath every 15-20 seconds and gently invert 3-4 times to check the level of thawing.
- When the vial contents are 50-75% liquid, remove from the water bath and continue to gently invert the vial until the entire contents are liquid.
- Place the vial on wet ice and transport to a biosafety cabinet.
- Wipe the outside of the vial with an alcohol wipe and gently invert 5 times to mix the cells.
- Check to see if there is liquid caught up in the cap. If so, gently tap the upright vial on a benchtop to force the liquid into the vial.
- Transfer quickly the vial contents to a 15 mL tube at RT (18-25°C).
- Add 1 mL of media to rinse the vial and transfer any remaining cells to the 15 mL tube.
- Add 10 mL media dropwise (about 1 mL per second) to the cells. Securely cap the tube and gently invert 3-5 times after adding all the media.

– Centrifuge the tube at 200 × *g* for 10 minutes at RT (18-25 C).

– Aspirate the supernatant without disturbing the pellet and resuspend the cells in 10 mL media, e.g., 1 mL first to resuspend cells followed by addition of 9 mL media.

– Remove a small aliquot of cell suspension (≤ 50 µL) to perform cell counting and viability assessment per your institution's SOP, for example using a hemocytometer and trypan blue or automated cell counting/viability instrument.

– Centrifuge at 350 × *g* for 5 minutes at RT (18-25°C) and resuspend in an appropriate volume of PBS to achieve cell concentration of 1 x 10^6/mL.

– **NOTE 6:** Aqua Dead Cell must not be added to FMO-TS-1. Reserve 0.5x10^6 cells for FMO-TS-1 before the last step of centrifugation and cell suspension in PBS.

– **NOTE 7:** If cells are to be used for compensation controls as described in WG2 SPO-2, reserve appropriate number of cells prior to the last step, centrifuge and resuspend cells in the staining buffer instead of PBS.

– **NOTE 8: Participants need to re-assess the cell number/volume for Section 4.3.2.**

4.3.2. Test Sample Aqua Dead Cell Stain

– Add 10 mL of cells (1 x 10^6/mL) from Section 4.3.1 to a 15 mL polystyrene tube.

– Add 10 µL reconstituted Aqua dye (see Section 4). Incubate for 30 minutes at RT, protected from light.

– Centrifuge at 350 × *g* for 5 minutes. Aspirate the supernatant. Resuspend the cell pellet. Add 3 mL Staining Buffer.

– Centrifuge at 350 × *g* for 5 minutes.

– Aspirate the supernatant. Resuspend the cell pellet. Add 1 mL Staining Buffer.

4.3.3. Test Sample FMO Staining (exp ID=e1)

– For ONE of the Test Samples, label 9 tubes with the Test Sample ID and FMO-TS-1, FMO-TS-2.... FMO-TS-9 (see Table 5)

– **For tube FMO-TS-1 add 100 µL of thawed PBMC which were not labeled with Aqua (see Section 4.3.1). Note 9**: The rest of thawed PBMC in PBS from Section 4.3.2 shall be centrifuged at 350 × *g* for 5 minutes at RT (18-25°C) and

resuspended in the staining buffer to achieve cell concentration of 1 x 10^6/mL for other usages such as FMO-TS-1 and cryoPBMC compensation tubes in SOP-02.

– For FMO-TS-2, FMO-TS-3…. FMO-TS-9 tubes, add 50 µL cells (prepared in Section 4.3.2, i.e. Aqua labeled) and 50 µL staining buffer to each tube.

– Add required volumes of antibodies provided in Table 5 or 35-45 µL of the appropriate FMO-TS cocktail cocktail to each tube.

– Mix well by gentle vortex.

– Incubate at RT (18-25°C) protected from light for 30 minutes.

– Add 2 mL of Staining Buffer.

– Centrifuge at 350 × g for 5 minutes at RT (18-25°C). Aspirate the supernatant. Resuspend the cell pellet. Add 2 mL Staining Buffer.

– Centrifuge 350 × g for 5 minutes at RT (18-25°C). Aspirate the supernatant. Resuspend the cell pellet.

– Add 500 µL of Staining Buffer.

– Hold at 2-8 °C, protected from light if not acquired on the cytometer immediately.

– Acquire on the instrument with 60 minutes of preparation.

– Vortex the thoroughly (at low speed) immediately prior to acquisition.

4.3.4. Test Sample Immunophenotyping Tube Staining (exp ID=e1)

NOTE 10: The Test Sample Immunophenotyping Tube will be stained in triplicate.

- For EACH Test Sample, label 3 tubes with the Test Sample ID and FC-TS-1, FC-TS-2, FC-TS-3 (see Table 6).
- For FC-TS-1, FC-TS-2, FC-TS-3 tubes, add 100 µL cells (prepared in Section 4.3.2, i.e. Aqua labeled) to each tube.
- Add required volumes of antibodies provided in Table 6 or 45 µL of the appropriate FC-TS cocktail to each tube.
- Mix well.
- Incubate at RT (18-25°C) protected from light for 30 minutes.
- Add 2 mL of Staining Buffer.

- Centrifuge at 350 × *g* for 5 minutes at RT (18-25°C). Aspirate the supernatant. Resuspend the cell pellet. Add 2 mL Staining Buffer.
- Centrifuge 350 × *g* for 5 minutes at RT (18-25°C). Aspirate the supernatant. Resuspend the cell pellet.
- Add 500 µL of Staining Buffer.
- Hold at 2-8 °C, protected from light if not acquired on the cytometer immediately.
- Acquire on the instrument with 60 minutes of preparation.
- Vortex the thoroughly (at low speed) immediately prior to acquisition.

4.3.5. Test Sample Counting Tube Staining (exp ID=e2)

NOTE 11: The Test Sample Counting Tube will be stained in **duplicate**.

– For each Test Sample, label 1 Trucount™ tube with the Test Sample ID and TS-TC (Table 7).

– Add 100 µL cells (prepared in Section 4.3.2) to the appropriate Trucount™ tube.

– Add 5 µL of CD45 PerCP-Cy5.5 and 5 µL of Annexin V Pacific Blue (or 10 µL counting tube cocktail).

– Vortex gently to mix.

– Incubate at RT protected from light for 30 minutes.

– Add 500 µL of Staining Buffer. Cap the tube and vortex gently to mix.

– Hold at 2-8 °C, protected from light if not acquired on the cytometer immediately.

– Acquire on the instrument with 60 minutes of preparation.

– Vortex the thoroughly (at low speed) immediately prior to acquisition.

4.4. QC/Synthetic cell TruCytes Preparation

4.4.1. TruCytes TBMNK Synthetic Cell Reconstitution

– Add 105 µL of Type II sterile water to the vial. Let sit at RT (18-25°C) for 15 minutes. Mix by gentle pipetting just prior to use.

– After reconstitution, store at 2-8°C for up to seven days.

4.4.2. QC Synthetic Cell Counting Tube Staining (exp ID=e3)

NOTE 12: The QC material Counting Tube will be stained in singlicate. PBS buffer is preferred for handling QC material instead of the staining buffer that might cause aggregation around QC material. Additionally, the detection threshold could be lowered to ensure the inclusion of all lymphocytes due to the low refractive index of the lymphocytes.

- Label 1 Trucount™ tube as QC-TC (Table 9).
- Add 100 µL synthetic cells (prepared in Section 4.4.1) to the Trucount™ tube.
- Add 7.5 µL of CD45 PerCP-Cy5.5.
- Vortex gently to mix.
- Incubate at RT protected from light for 30 minutes.
- Add 500 µL of PBS. Cap the tube and vortex gently to mix.
- Hold at 2-8 °C, protected from light if not acquired on the cytometer immediately.
- Acquire on the instrument within 60 minutes of preparation.
- Vortex the thoroughly (at low speed) immediately prior to acquisition.

4.4.3. QC Synthetic Cell Immunophenotyping Tube Staining (exp ID=e4)

NOTE 13: The QC material Immunophenotyping Tube will be stained in singlicate. PBS buffer is preferred for handling QC material instead of the staining buffer that might cause aggregation around QC material. Additionally, the detection threshold could be lowered to ensure the inclusion of all lymphocytes due to the low refractive index of the lymphocytes.

- Label a single tube as QC-FC (Table 8). Note that the QC synthetic cells from each vial will be stained in singlicate.
- Add 100 µL synthetic cells (prepared in Sections 4.4.1).
- Add required volumes of antibodies provided in Table 8.
- Mix well.
- Incubate at RT (18-25°C) protected from light for 30 minutes.
- Add 2 mL of PBS.
- Centrifuge at 500 × g for 8 minutes at RT (18-25°C). Aspirate the supernatant. Resuspend the cell pellet. Add 2 mL PBS.

- Centrifuge 500 × *g* for 8 minutes at RT (18-25°C). Aspirate the supernatant. Resuspend the cell pellet.
- Add 500 µL of PBS.
- Hold at 2-8 °C, protected from light if not acquired on the cytometer immediately.
- Acquire on the instrument with 60 minutes of preparation.
- Vortex the thoroughly (at low speed) immediately prior to acquisition.

4.5. Acquisition

- Create an acquisition template for the TBMNK Test Samples and a second acquisition template for the TBMNK QC. Note: Plots from the gating hierarchy provided in SOP-04 can serve as acquisition templates.
- Use the gain settings for all fluorescence detectors finalized in the SOP-01. The gain settings for both FSC and SSC can be adjusted for different sample types if needed.
- The threshold should be set on the PerCP-Cy5.5 channel so that most of the debris is excluded from the CD45+ events.
- Acquire samples WITHOUT compensation and with a minimum of 50,000 events in the CD45+ gate. Note that compensation will be applied post-acquisition.
- **Note 14**: For acquisition of QC material, carefully adjust gains for FSC and SSC to minimize the loss of synthetic cells due to the low refractive index of the synthetic lymphocyte population (See examples provided in Appendix C and D in SOP-04).

4.6. Local Filename Entry on the WG2 Test and QC Samples Reporting Spreadsheet

- Open the WG2 Reporting Workbook and select the “Test and QC Samples” Tab. Verify that the Study ID, Site ID, Instrument ID and Protocol ID at the top of the Spreadsheet describe the current execution of the protocol. See Figure 2 for example. One Workbook is required for each instrument.
- For each FCS file collected (29 total files) find the corresponding material code row using the terms in “Column D” and description in “Column C”. See Figure 2.
- Identify the associated “Get Local FCS Filename” button in Column H to select the local FCS file location for Column G (directory is stored in hidden Column I). See Figure 2.

- Press the button and use the file dialog box to identify the local FCS filename corresponding to the files collected in Section 4.5.

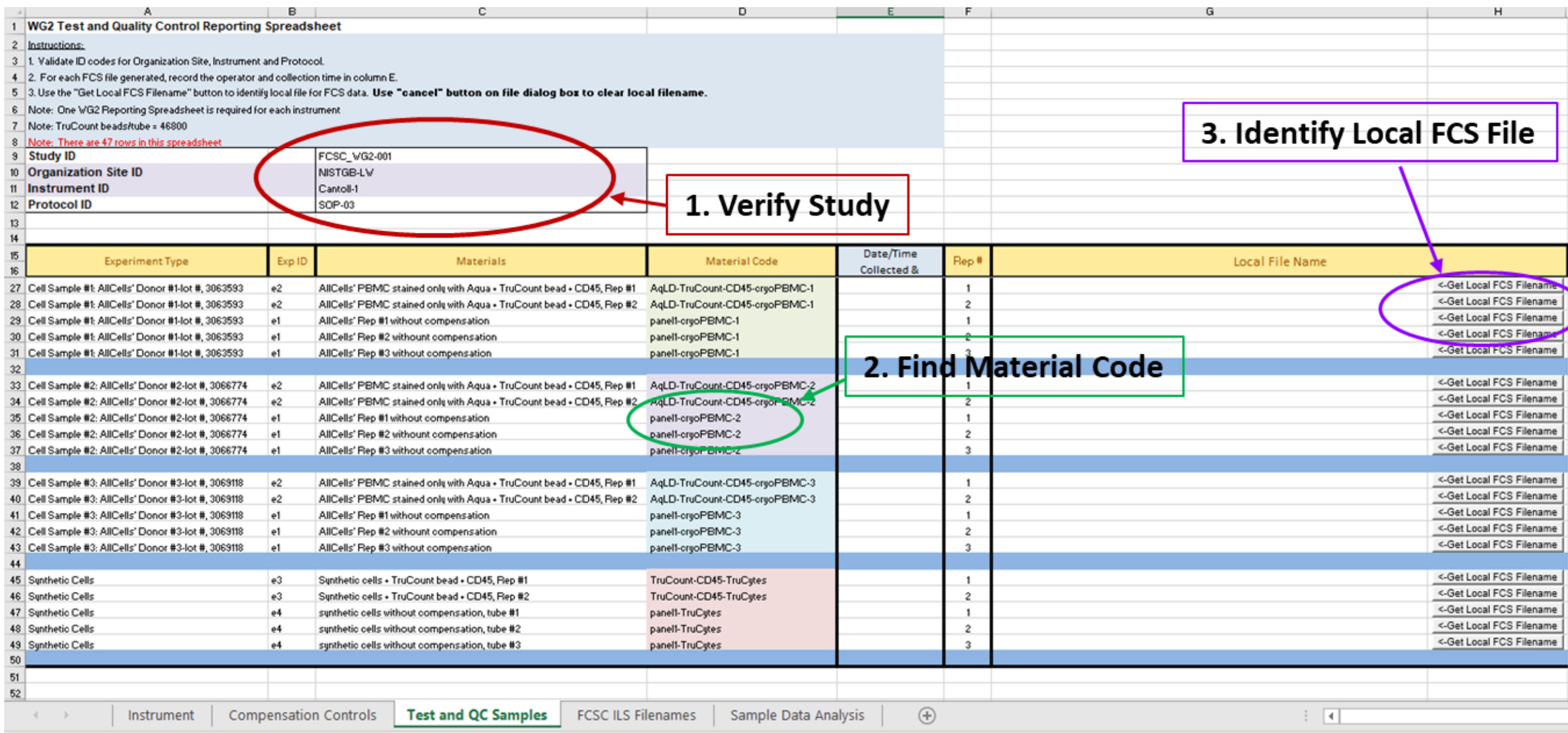

Figure 2. Test and QC Samples Reporting Spreadsheet.

4.7. Generate FCSC Files with Standardized File Names

- Once the files have been collected during SOP-01, SOP-02 and SOP-03 have been recorded in the WG2 reporting spreadsheets (Instrument, compensation control, Test and QC Samples Tabs), select the "FCSC ILS Filenames" tab. This is shown in Figure 3.
- Press the "1. Clear and Update List" Button. This will clear the spreadsheet and transfer the local FCS filenames and the relevant metadata from each of the data collection worksheets to the FCSC ILS Filenames spreadsheet.
- Press the "2. Validate List" Button. This checks that the data transferred will result in the generation of unique ILS filenames for each FCS file. Rows will be highlighted if non-unique FCSC ILS filename or missing metadata are detected. Cells will also be highlighted if they have space or underline characters. Metadata in the highlighted cells will need to be manually checked and corrected in the spreadsheets.

  NOTE 15: If other FCS files from protocol or technical replicates are generated, additional instructions are required for file inclusion. See study organizers.

- Once data has passed validation, press the "3. Make ILS filename" button. This will concatenate each of the metadata terms and generate a standardized FCSC

ILS filename for each row. The filename will be observed in column N "FCSC ILS filename". Each of these names will be unique and in the FCSC ILS filenaming format.

- Press the "4. Generate ILS Directory" button to generate a directory that contains copies of the WG2 FCS files renamed with the standardized ILS filename. Pressing this button brings up a filesave dialog box populated with a predefined directory generated from the study metadata. Identify where the ILS directory should be saved and press "OK".
- A directory window will automatically appear with the new FCS data with the standardized filenames. Verify the correct FCS files with the new filenames were written to the directory.
- ***NOTE 16:*** This directory and included files will be used for bulk raw data analysis in WG3.

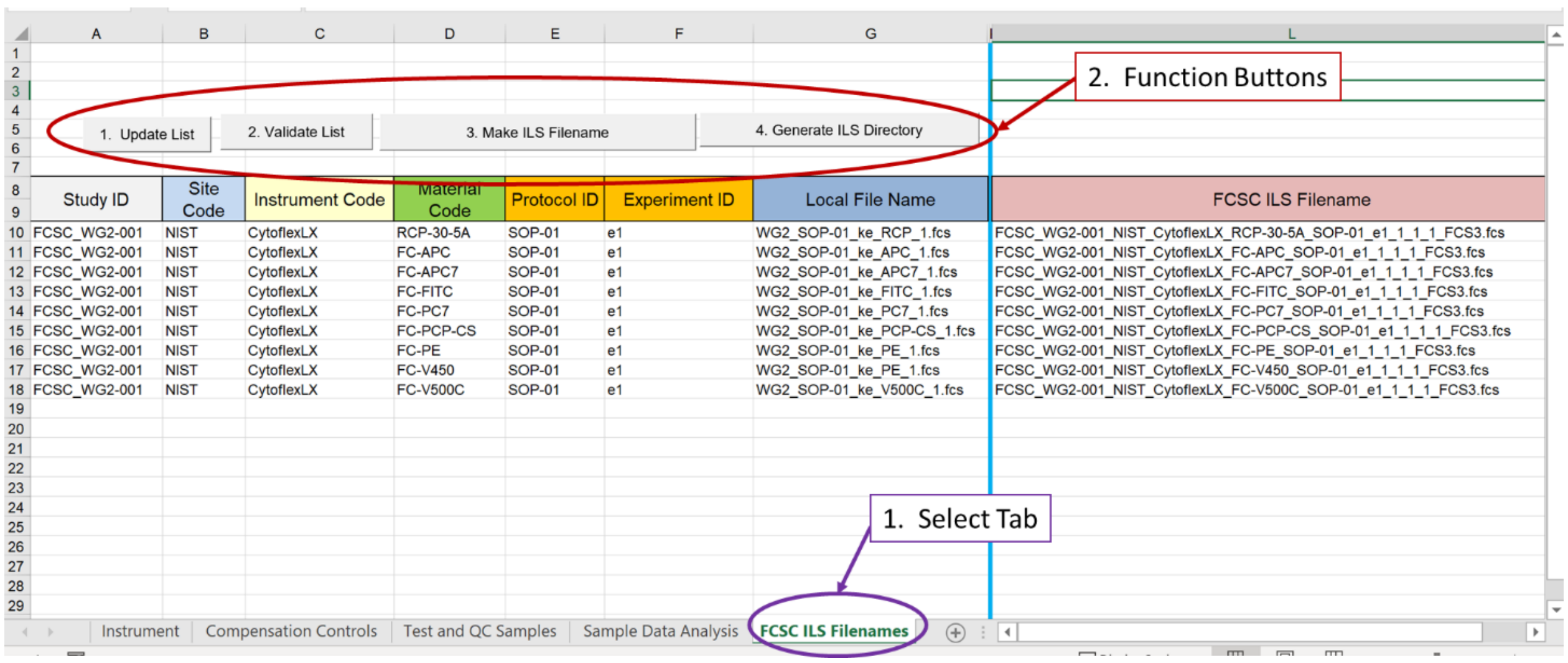

Figure 3. FCSC ILS Filename generation spreadsheet

5. Attachments

- AllCells Thawing Protocol
- Slingshot TDS for TruCytes TBMNK

For NIST Consortium member use only

WG2 SOP-04 Data Analysis

Overview

This Standard Operating Procedure (SOP) describes the data analysis of the data generated from both SOP-1 and SOP-03 and to be used for the Working Group 2 (WG2) Inter-Laboratory Study (ILS). The data analysis includes the test samples and QC material acquired during SOP-03 and to which the 3 compensation matrices generated in SOP-02 will be applied. In addition, the data analysis will be performed with data obtained from SOP-1 to calibrate the fluorescence response, in units of Spe and ERF, to estimate the background (B, in units of Spe and ERF) and the detection efficiency (Q, units of Spe/ERF), which can be used to estimate the limit of detection and to predict instrument performance in resolving particles of different brightness, and to obtain the expression levels of CD markers in the unit of ERF.

Each generated .fcs file from SOP-03 will be analyzed with each of the 3 compensation matrices according to the workflow summarized in Figure 1.

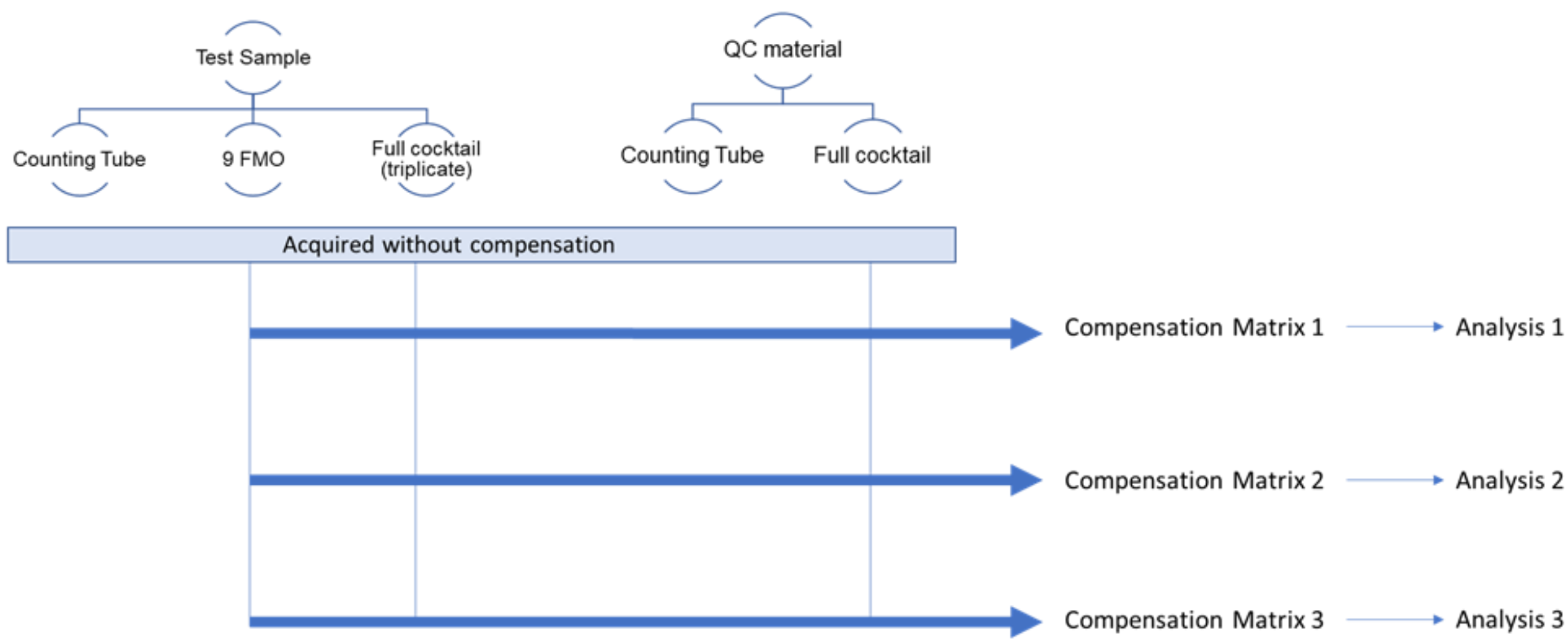

Figure 1. Data Analysis workflow of the WG2 ILS.

Data analysis may be performed on any analysis software but will follow gating plots and guidelines as described in this SOP.

For the analysis of instrument sensitivity, background, ERF calibration, and CD marker expression, etc., an automated WG2 Cytometer Sensitivity and ERF Calibration spreadsheet provided for the study will be used.

Equipment

- Flow Cytometry Analysis Software (use software and version of your institution or acquisition software provided with the cytometer
- WG2 Cytometer Sensitivity and ERF Calibration spreadsheet
- Computer

Procedure

1.1. Analysis template for Trucount™ counting tube for Test Sample

Create Single Analysis Template that will be used across all samples and analyses according to the following guidelines.

Plot 1

A bivariate dot plot, Time (X-axis) versus CD45 PerCP-Cy5.5-A (Y-axis), of all collected events. This plot is used for acquisition quality monitoring. Note that if a fluctuation is observed, e.g., stark dip or gap in the time profile, participant should consider to re-start sample acquisition or avoid the use of data collected during the fluctuation in the data analysis.

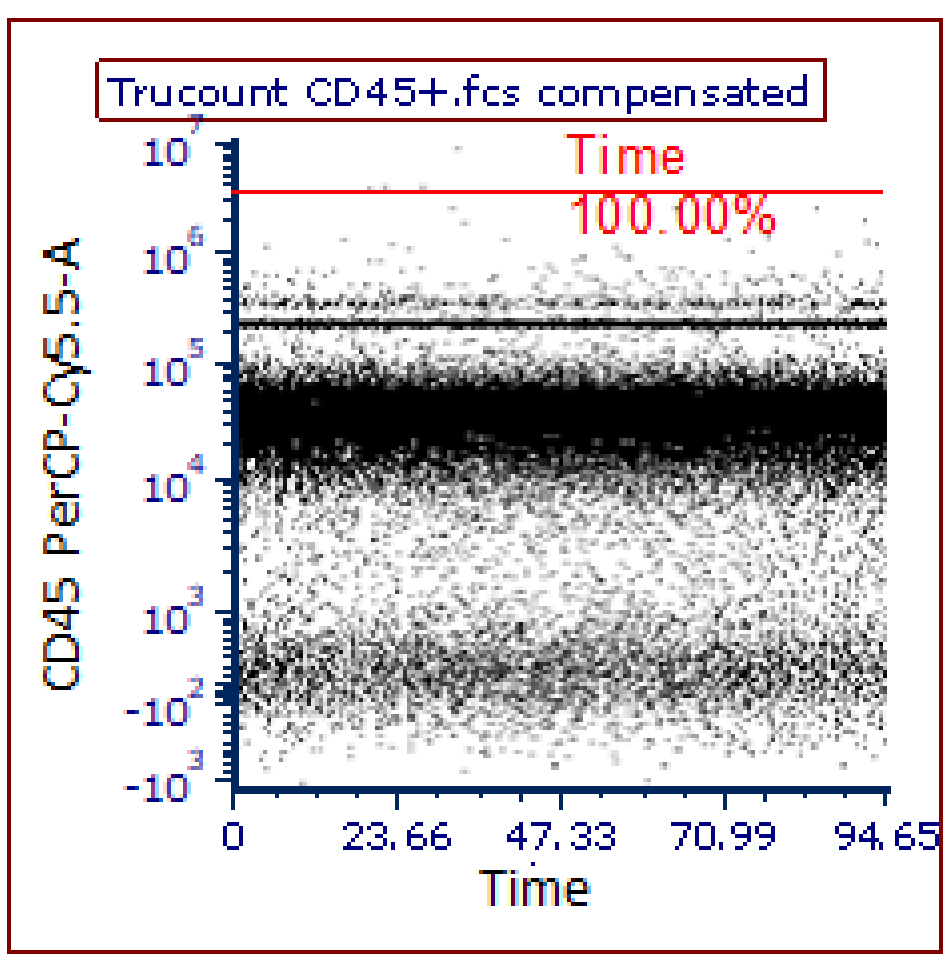

Plot 2

A bivariate dot plot, APC-A (X-axis) versus SSC-A (Y-axis), of events in 'Time' gate. This plot is used to draw a region around the "Beads" and the cellular events "Not beads". Ensure all beads are taken in the gate. Note specific APC fluorescence is not detected because all cellular events are APC negative. Beads autofluorescence is used to detect and gate them.

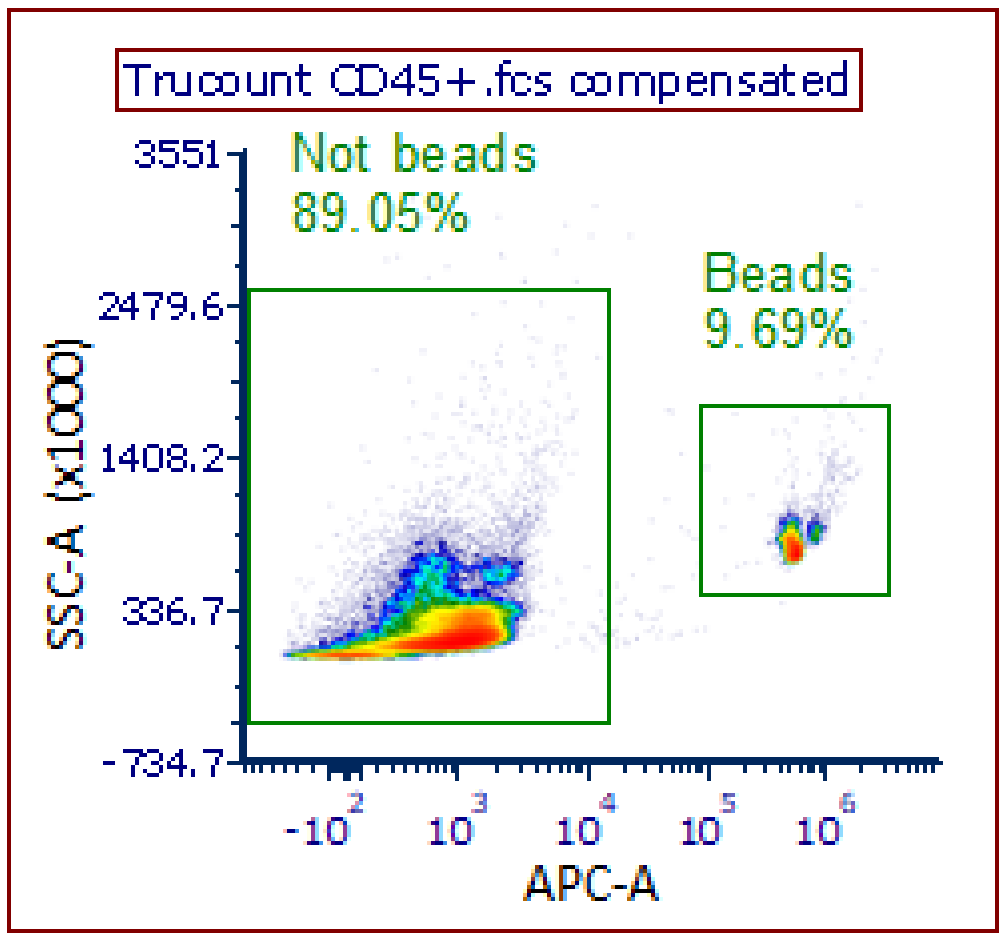

Plot 3

A bivariate dot plot, SSC-A (X-axis) versus SSC-H (Y-axis), of "Not beads". This plot is used to draw a region around single cells to exclude side scatter doublets, named "SSC Singlets". Note that singlet events are identified and gated based on Side Scatter (SSC) signal processing of Area (SSC-A) and Height (SSC-H). Doublets will have double the area values of single cells while the height is roughly the same. Therefore, differences between height and area can be used to identify aggregates.

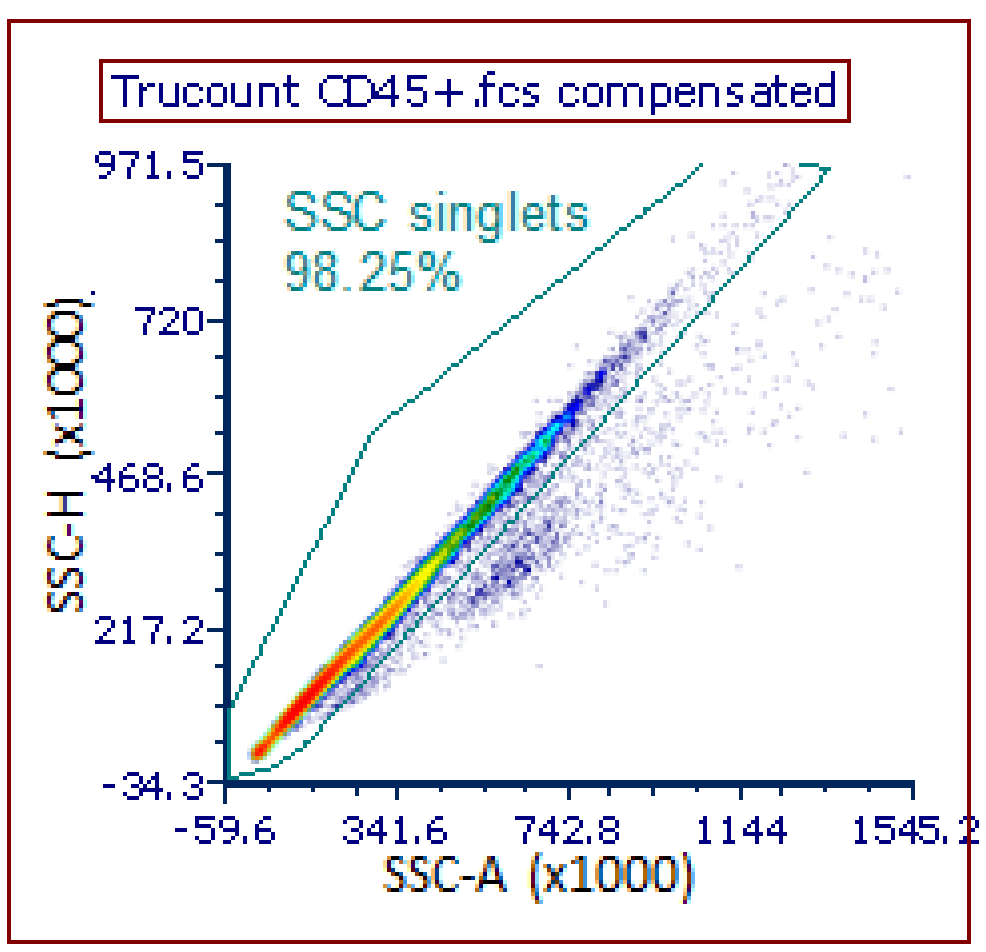

Plot 4

A bivariate dot plot, FSC-A (X-axis) versus FSC-H (Y-axis), of events in 'SSC singlets' gate. This plot is used to draw a region around single cells to exclude forward scatter doublets, named "Singlets". A similar principle of signal processing applies as in Plot 3.

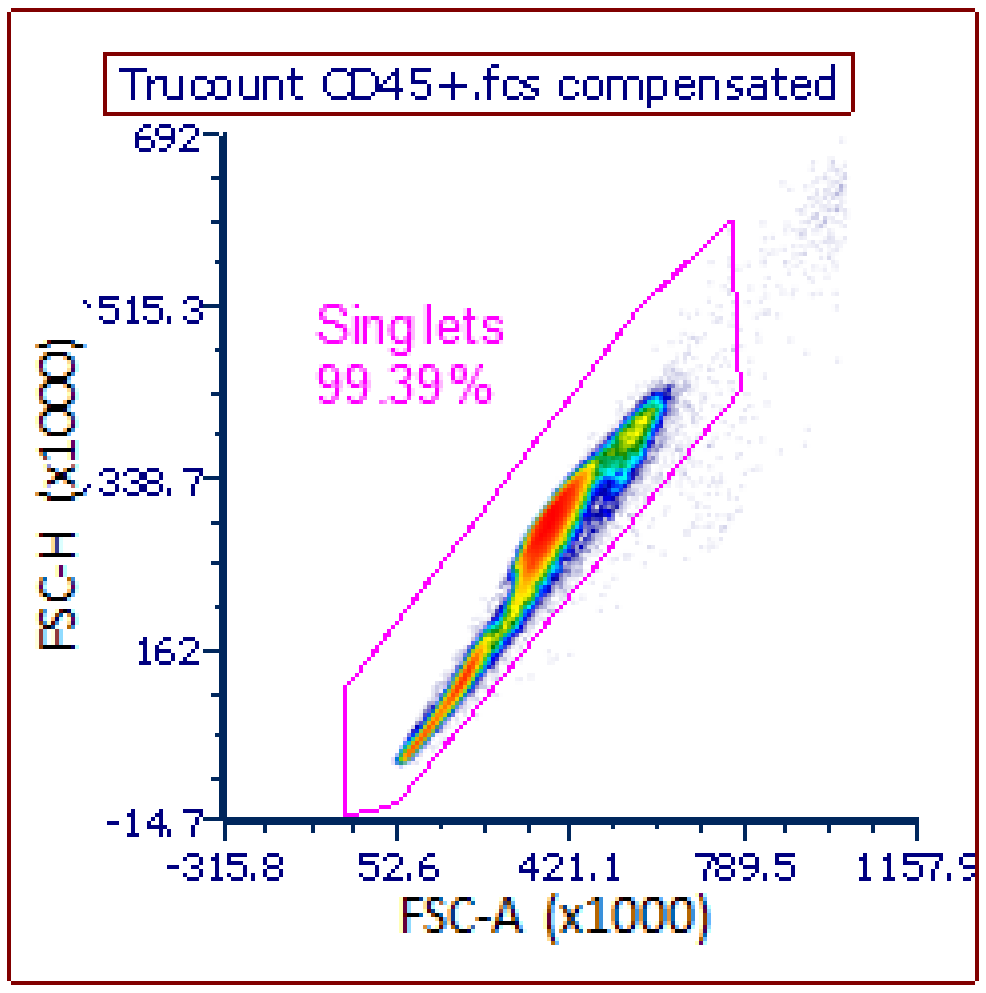

Plot 5

A bivariate dot plot, Annexin V Pacific-Blue-A (X-axis) versus Live/Dead Aqua-A (Y-axis), of events in 'Singlets' gate. This plot is used to draw a region around cells to exclude dead and apoptotic cells, named "Annexin-Aqua- cells".

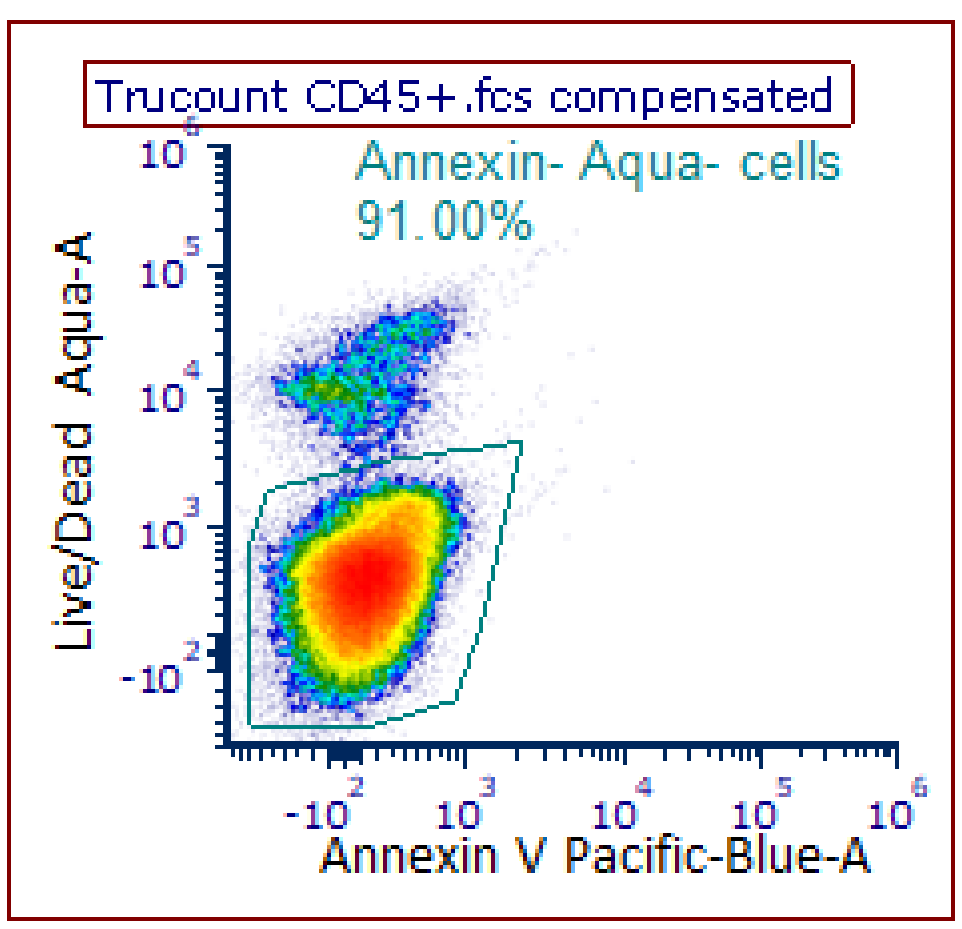

Plot 6

A bivariate dot plot, CD45 PerCP-Cy5.5-A (X-axis) versus SSC-A (Y-axis), of events in 'Annexin-Aqua-cells' gate. This plot is used to draw a region around the "CD45+ mononuclear cells". The events in this gate will be used for the calculation of total cell counts.

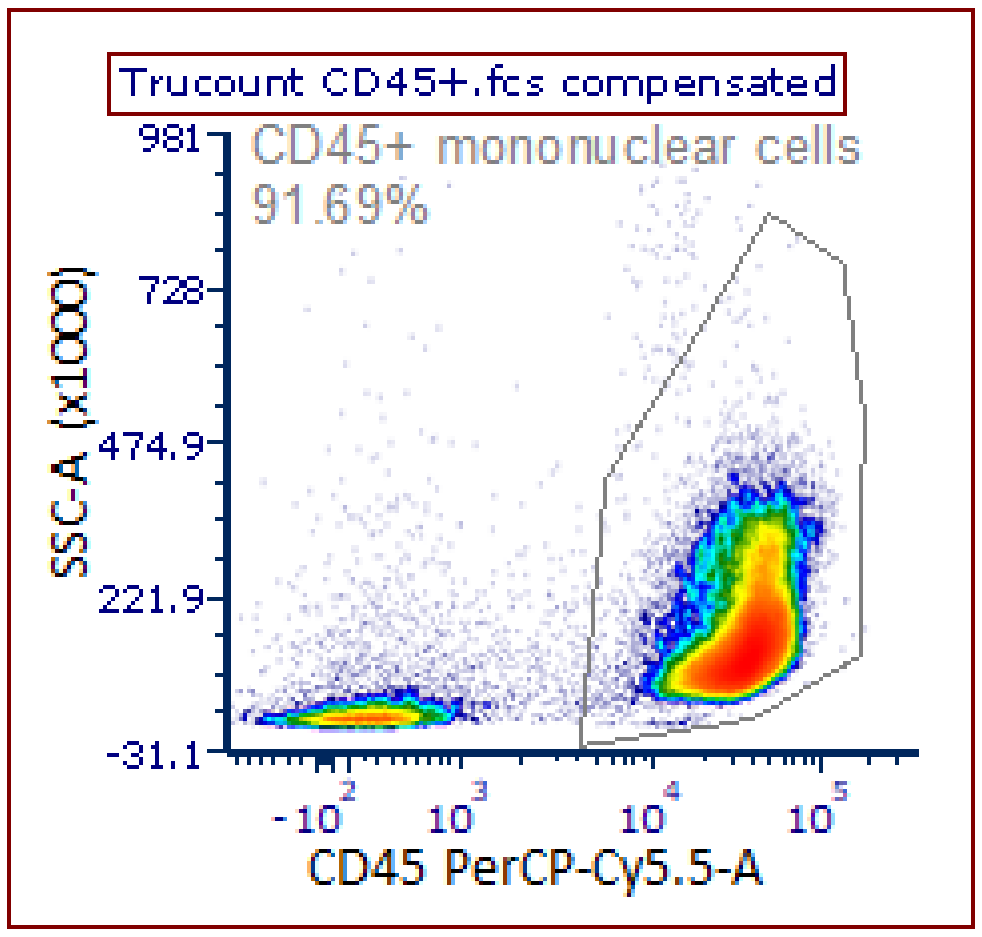

Population Hierarchy

Population hierarchy for the counting tube is provided below.

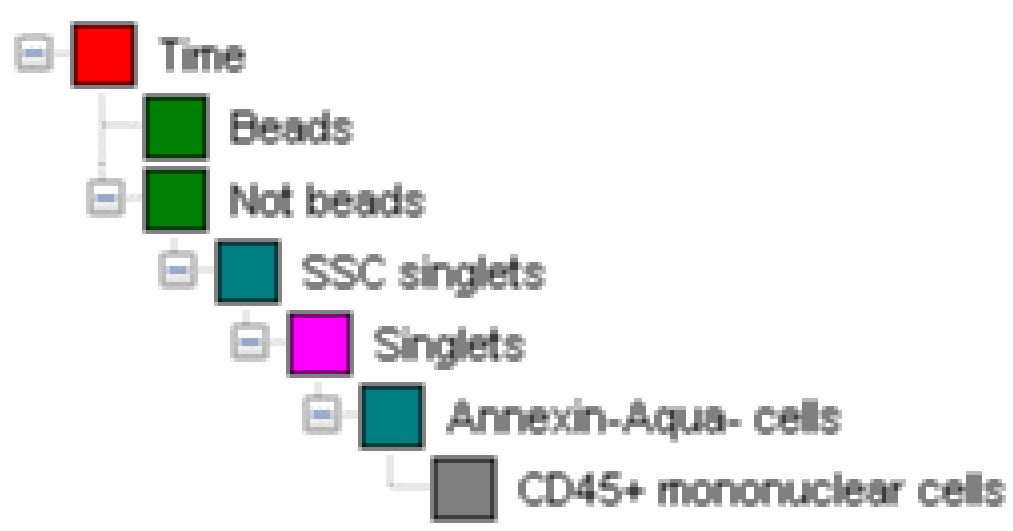

1.2. Analysis template for TBMNK panel for Test Sample

Create Single Analysis Template that will be used across all samples and analyses according to the following guidelines.

Plot 1

A bivariate dot plot, time (X-axis) versus CD3 FITC-A (Y-axis), of all collected events. This plot is used for acquisition quality monitoring.

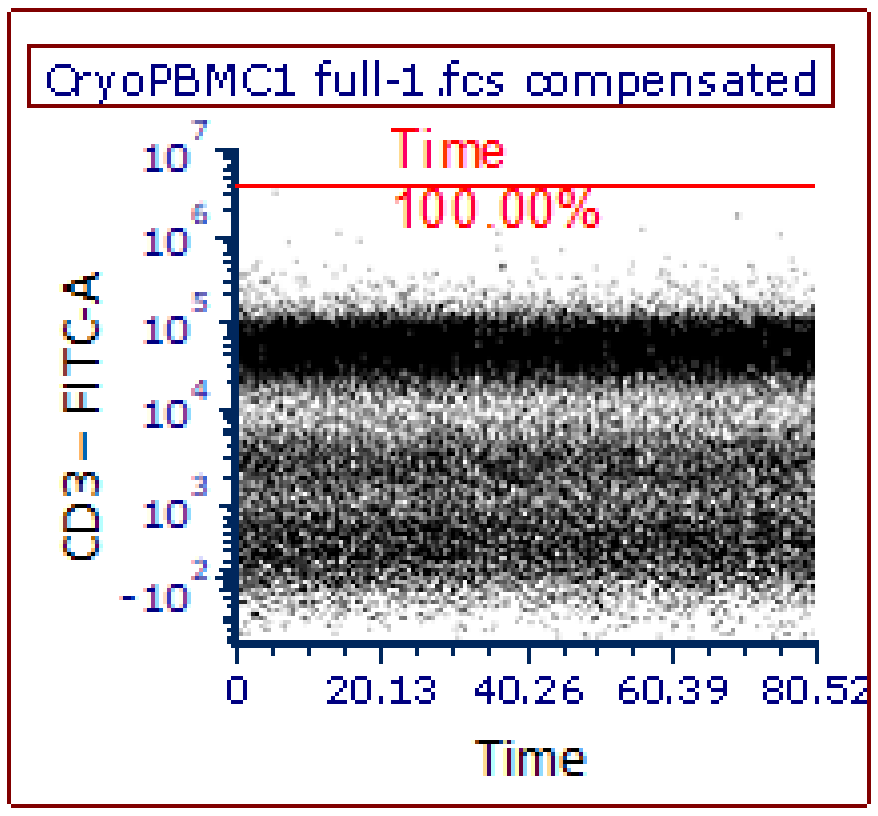

Plot 2

A bivariate dot plot, SSC-A (X-axis) versus SSC-H (Y-axis), of events in 'Time' gate. This plot is used to draw a region around single cells to exclude side scatter doublets, named "SSC Singlets". A similar consideration is applied as above in Plot 3 of Section 1.1.

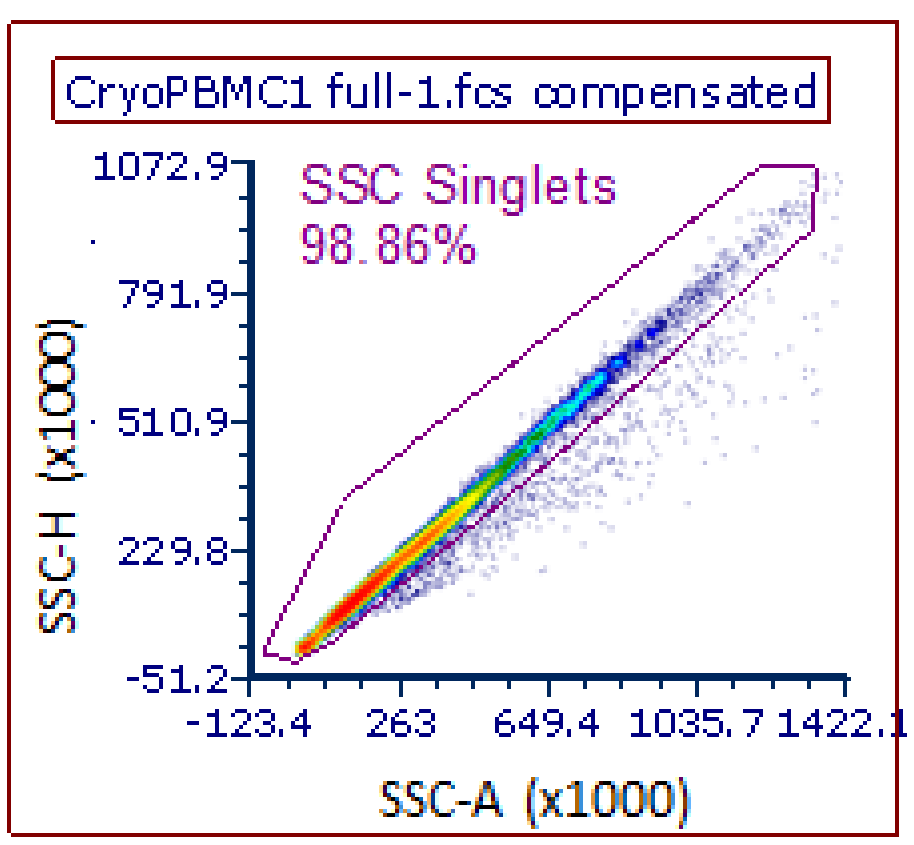

Plot 3

A bivariate dot plot, FSC-A (X-axis) versus FSC-H (Y-axis), of events in ‘SSC singlets’ gate. This plot is used to draw a region around single cells to exclude forward scatter doublets, named “Singlets”. A similar consideration is applied as above in Plot 4 of Section 1.1.

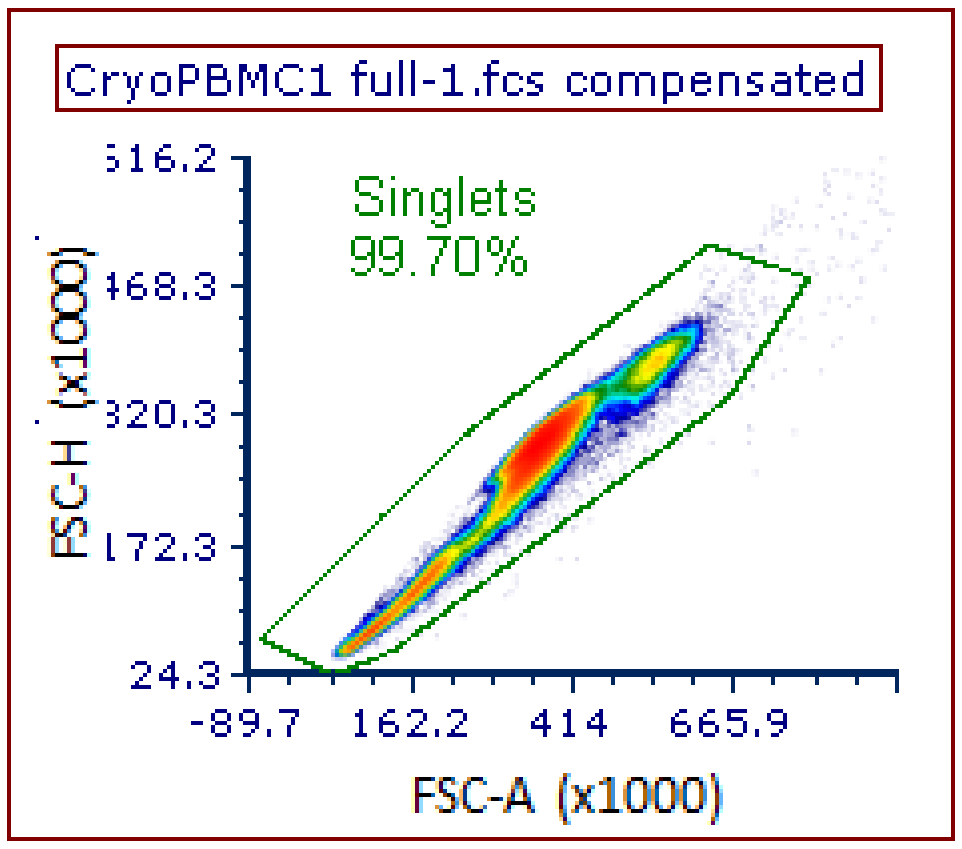

Plot 4

A bivariate dot plot, Annexin V Pacific Blue-A (X-axis) versus Live/Dead Aqua-A (Y-axis), of ‘Singlets’. This plot is used to draw a region around single cells to exclude dead and apoptotic cells that are positive for cellular amines and Annexin V, named “Annexin-Aqua- cells”.

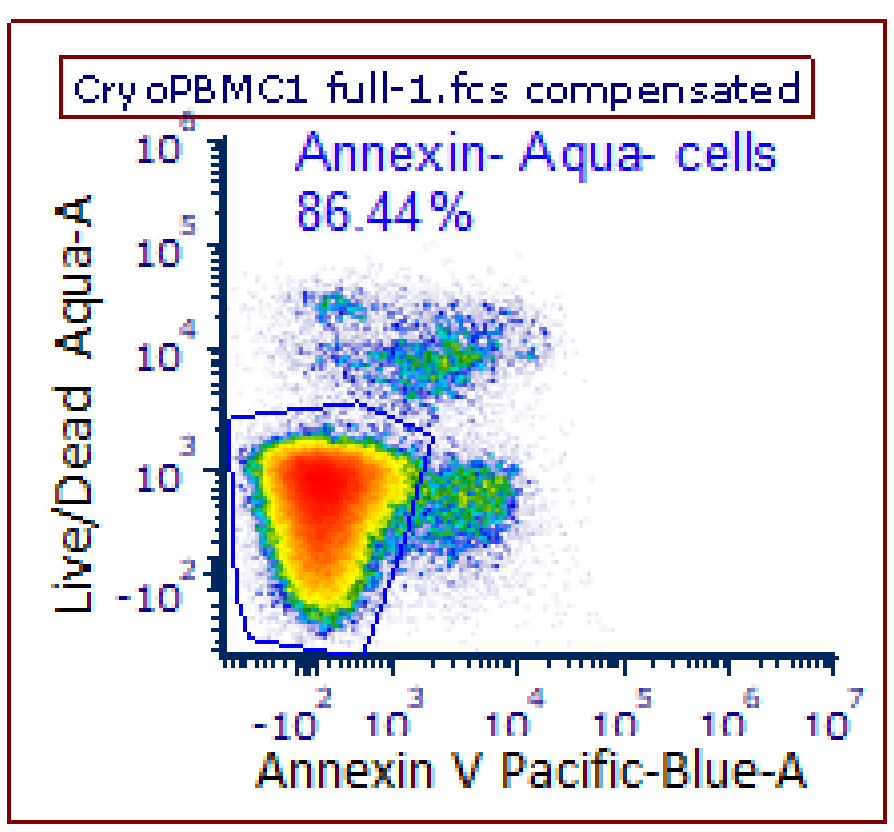

NOTE: QC synthetic cell Trucytes will not be assessed for viability or apoptosis. A separate analysis scheme will be applied, matching as much as feasible the analysis of the test sample.

NOTE: No viability threshold criteria will be applied to test sample. An abnormally low viability should be highlighted to the NIST but data for these must be entered in the spreadsheet.

Plot 5

A bivariate dot plot, CD45 PerCP-Cy5.5-A (X-axis) versus SSC-A (Y-axis), of 'Annexin-Aqua- cells'. This plot is used to draw a region around CD45+ cells that are live and non-apoptotic, named as "CD45+ mononuclear cells".

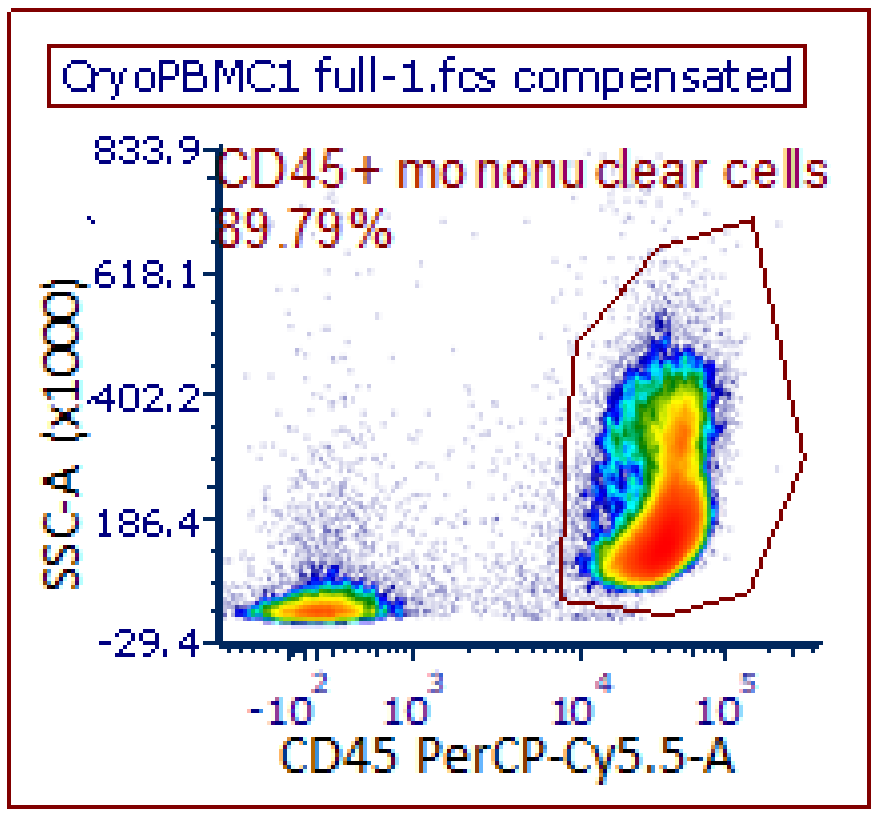

Plot 6

A bivariate dot plot, CD14/CD19 APC-A (X-axis) versus SSC-A (Y-axis), of the "CD45+ mononuclear Cells" gate. This plot is used to draw regions around the CD14bright monocytes, named "CD14+ dump monocytes". This gate identifies CD14^{bright} monocytes and include both the SSC^{med} (normal monocytes) and SSC^{low} (degranulated monocytes). The scope is to capture any degranulated monocytes in order to be able to subtract them for the 'Lymphocytes' gate from Plot 7. Note that the below sample does not contain degranulated monocytes but their expected location is noted with an *.

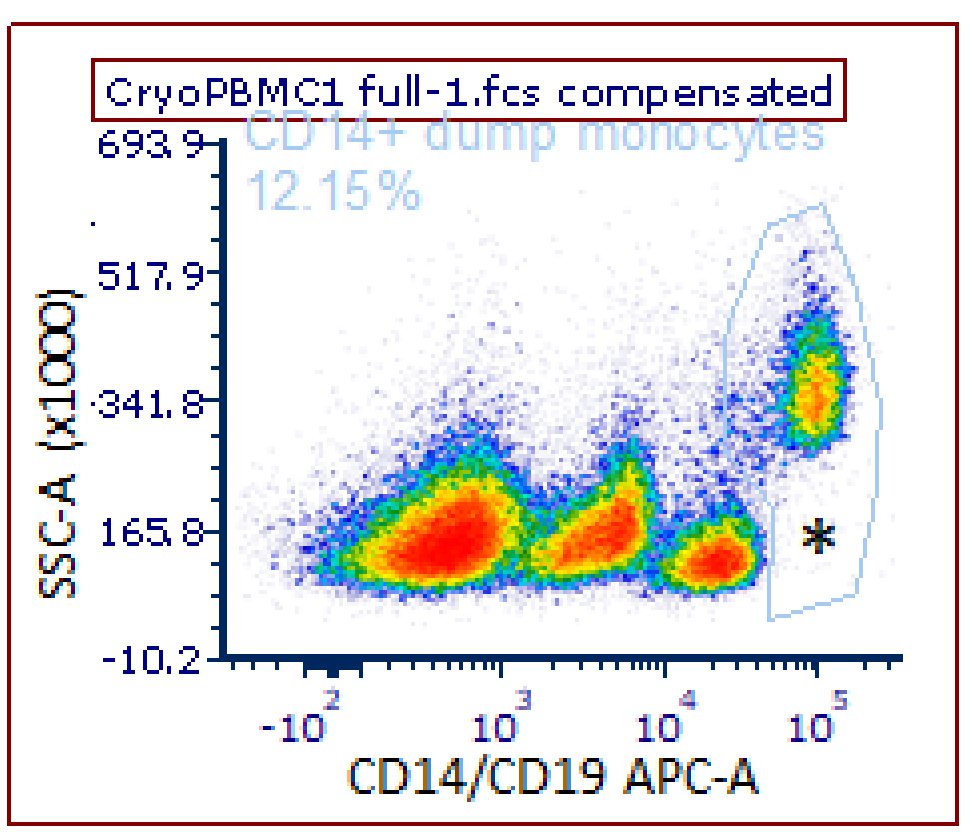

Plot 7

A bivariate dot plot, FSC-A (X-axis) versus SSC-A (Y-axis), of the ‘CD45+ mononuclear cells’ gate. This plot is used to draw regions around the following cell subsets: - “Lymphocytes” (CD45+, FSC^{low}, SSC^{low}), - “Monocytes” (CD45+, FSC^{med}, SSC^{med}).

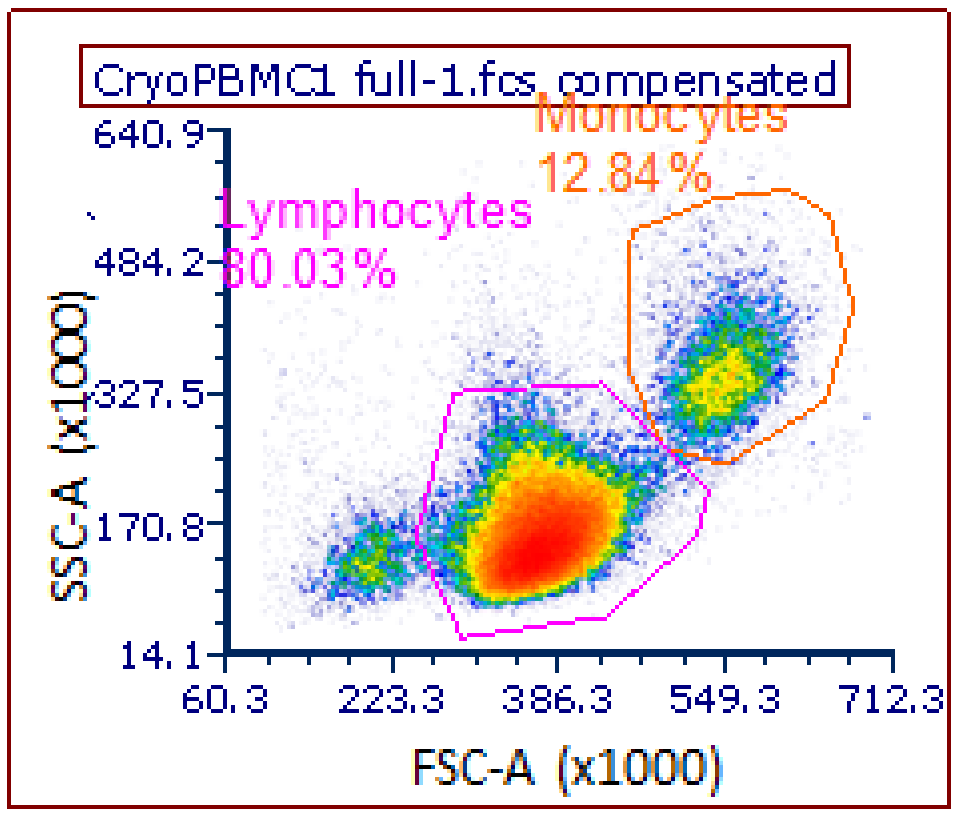

BOOLEAN GATING FOR LYMPHOCYTES GATE

In order to prevent potential contamination from degranulated monocytes, which may contaminate the lymphocyte gate, a boolean gating strategy is applied downstream of the ‘Lymphocytes’ gate. The events from the ‘CD14+ dump monocytes’ are substracted from the ‘Lymphocytes’ gate with the following Boolean equation: Lymphocytes AND NOT ‘CD14+ dump monocytes’.

QCing the Boolean formula: Ensure no APC^{bright} cells appear in Plot 10 as correct analysis and Boolean gating would remove these cells from appearing in downstream Lymphocytes plots.

Plot 8

A bivariate dot plot, CD3 FITC-A (X-axis) versus CD16/CD56 PE-A (Y-axis), of the ‘Lymphocytes’ gate. This plot is used to draw regions around “CD3+ T cells” ($CD45^+$, FSC^{low}, SSC^{low}, $CD3^+$, $CD16/CD56^-$) and “NK cells” ($CD45^+$, FSC^{low}, SSC^{low}, $CD3^-$, $CD16/CD56^+$).

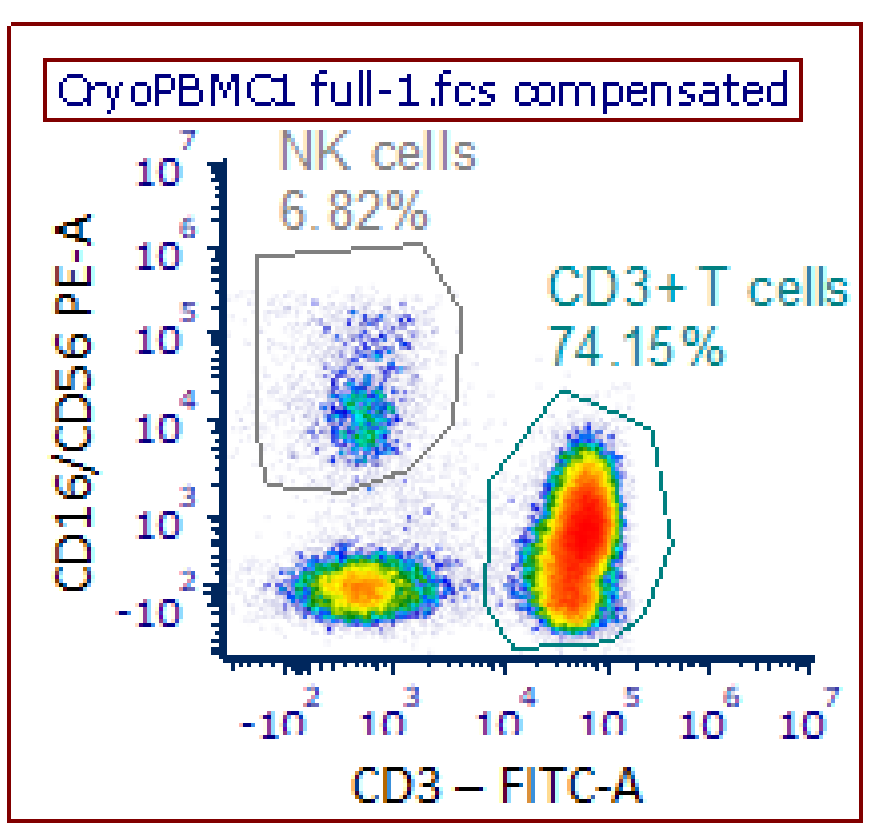

Plot 9

A bivariate dot plot, CD8 APC-Cy7-A (X-axis) versus CD4 PE-Cy7-A (Y-axis), of the 'CD3+ T cells' gate. This plot is used to draw regions around the following cell subsets: - "CD8+ T cells" ($CD45^+$, FSC^{low}, SSC^{low}, $CD3^+$, $CD8^+$) and "CD4+ T cells" ($CD45^+$, FSC^{low}, SSC^{low}, $CD3^+$, $CD4^+$).

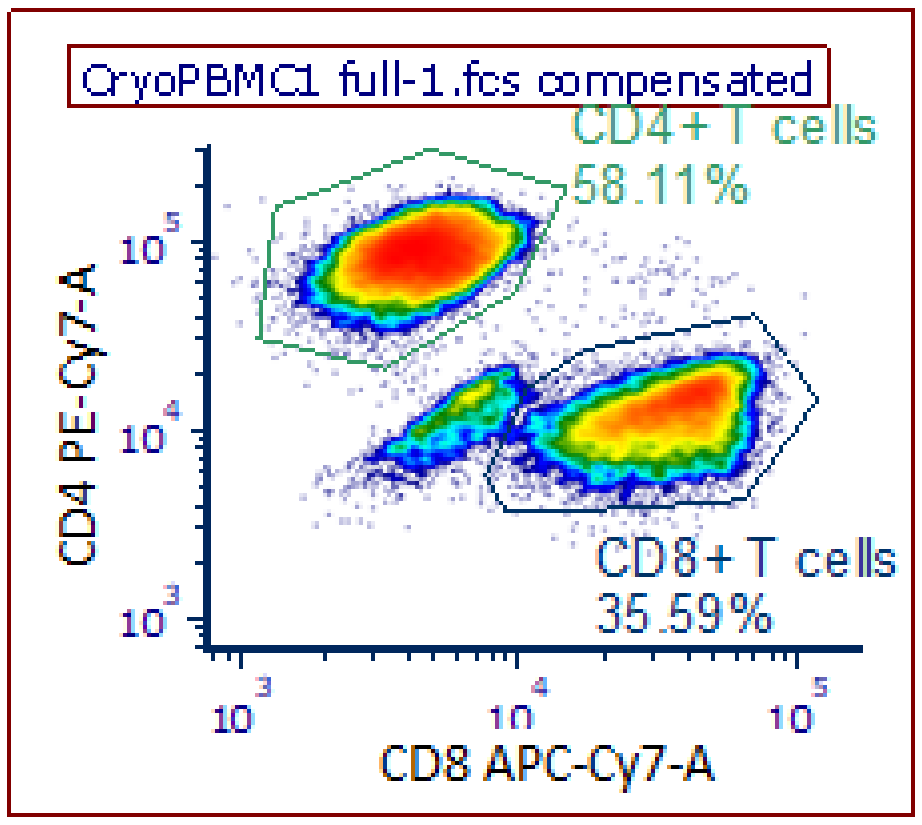

Plot 10

A bivariate dot plot, CD3 FITC-A (X-axis) versus CD19 (CD14) APC-A (Y-axis), of the "Lymphocytes" gate defined in Plot 7. This plot is used to draw regions around the "CD19+ B cells" subset ($CD45^+$, FSC^{low}, SSC^{low}, $CD3^-$, $CD19^+$).

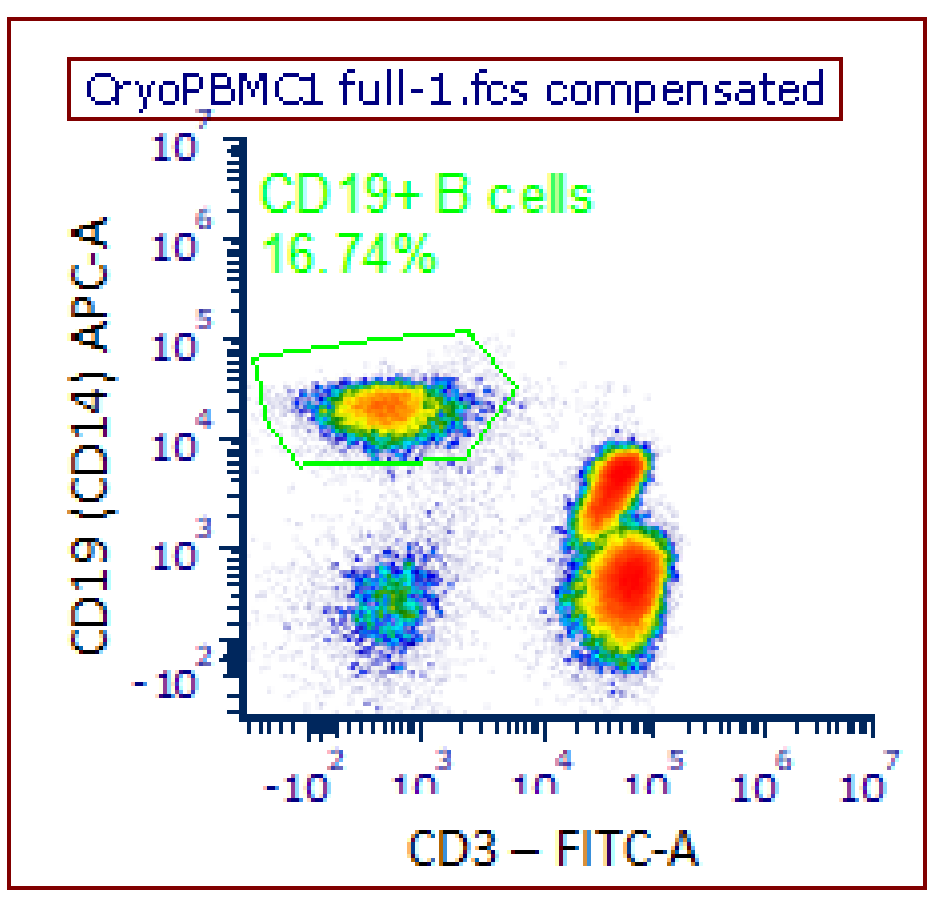

Plot 11

A bivariate dot plot, CD3 FITC-A (X-axis) versus CD14 (CD19) APC-A (Y-axis), of the "Monocytes" gate defined in Plot 7. This plot is used to draw region around "CD14+ monocytes". Ensure to only include the CD14^{bright} cells similarly to Plot 6.

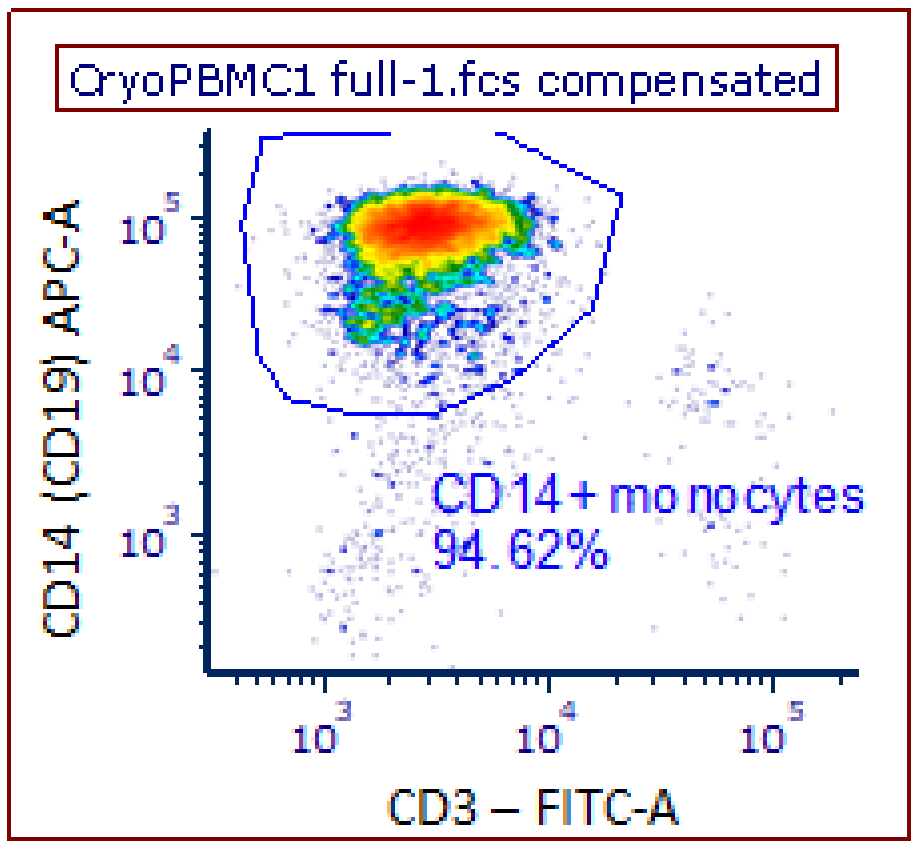

Population Hierarchy

Population hierarchy for the TBNMK tube is provided below.

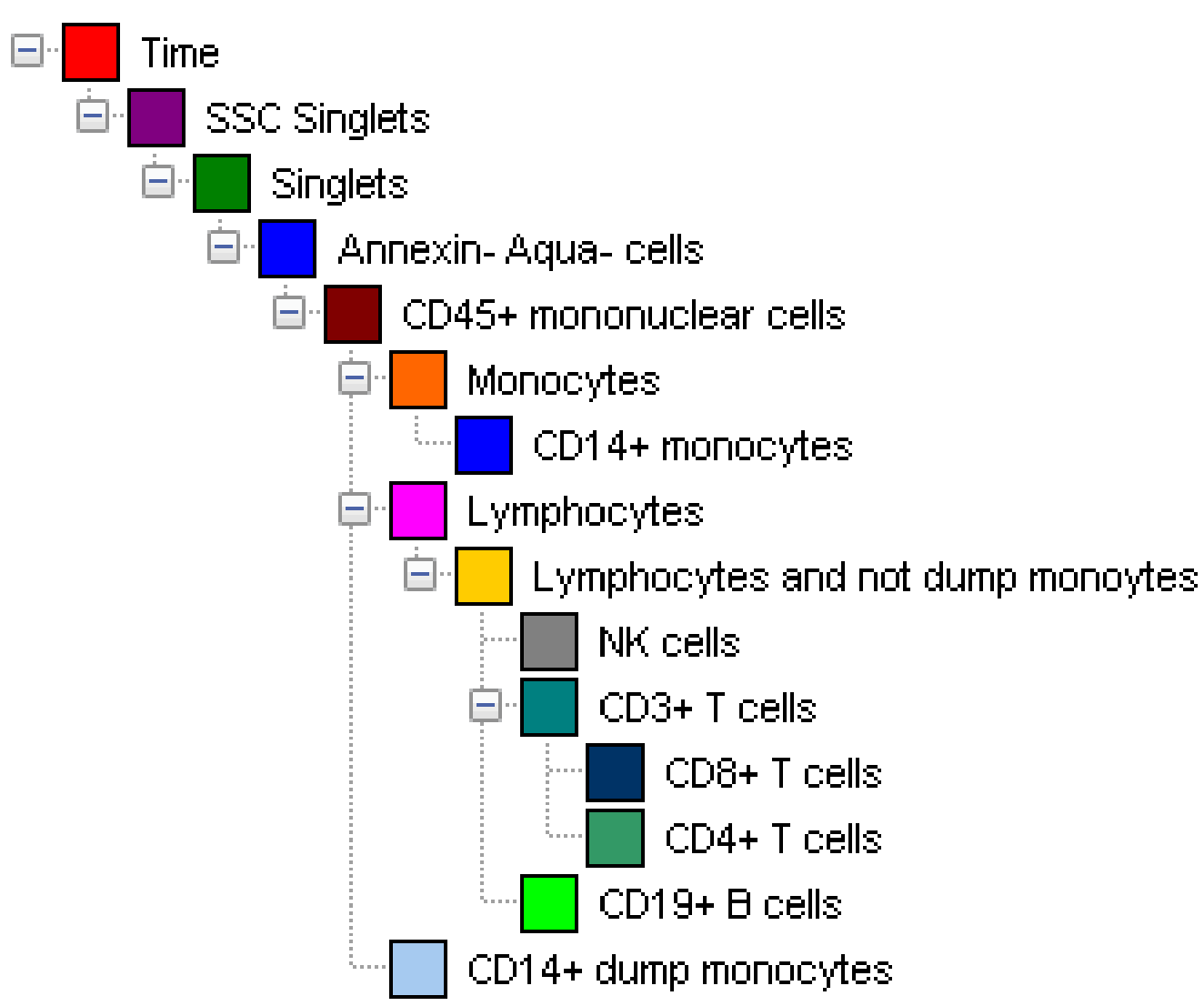

1.3. Analysis template for Trucount™ counting tube for QC synthetic cell Trucytes

Plot 1

A bivariate dot plot, Time (X-axis) versus CD45 PerCP-Cy5.5-A (Y-axis), of all collected events. This plot is used for acquisition quality monitoring where all populations are equally present.

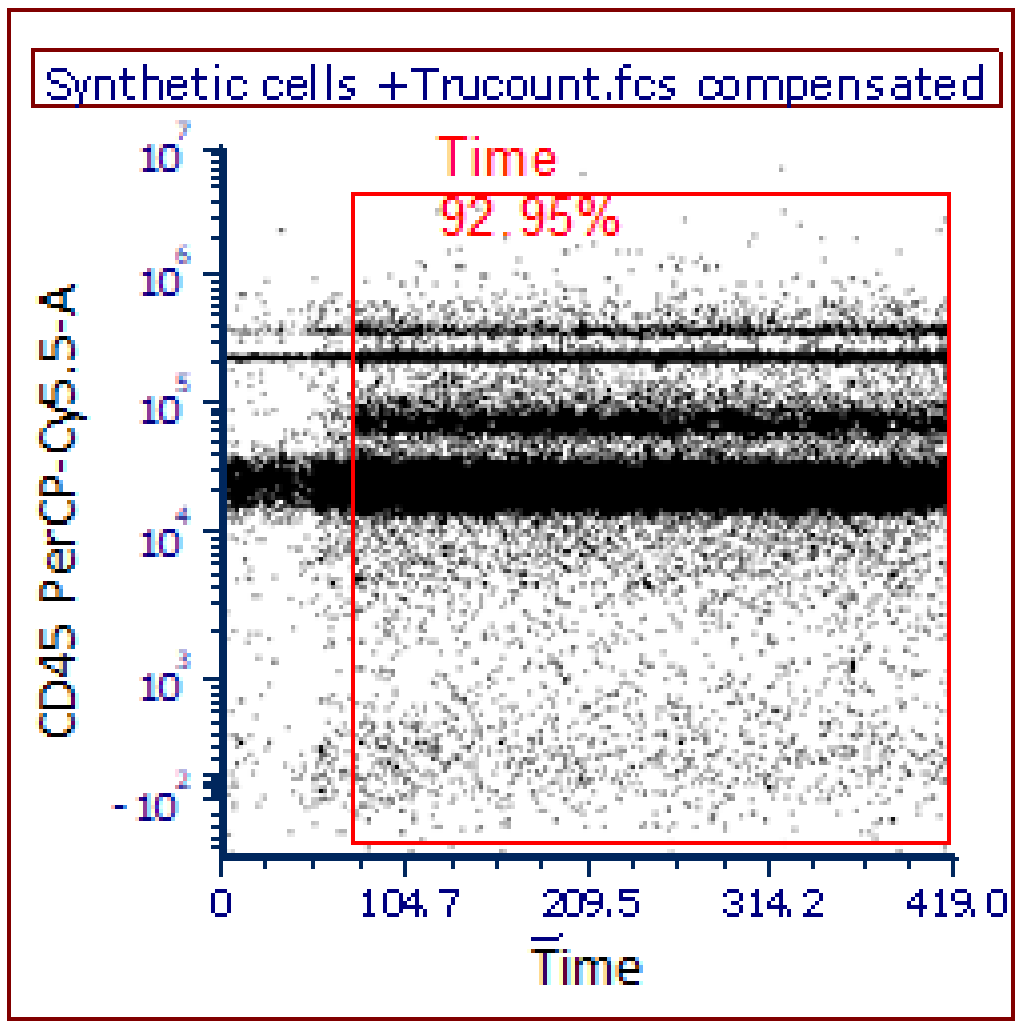

Plot 2

A bivariate dot plot, APC-A (X-axis) versus SSC-A (Y-axis), of events in 'Time' gate. This plot is used to draw a region around the "Beads" and the cellular events "Not beads". Ensure all beads are taken in the gate. Note specific APC fluorescence is not detected because all cellular events are APC negative. Beads autofluorescence is used to detect and gate them.

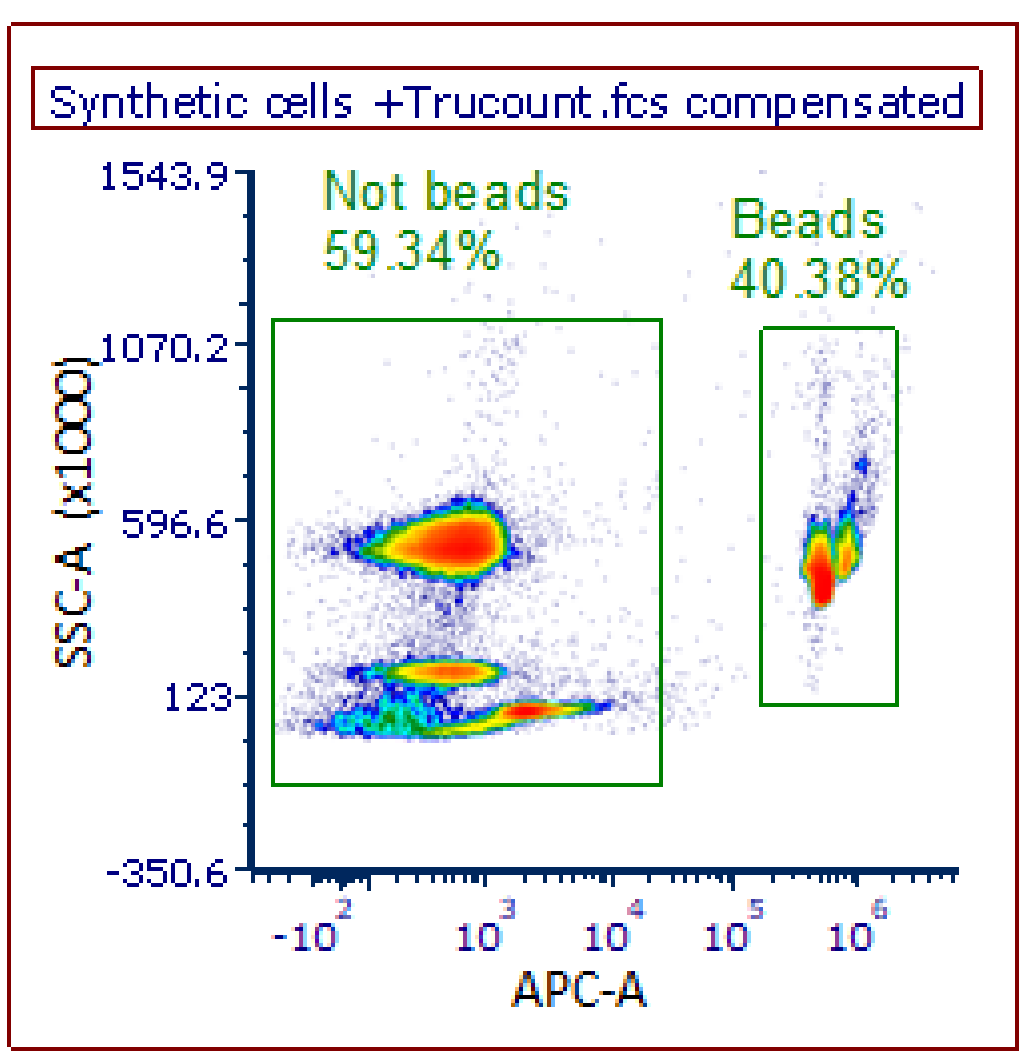

Plot 3

A bivariate dot plot, SSC-A (X-axis) versus SSC-H (Y-axis), of “Not beads”. This plot is used to draw a region around single cells to exclude side scatter doublets, named “SSC Singlets”. Note that scattering profiles of Trucytes are different from cryoPBMC.

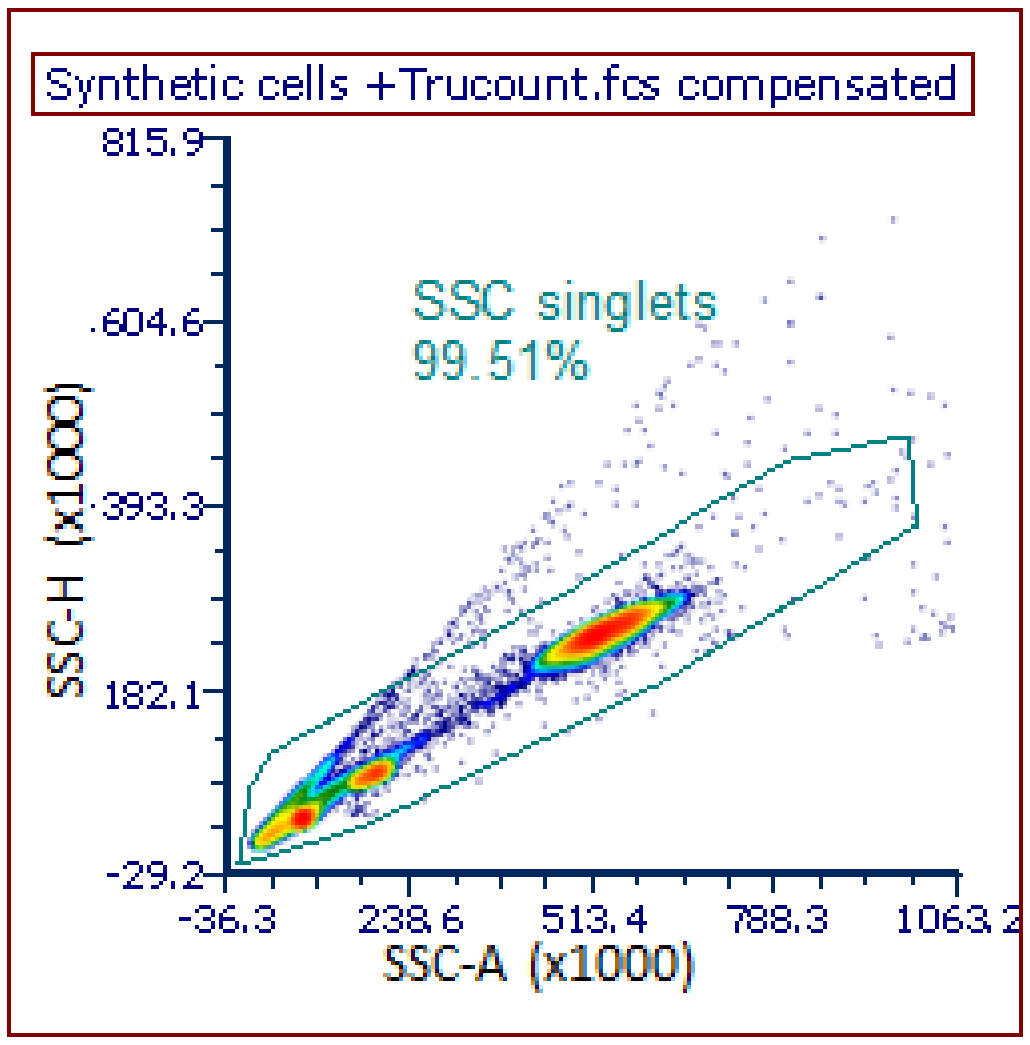

Plot 4

A bivariate dot plot, FSC-A (X-axis) versus FSC-H (Y-axis), of events in ‘SSC singlets’ gate. This plot is used to draw a region around single cells to exclude forward scatter doublets, named “Singlets”. Note that scattering profiles of Trucytes are different from cryoPBMC.

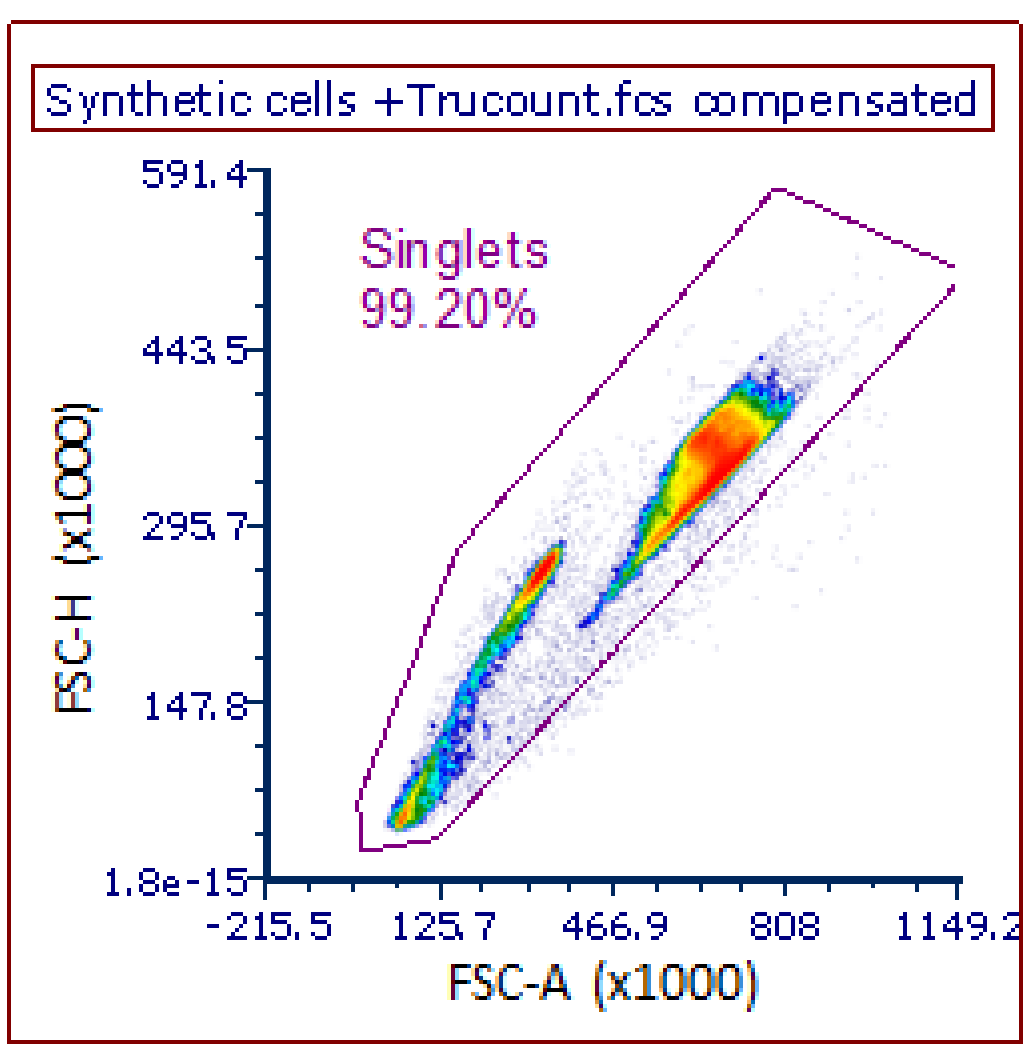

Plot 5

A bivariate dot plot, CD45 PerCP-Cy5.5-A (X-axis) versus SSC-A (Y-axis), of events in 'Singlets' gate. This plot is used to draw a region around the "CD45+ mononuclear cells". The events in this gate will be used for the calculation of absolute counts. Note that Trucytes include granulocytes (CD45+ SSC^{high}) that are outside of the "CD45+ mononuclear cells" gate.

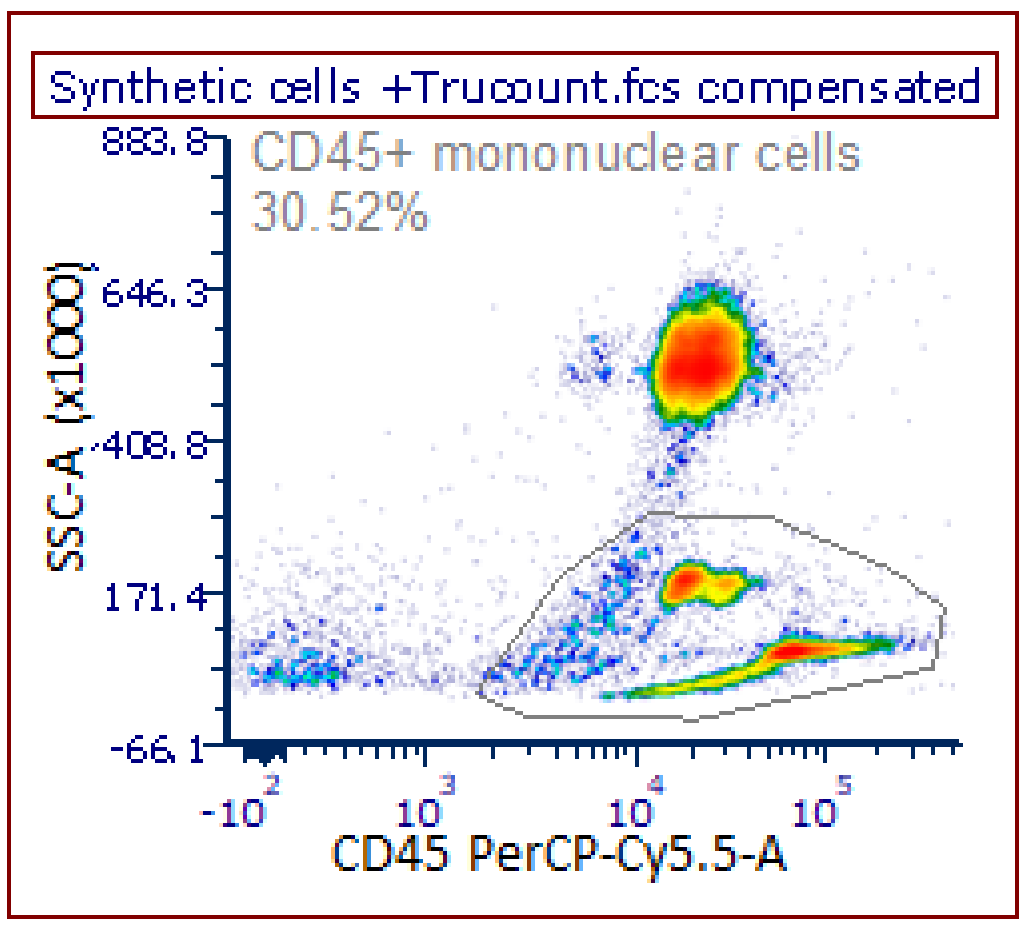

Population Hierarchy

Population hierarchy for the counting tube is provided below.

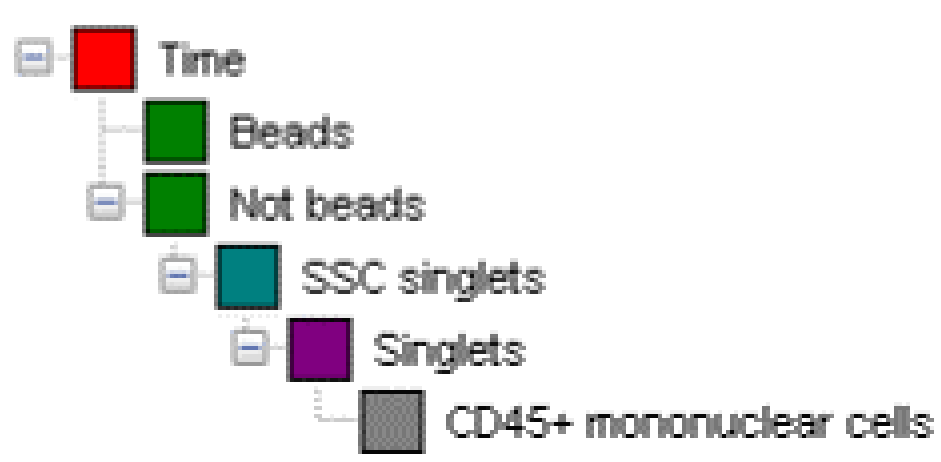

1.4. Analysis template for TBMNK panel for QC synthetic cell Trucytes

Plot 1

A bivariate dot plot, time (X-axis) versus CD3 FITC-A (Y-axis), of all collected events. This plot is used for acquisition quality monitoring.

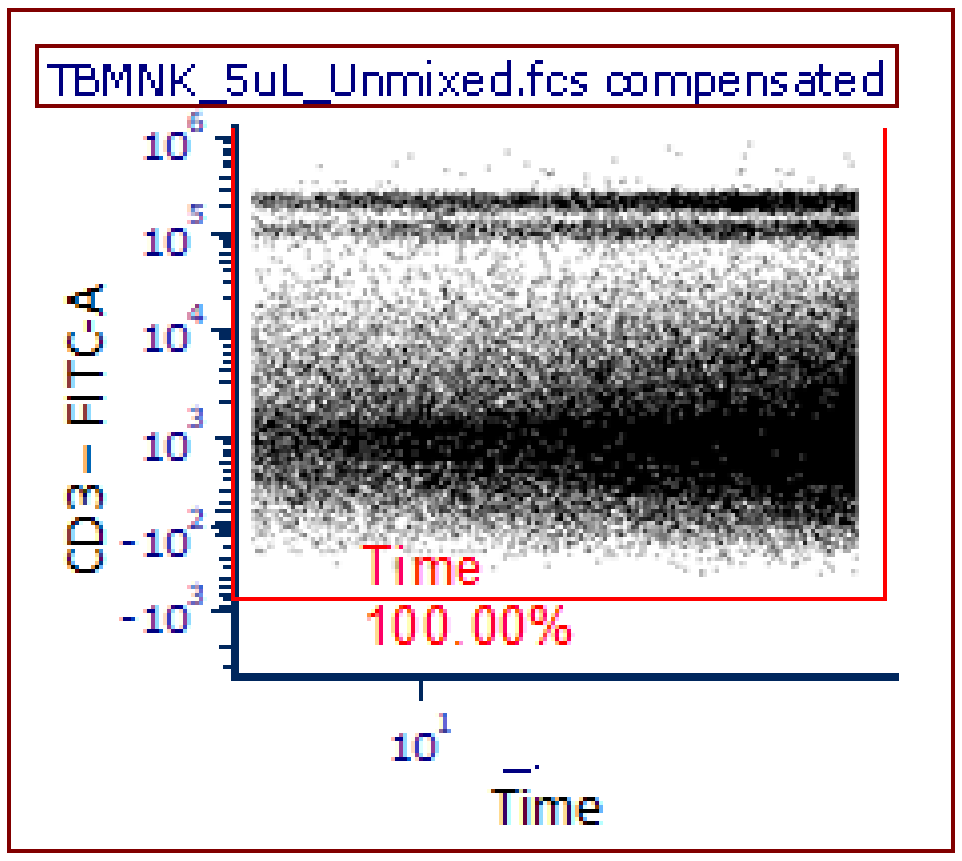

Plot 2

A bivariate dot plot, SSC-A (X-axis) versus SSC-H (Y-axis), of events in ‘Time’ gate. This plot is used to draw a region around single cells to exclude side scatter doublets, named “SSC Singlets”. Do not exclude debris that are SSC^{low} at this stage since they are gated out in downstream Plot 4.

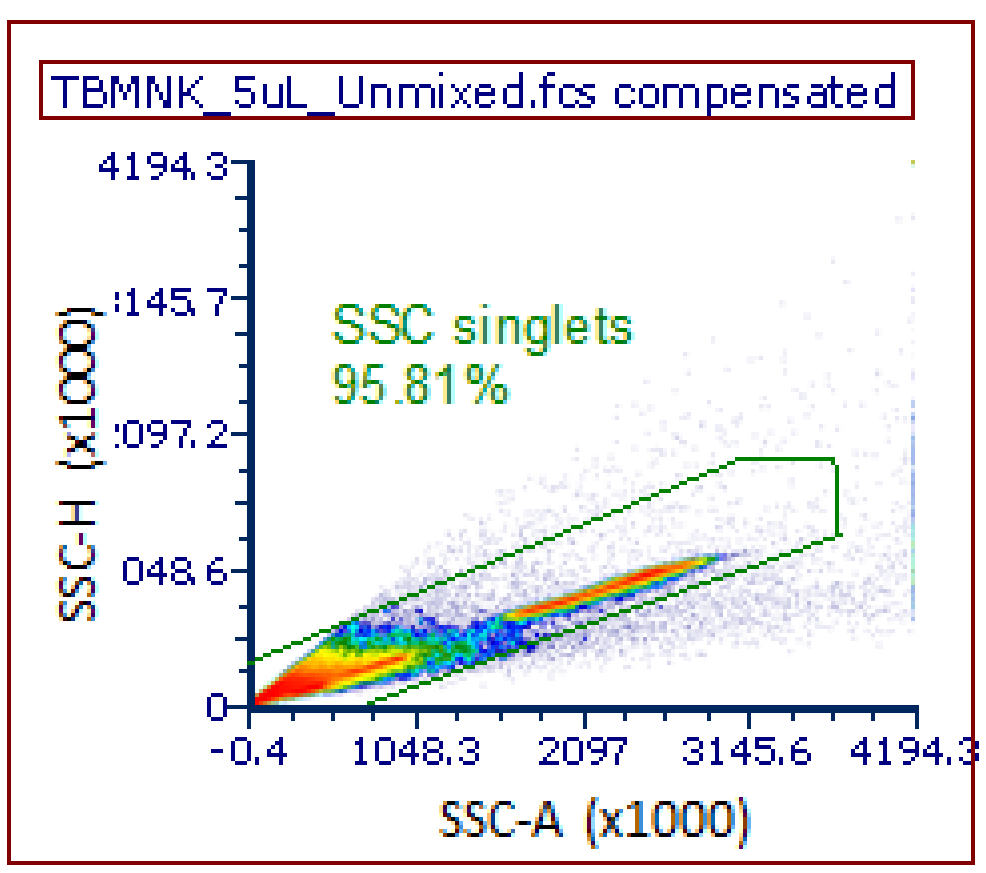

Plot 3

A bivariate dot plot, FSC-A (X-axis) versus FSC-H (Y-axis), of events in 'SSC singlets' gate. This plot is used to draw a region around single cells to exclude forward scatter doublets, named "Singlets". Do not exclude debris that are FSC^{low} at this stage since they are gated out in downstream Plot 4.

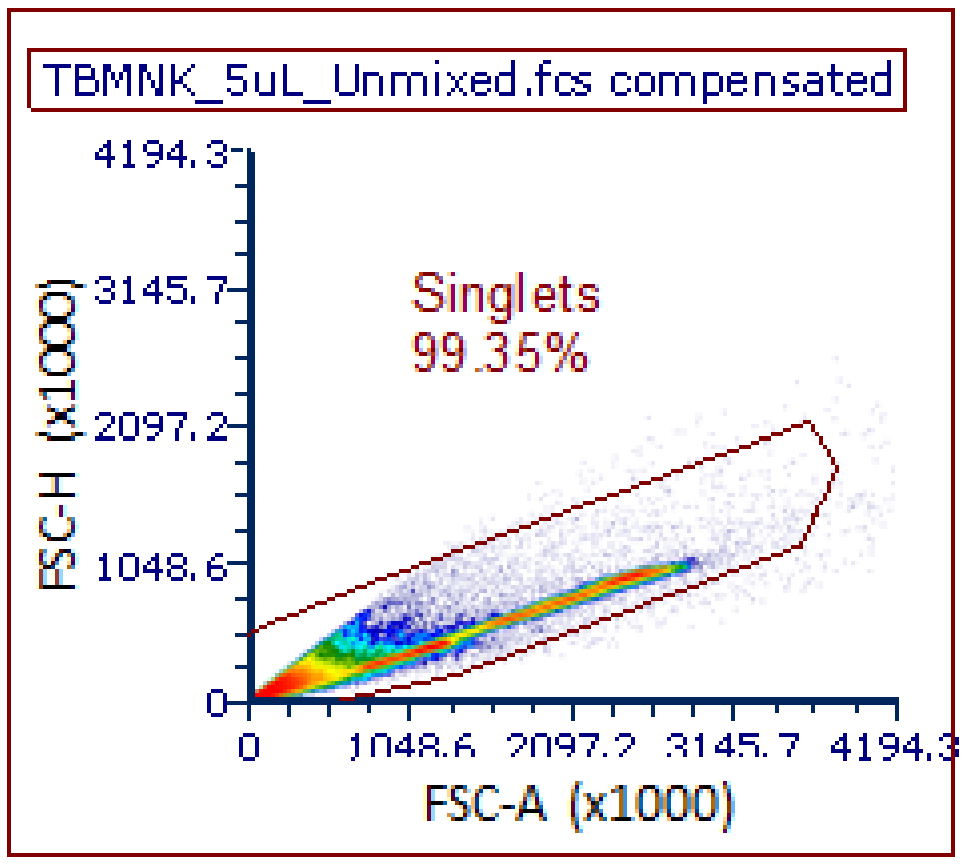

Plot 4

A bivariate dot plot, CD45 PerCP-Cy5.5-A (X-axis) versus SSC-A (Y-axis), of 'CD45+ cells. This plot is used to draw a region around all of the CD45+ cells to exclude debris and generate a reliable gate for reporting daughter 'monocytes' and 'lymphocytes' populations. The gate is named "CD45+ mononuclear cells ". Note that Trucytes include granulocytes (CD45+ SSC^{high}) that are above the "CD45+ mononuclear cells" gate.

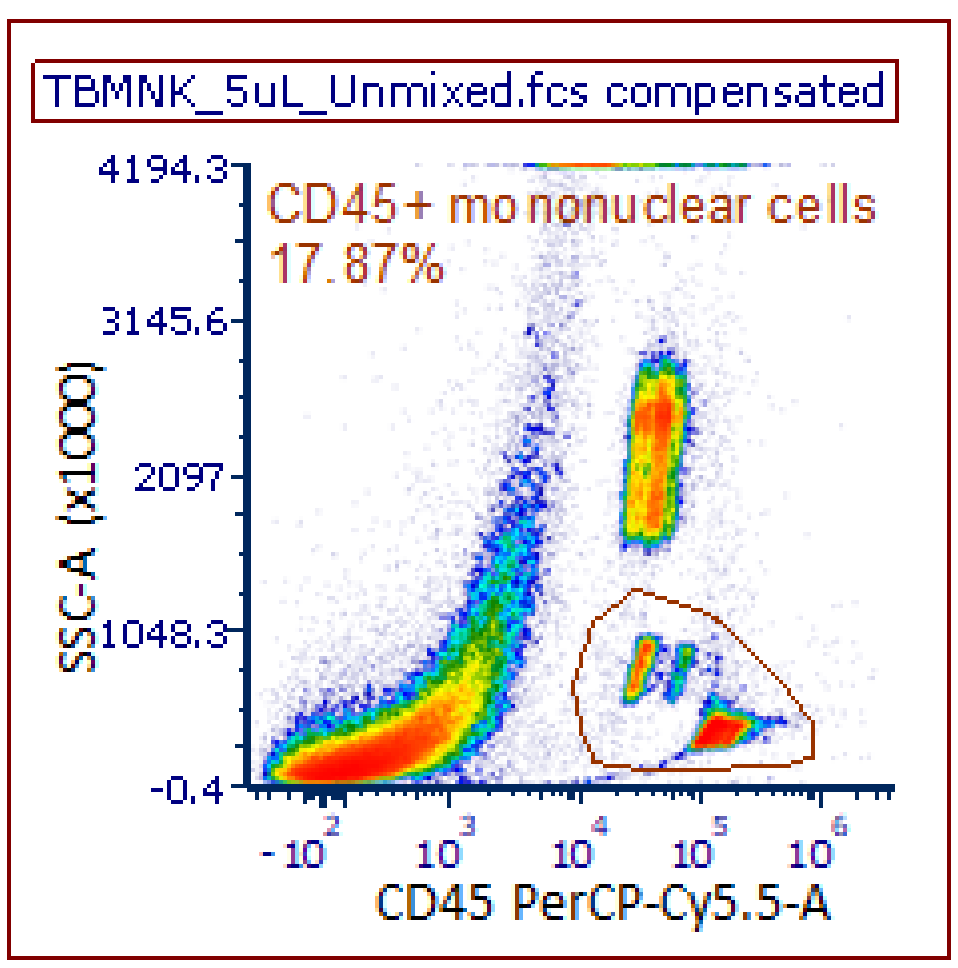

Plot 5

A bivariate dot plot, CD45 PerCP-Cy5.5-A (X-axis) versus SSC-A (Y-axis), of 'C'. This plot is used to draw regions around the following cell subsets: - "Lymphocytes" ($CD45^{bright}$, SSC^{low}), - "Monocytes" ($CD45^{+}$, SSC^{med}). Note that the monocytes of Trucytes consist of two distinct populations, classical and non-classic monocytes (also see Slingshot TDS for Trucytes TBMNK).

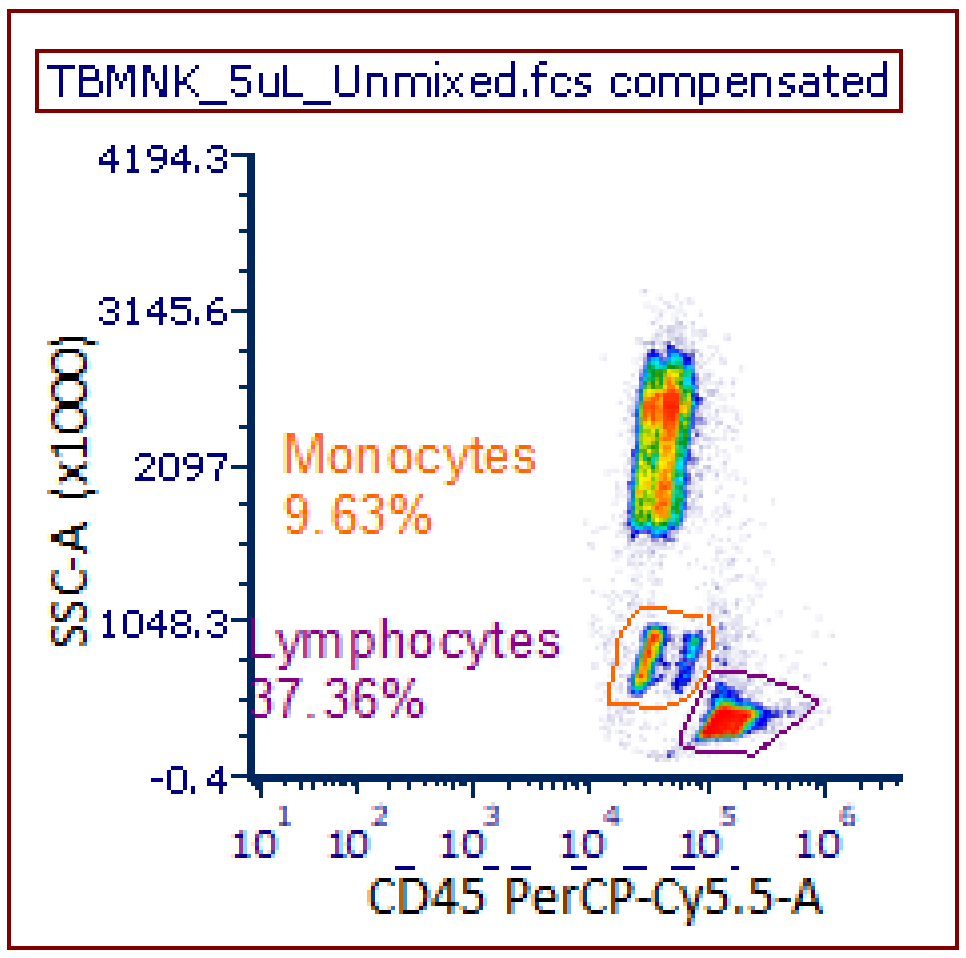

Plot 6

A bivariate dot plot, CD3 FITC-A (X-axis) versus CD16/CD56 PE-A (Y-axis), of the 'Lymphocytes' gate. This plot is used to draw regions around "CD3+ T cells" ($CD45^{bright}$, SSC^{low}, $CD3^{+}$, $CD16/CD56^{-}$) and "NK cells" ($CD45^{bright}$, SSC^{low}, $CD3^{-}$, $CD16/CD56^{+}$). Note that the CD3+ lymphocytes of Trucytes consist of two distinct populations, CD4+ and CD8+ lymphocytes (also see Slingshot TDS for Trucytes TBMNK).

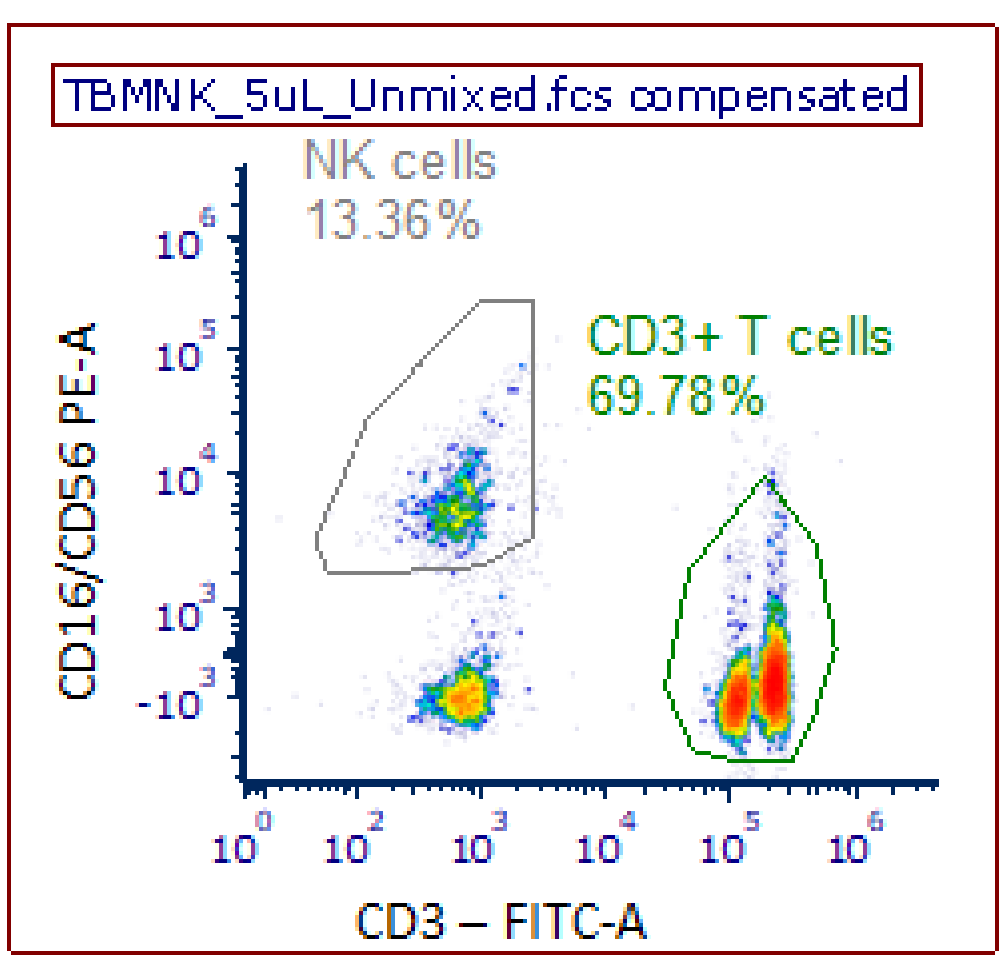

Plot 7

A bivariate dot plot, CD8 APC-Cy7-A (X-axis) versus CD4 PE-Cy7-A (Y-axis), of the ‘CD3+ T cells’ gate. This plot is used to draw regions around the following cell subsets: - “CD8+ T cells” ($CD45^{bright}$, SSC^{low}, $CD3^{+}$, $CD8^{+}$) and “CD4+ T cells” ($CD45^{bright}$, SSC^{low}, $CD3^{+}$, $CD4^{+}$).

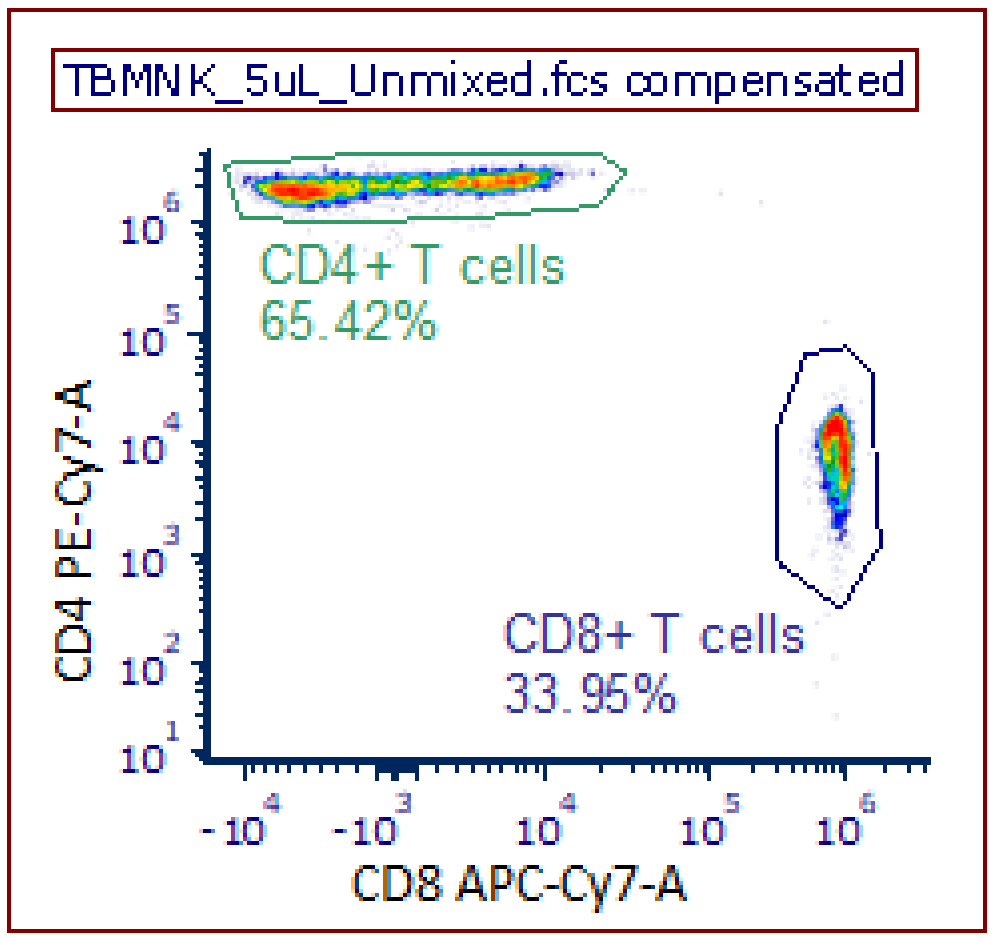

Plot 8

A bivariate dot plot, CD3 FITC-A (X-axis) versus CD19 (CD14) APC-A (Y-axis), of the “Lymphocytes” gate. This plot is used to draw regions around the “CD19+ B cells” subset ($CD45^{bright}$, SSC^{low}, $CD3^{-}$, $CD19^{+}$).

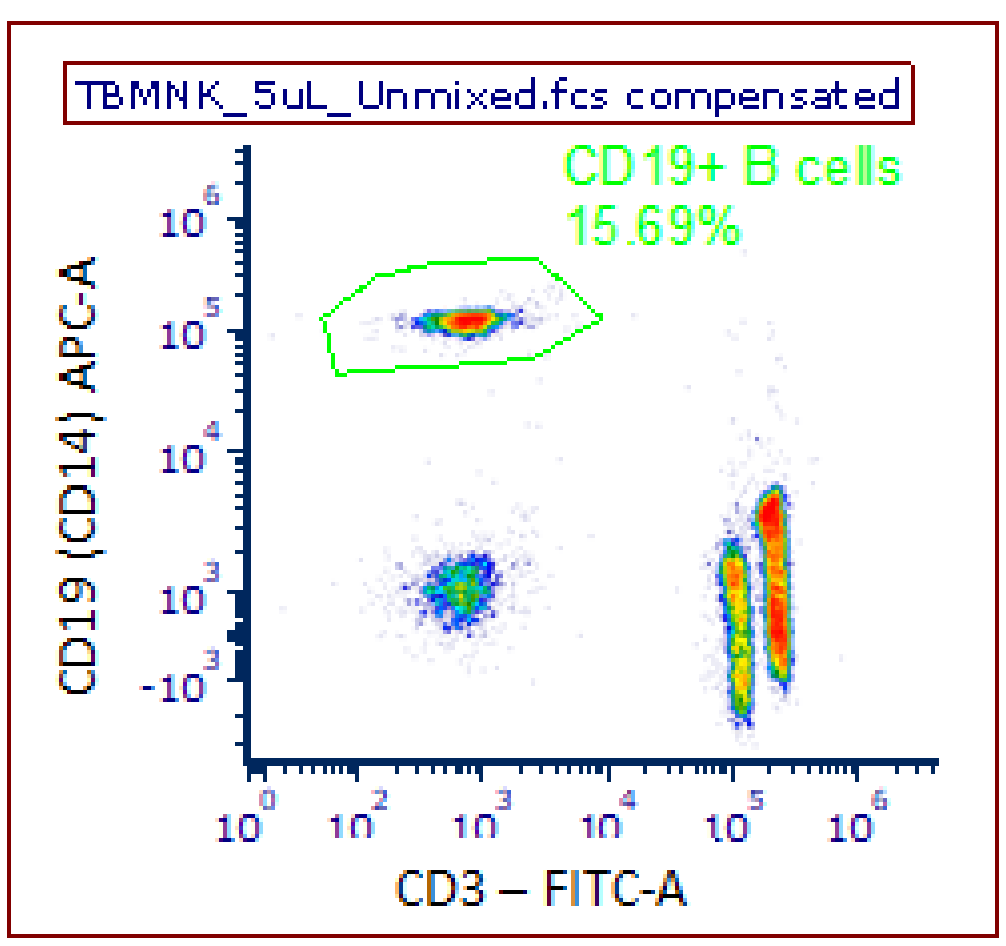

Plot 9

A bivariate dot plot, CD3 FITC-A (X-axis) versus CD14 (CD19) APC-A (Y-axis), of the "Monocytes" gate. This plot is used to draw region around "CD14+ monocytes".

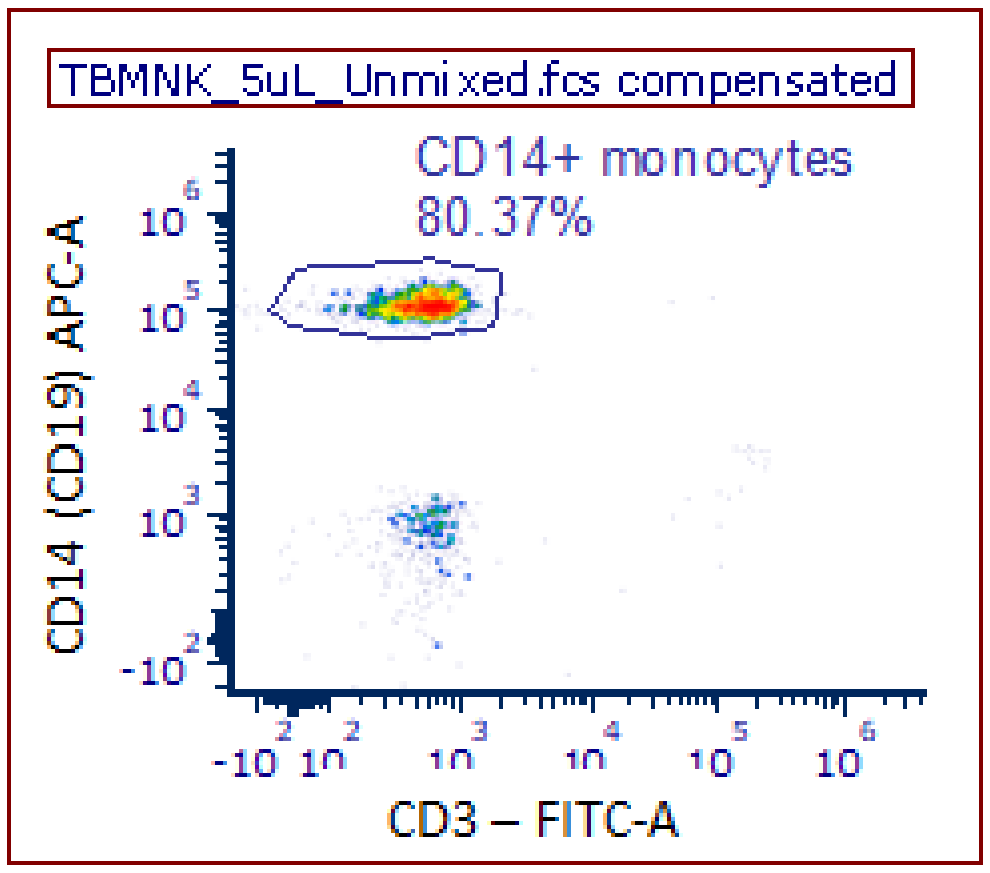

Population Hierarchy

Population hierarchy for the TBNMK tube for QC synthetic cell Trucytes is provided below.

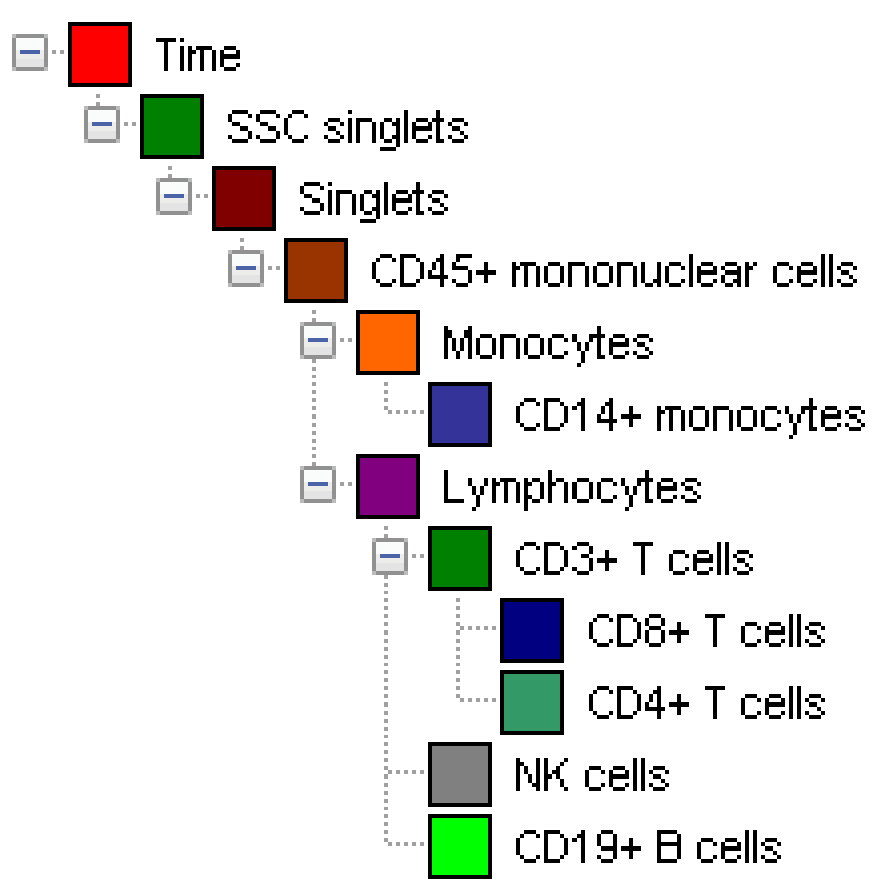

1.5. WG2 ILS Reporting Spreadsheet

Summary population hierarchy and associated assay reportable and values results needed to generate data to enter in the WG2 ILS Reporting Spreadsheet are listed in Table 1 and Table 2.
The list of reportable results for the test sample and synthetic cells QC material are listed in the below sections.

Table 1. Values to record to obtain reportables for counting tube

Name of population in analysis template	List of parameters to record
Time	NA
Beads	Events*
Not beads	NA
SSC Singlets	NA
Singlets	NA
Annexin-Aqua- cells (for cryo PBMC only)	NA
CD45+ mononuclear cells	Events*

*Will be used for absolute counts calculations

Table 2. Values to record to obtain reportables for immunophenotyping tube

Name of population in analysis template	List of parameters to record			
Time	NA			
SSC Singlets	NA			
Singlets	NA			
Annexin-Aqua- cells (for cryo PBMC only)	% parent	NA	NA	NA
CD45+ mononuclear cells	% parent**	Events*	MdFI PerCP-Cy5.5	rSD PerCP-Cy5.5
Monocytes	% parent	NA	NA	NA
CD14+ monocytes	% parent	Events*	MdFI APC	rSD APC
Lymphocytes	NA			
Lymphocytes and not dump monocytes	% grandparent	NA	NA	NA
NK cells	% parent	Events*	MdFI PE	rSD PE
CD3+ T cells	% parent	Events*	MdFI FITC	rSD FITC
CD8+ T cells	% parent	Events*	MdFI APC-Cy7	rSD APC-Cy7
CD4+ T cells	% parent	Events*	MdFI PE-Cy7	rSD PE-PE-Cy7
CD19+ B cells	% parent	Events*	MdFI APC	rSD APC
CD14+ dump monocytes	NA			

*Will be used for absolute counts calculations

**For cryoPBMC only

1.6. Summary population hierarchy and associated assay reportable results to enter in the WG2 ILS Reporting Spreadsheet

Population Hierarchy is provided in Section 1.2 and the list of reportable results for each sample and QC material are listed in Tables 3 and 4.

Table 3: Test Sample (cryoPBMC) Reportable Results

population	Immunophenotype	Reporting Unit
Annexin-Aqua- cells	Aqua-, Annexin-	% of singlets (% parent)
CD45+ mononuclear cells	Aqua-, Annexin-, CD45+	% Annexin-Aqua- cells (% parent)
		cells/uL*
		MdFI and rSD CD45 PerCP-Cy5.5
Monocytes	Aqua-, Annexin-, CD45+, FSCmed, SSCmed	% CD45+ cells (% parent)
CD14+ monocytes	Aqua-, Annexin-, CD45+, FSCmed, SSCmed, CD14+/hi	% monocytes (% parent)
		cells/uL*
		MdFI and rSD CD14 APC
Lymphocytes And Not Dump Monocytes	Aqua-, Annexin-, CD45+, FSClow, SSClow	% CD45+ cells (% grandparent)
CD3+ T cells	Aqua-, Annexin-, CD45+, FSClow, SSClow, CD3+	% of Lymphocytes And Not Dump Monocytes (% parent)
		cells/uL*
		MdFI and rSD CD3 FITC
CD4+ T cells	Aqua-, Annexin-, CD45+, FSClow, SSClow, CD3+, CD4+	% of CD3+ T cells (% parent)
		cells/uL*
		MdFI and rSD CD4 PE-Cy7
CD8+ T cells	Aqua-, Annexin-, CD45+, FSClow, SSClow, CD3+, CD8+	% of CD3+ T cells (% parent)
		cells/uL*
		MdFI and rSD CD8 APC-Cy7
CD19+ B cells	Aqua-, Annexin-, CD45+, FSClow, SSClow, CD19+	% of Lymphocytes And Not Dump Monocytes (% parent)
		cells/uL*
		MdFI and rSD CD19 APC
NK cells	Aqua-, Annexin-, CD45+, FSClow, SSClow, CD56+/CD16+:	% of Lymphocytes And Not Dump Monocytes (% parent)
		cells/uL*
		MdFI and rSD CD56 CD16 PE

*absolute counts may be derived using volumetric counting or Trucount™ tube method

Table 4. QC Synthetic cell Trucytes Reportable Results

population	Immunophenotype	Reporting Unit
CD45+ mononuclear cells	CD45+	NA (no % will be reported)
		cells/uL*
		MdFI and rSD CD45 PerCP-Cy5.5
Monocytes	CD45+, SSCmed	% CD45+ cells % parent)
CD14+ monocytes	CD45+, SSCmed, CD14+	% monocytes (% parent)
		cells/uL*
		MdFI and rSD CD14 APC

Lymphocytes	CD45bright, SSClow	% CD45+ cells (% parent)
CD3+ T cells	CD45bright, SSClow, CD3+	% of Lymphocytes (% parent)
		cells/uL*
		MdFI and rSD CD3 FITC
CD4+ T cells	CD45bright, SSClow, CD3+, CD4+	% of CD3+ T cells (% parent)
		cells/uL*
		MdFI and rSD CD4 PE-Cy7
CD8+ T cells	CD45bright, SSClow, CD3+, CD8+	% of CD3+ T cells (% parent)
		cells/uL*
		MdFI and rSD CD8 APC-Cy7
CD19+ B cells	CD45bright, SSClow, CD19+	% of Lymphocytes (% parent)
		cells/uL*
		MdFI and rSD CD19 APC
NK cells	CD45bright, SSClow, CD56+/CD16+:	% of Lymphocytes (% parent)
		cells/uL*
		MdFI and rSD CD56 CD16 PE

*absolute counts may be derived using volumetric counting or Trucount™ tube method

1.7. Analysis Procedure for Counting Tube

- Import compensated .fcs files for the Test Samples and the QC material into counting tube analysis template (analysis template guidelines described in Section 1.1 and Appendix A and in Section 1.3 and Appendix C).
- **NOTE**: Compensated Matrix 1 generated with cryoPBMC and lyoLeuk is suggested to be applied to the counting tubes for the test samples and QC material as specified in the WG2 ILS Reporting Spreadsheet.
- Analyze data files as described in Section 1.1 and 1.3 in order to derive CD45+ cells absolute counts. Generate pdf of the batch files and enter reportable results in WG2 ILS Reporting Spreadsheet format.
- Generate pdf of the batch files and use events numbers in order to calculate absolute.

1.7.1. Analysis Procedure for TBMNK Panel Compensation Matrix 1

- Import uncompensated .fcs files for the Test Samples and the QC material into analysis template (analysis template guideline described in Section 1.2 and Appendix B and in Section 1.4 and Appendix D)
- Apply compensation matrix 1 to all of the files (FMOs and fully stained tubes)
- Set gates as described in Section 1.2 for the full stain tube and cross verify that the gates are appropriate by verifying the FMO pattern.
- Once gets are set, run analysis on full stain triplicates as a batch without changing gates between replicates.
- Generate pdf of the batch files and enter reportable results for % of parent in WG2 ILS Reporting Spreadsheet format.
- Follow sections 1.7.4, 1.7.5, and 1.8 for the absolute counts and ERF calculations.

1.7.2. Compensation Matrix 2

- Import uncompensated .fcs files for the Test Samples and the QC material into appropriate analysis template (analysis guideline described in Section 4.2 and Appendix B and in Section 1.4 and Appendix D).
- Apply compensation matrix 2 to all of the files (FMOs and fully stained tubes).
- Set gates as described in Section 1.2 for the full stain tube and cross verify that the gates are appropriate by verifying the FMO pattern.
- Once gets are set, run analysis on full stain triplicates as a batch without changing gates between replicates.
- Generate pdf of the batch files and enter reportable results for % of parent in WG2 ILS Reporting Spreadsheet format.
- Follow sections 1.7.4, 1.7.5, and 1.8 for the absolute counts and ERF calculations.

1.7.3. Compensation Matrix 3

- Import uncompensated .fcs files for the Test Samples and the QC material into appropriate analysis template (analysis template guideline described in Section 4.2 and Appendix B and in Section 1.4 and Appendix D).
- Apply compensation matrix 3 to all of the files (FMOs and fully stained tubes).
- Set gates as described in Section 1.2 for the full stain tube and cross verify that the gates are appropriate by verifying the FMO pattern.
- Once gets are set, run analysis on full stain triplicates as a batch without changing gates between replicates.
- Generate pdf of the batch files and enter reportable results for % of parent in WG2 ILS Reporting Spreadsheet format.
- Follow sections 1.7.4, 1.7.5, and 1.8 for the absolute counts and ERF calculations.

1.7.4. Cell Counting using volumetric method

- For volumetric flow cytometers, cell count can be directly obtained from acquired data files showing a known analytical volume and event and percentage of the cell population.

1.7.5. Cell Counting using Trucount™ method

- Collect data from cell counting Trucount™ tube analysis pdf and perform the following calculation to determine the CD45+ cells absolute count in each sample and each compensation matrix

- $\text{CD45+ mononuclear cells cells/}\mu\text{L} = \frac{(\text{CD45+ mononuclear cells events})\times(\text{bead counts})*}{(\text{beads events})\times(100\ \mu\text{L sample volume used})}$

*lot specific and provided in manufacturer's package insert

- Once the CD45+ cell count has been determined, calculate absolute counts for each CD45+ cell subsets from the full stain tube.

T cells:

- $$\text{CD3+ T cells cells/}\mu\text{L} = \frac{(\text{CD45}^{+}\text{ mononuclear cells absolute count from counting tube}) \times (\text{CD3+ T cells events})}{(\text{CD45+ mononuclear cells events})}$$

CD4 T cells:

- $$\text{CD4+ T cells cells/}\mu\text{L} = \frac{(\text{CD45}^{+}\text{ mononuclear cells absolute count from counting tube}) \times (\text{CD4+ T cells events})}{(\text{CD45+ mononuclear cells events})}$$

CD8 T cells:

- $$\text{CD8+ T cells cells/}\mu\text{L} = \frac{(\text{CD45}^{+}\text{ mononuclear cells absolute count from counting tube}) \times (\text{CD8+ T cells events})}{(\text{CD45+ mononuclear cells events})}$$

B cells:

- $$\text{CD19+B cells cells/}\mu\text{L} = \frac{(\text{CD45}^{+}\text{ mononuclear cells absolute count from counting tube}) \times (\text{B cells events})}{(\text{CD45+ mononuclear cells events})}$$

CD14+ monocytes cells:

- $$\text{CD14+ monocytes cells/}\mu\text{L} = \frac{(\text{CD45}^{+}\text{ mononuclear cells absolute count from counting tube}) \times (\text{CD14+ monocytes events})}{(\text{CD45+ mononuclear cells events})}$$

NK cells:

- $$\text{NK cells cells/}\mu\text{L} = \frac{(\text{CD45}^{+}\text{ mononuclear cells absolute count from counting tube}) \times (\text{NK cells events})}{(\text{CD45+ mononuclear cells events})}$$

- Enter absolute cell counts into WG2 ILS Reporting Spreadsheet.

1.8. Determination of instrument performance characteristics and ERF values of CD markers

- Insert (copy and paste values) raw data, MdFI, rCV and rSD of the Rainbow 8-peak beads obtained for the 8 fluorescence channels in the 'Column G', 'Column H', and 'Column I', respectively, in the 'Instrument' sheet of the WG2 ILS Reporting Spreadsheet to the respective columns in Row 30-37 for each fluorescence channel (labeled as FITC,-A, APC,-A, etc. in Row 29) in the 'Raw Data' sheet (2nd sheet) of the WG2 Cytometer Sensitivity and ERF Calibration spreadsheet. Raw data for the two fluorescence channels, FITC and APC is shown as examples in Figure 2. [**Note**: i) Users should make a copy of the original WG2 Cytometer Sensitivity and ERF Calibration spreadsheet and insert your data in the copy of the spreadsheet; ii) Users are not allowed to make any changes in cells highlighted in blue; iii) Data from minimal 5 peaks with distinguishable fluorescence signals should be entered starting from Row 30. Peak_01 does not have to be the corresponding peak 1 in the Rainbow 8-peak beads.

28	**Sample**	**FITC-A**			**APC,-A**			**APC-Cy7,-A**
29	**Statistic**	FITC-A	CV,FITC-A	SD,FITC-A	APC,-A	CV,APC,-A	SD,APC,-A	APC-Cy7,-A
30	**Peak_01**	17.22	1.4825784	25.53	58.32	0.720679	42.03	4.86
31	**Peak_02**	291.92	0.12078652	35.26	613.17	0.2076096	127.3	44.55
32	**Peak_03**	826.56	0.06618999	54.71	1674.67	0.1337398	223.97	118.26
33	**Peak_04**	2415.72	0.04076217	98.47	4774.95	0.0767087	366.28	349.92
34	**Peak_05**	5971.65	0.02799226	167.16	11856.38	0.0497825	590.24	865.89
35	**Peak_06**	17266.74	0.02084122	359.86	34175.52	0.0371424	1269.36	2536.11
36	**Peak_07**	51448.44	0.01775797	913.62	97947.63	0.0306639	3003.46	7610.36
37	**Peak_08**	108846.39	0.01710576	1861.9	165218.95	0.0308551	5097.85	14037.3
38	**Peak_09**							
39	**Peak_10**							
40	**FS Bead ERF**	35072.16			42203.06			32284.44
41	**FS Bead Mean**	21311.00			35279.00			13758.00
42	**FS Bead rSD**	1590.00			2006.00			
43	**CDx Mean**				20000.00			
44	**CDx rSD**							
45	**CDy Mean**				25000.00			
46	**Cdy rSD**							
47								

Figure 2. A portion of the data sets in the 'Raw Data' sheet of the WG2 Cytometer Sensitivity and ERF Calibration spreadsheet.

- Enter MdFI and rSD of respective FC bead obtained from SOP-1 and recorded in the 'Instrument' sheet of the WG2 ILS Reporting Spreadsheet in Row 41 and 42 in the same 'Raw Data' sheet as shown in Figure 2.
- Insert an ERF value assigned by NIST to each FC bead in Row 40 in the 'Raw Data' sheet. The NIST assigned ERF values for the 8 FC beads will be provided to participants separately.
- Enter the MdFI and rSD values of the different cell markers in the respective Column and Rows in the 'Raw Data' sheet of the WG2 Cytometer Sensitivity Spreadsheet (Rows 43-46).

- Press **only once** the ‘Analyze Data’ button on the top of the ‘Raw Data’ sheet. The automatic calculations will be proceeded using the ‘Analysis Template’ sheet (1st sheet of the spreadsheet) and raw data entered in the “Raw Data’ sheet. The outputs of the automatic calculations are the individual fluorescence channel/detector worksheets added to the spreadsheet before the ‘Analysis Template’ sheet. Note: for more information on the use of the automated WG2 Cytometry Sensitivity and ERF Calibration spreadsheet, please see ‘Using the Excel Spreadsheet to Analyze Multi-Intensity Ranging Data’.
- The cytometer performance characteristics generated from the ‘Analysis Data’ program in Step above are reported in the ‘Summary’ sheet (last sheet) of the spreadsheet. An example summary report is provided in Figure 3.

Summary										
Date:	19-May-2022									
0										
0										
Sample Name	Spe (Q_{Spe})	B_{Spe}	B_{IU}	$CV_{Intrinsic}$	Q_{ERF}	B_{ERF}	CDx_{ERF}	CDy_{ERF}	Goodness of Fit	Model Used
Analysis Template	1.82E-01	9.65E+02	5.30E+03	1.37E-02	1.82E-01	5.30E+03			9.94E-01	QUAD FIT
FITC-A	4.36E-01	4.78E+02	1.10E+03	1.65E-02	2.65E-01	6.66E+02			9.58E-01	QUAD FIT
APC,-A	4.13E-01	1.74E+04	4.22E+04	3.05E-02	3.45E-01	3.53E+04	2.39E+04	2.99E+04	8.28E-01	QUAD FIT
APC-Cy7,-A	1.32E-01	-1.70E+01	-1.29E+02	1.62E-02	5.63E-02	-5.49E+01			1.84E-40	QUAD FIT
PE,-A	4.07E-01	2.08E+03	5.12E+03	2.31E-02	6.17E-02	7.77E+02			8.48E-01	QUAD FIT
PE-Cy7,-A	1.54E+01	3.68E+05	2.39E+04	1.96E-02	6.97E-01	1.08E+03			9.33E-01	QUAD FIT
Pacific Blue,-A	-3.66E-01	4.01E+03	-1.10E+04	2.10E-02	-3.66E-01	-1.10E+04			6.12E-01	QUAD FIT
Aqua,-A	-1.92E-02	7.60E+00	-3.96E+02	1.10E-01	-1.92E-02	-3.96E+02			4.33E-04	QUAD FIT
PerCP-Cy5.5,-A	1.82E-01	9.65E+02	5.30E+03	1.37E-02	1.82E-01	5.30E+03			9.94E-01	QUAD FIT

Figure 3. Example summary report on the instrument performance characteristics and ERF values of CD markers.

- Report Q_{ERF} values for 8 fluorescence channels/detectors in Column ‘J’ and the analysis date/operator in Column ‘K’, respectively in the ‘Instrument’ sheet of the WG2 ILS Reporting Spreadsheet.
- Report ERF values for 7 CD markers in Column ‘J’ and the analysis date/operator in Column ‘K’, respectively in the ‘Sample Data Analysis’ sheet of the WG2 ILS Reporting Spreadsheet. Optional: Participant can record sample filename (either Local Filename from the ‘Test and QC Samples’ sheet or FCSC ILS Filename from the ‘FCSC ILS Filenames’ sheet) used for the analysis in Column ‘L’ using copy/paste function keys. This Column ‘L’ serves as a sample file place holder for participant to track the fcs file used for value determination.
- Copy and paste the summary report table alike Figure 3 in the ‘Summary’ sheet as an image at the bottom of the ‘Sample Data Analysis’ sheet of the WG2 ILS Reporting Spreadsheet (below Row 314).

1.9. File Export for WG3

- Complete the WG2 ILS Reporting Spreadsheet and return to NIST.

Attachments

Appendix A: Screenshot of counting tube for Cryopreserved PBMCs
Appendix B: Screenshot of analysis template for full stain of Cryopreserved PBMCs
Appendix C: Screenshot of Trucount™ counting tube for QC Synthetic Cell Trucytes
Appendix D: Screenshot of analysis template for full stain of QC Synthetic Cell Trucytes
Slingshot TDS for Trucytes TBMNK

Appendix A: Analysis Template for Counting Tube:

Cryopreserved PBMCs

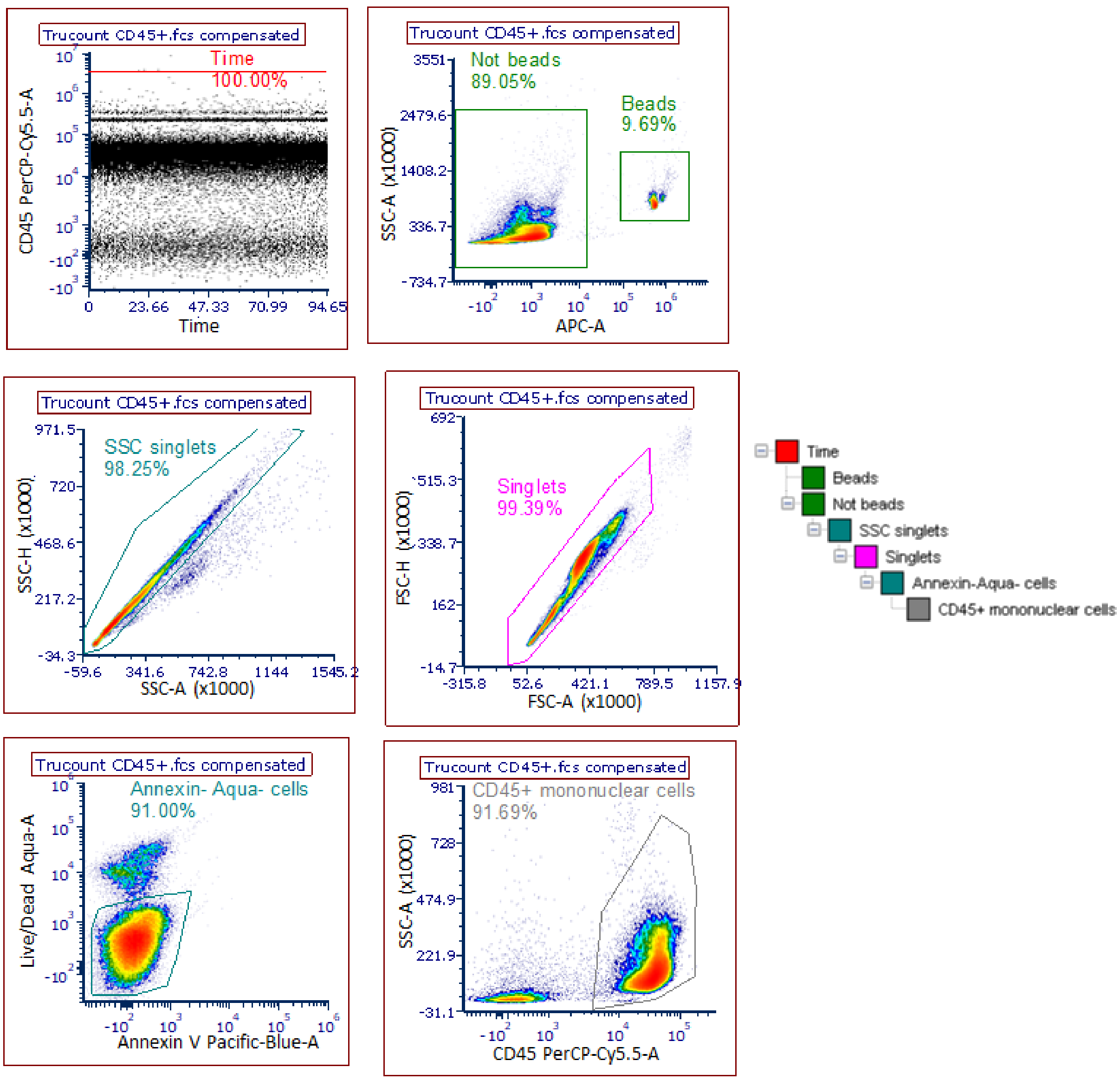

Appendix B: Analysis Template for TBMNK Assay: Cryopreserved PBMCs

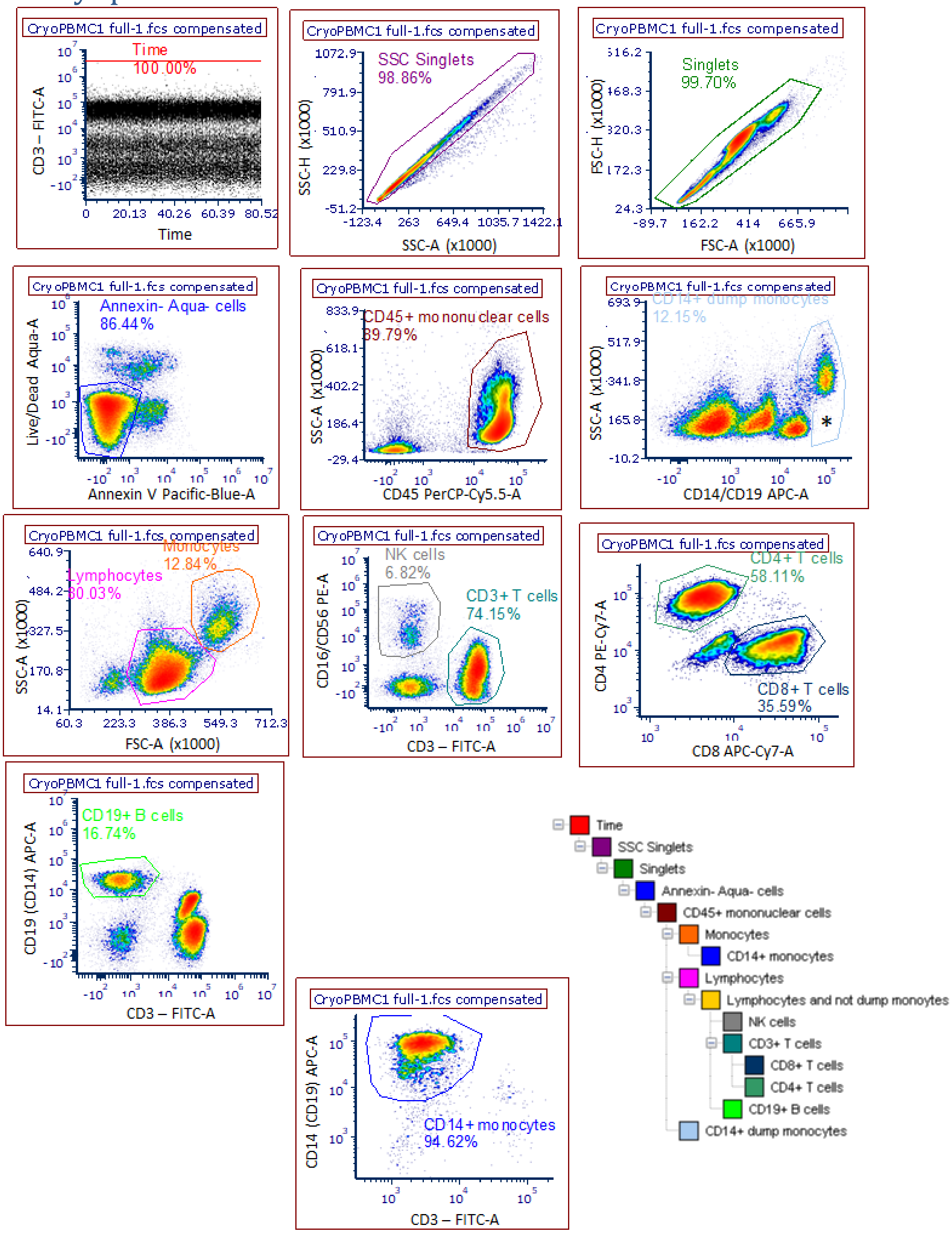

Appendix C: Analysis Template for Trucount™ Counting Tube: QC Synthetic Cell Trucytes

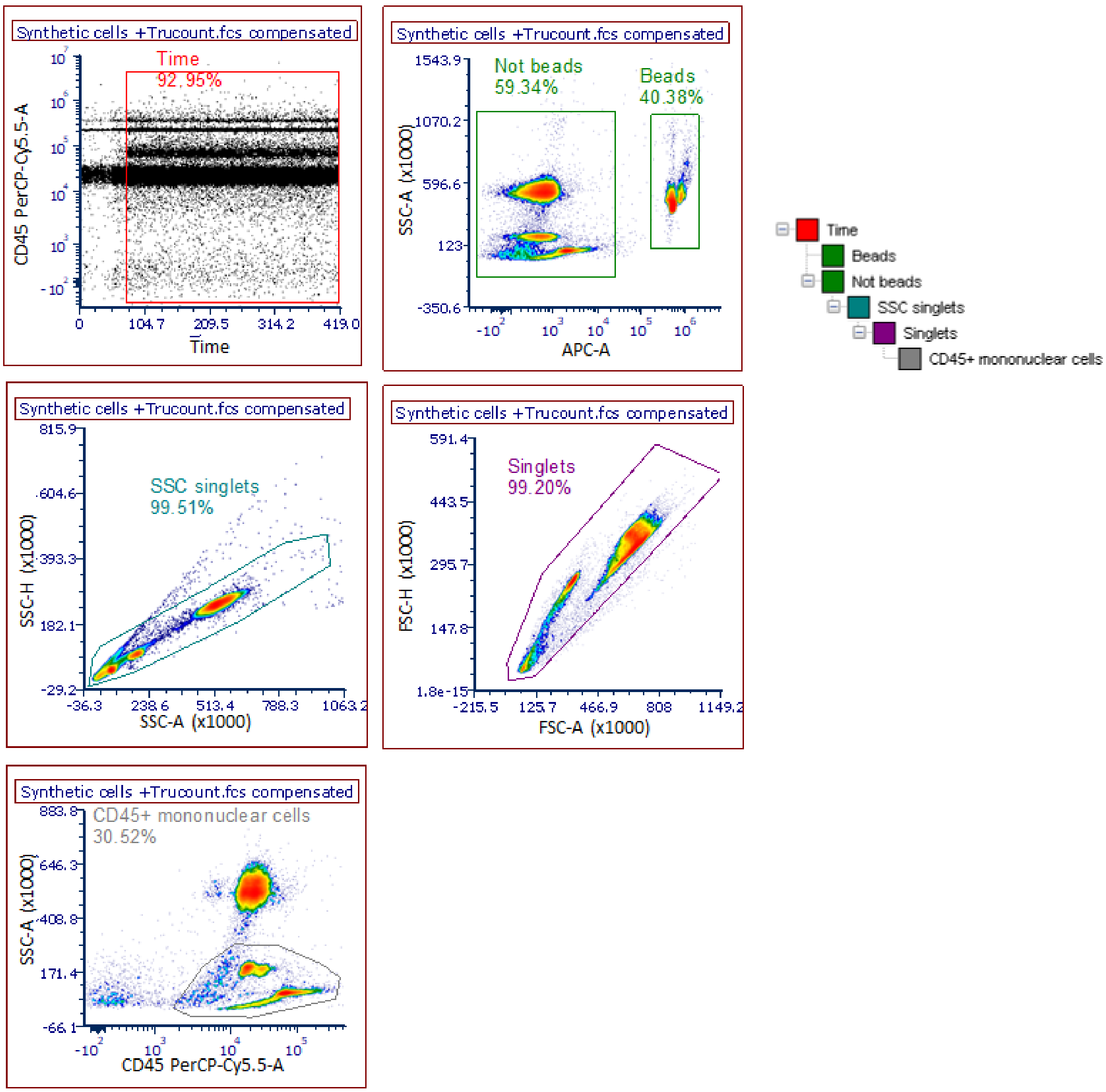

Appendix D Analysis Template for TBMNK Tube:

QC Synthetic Cell Trucytes

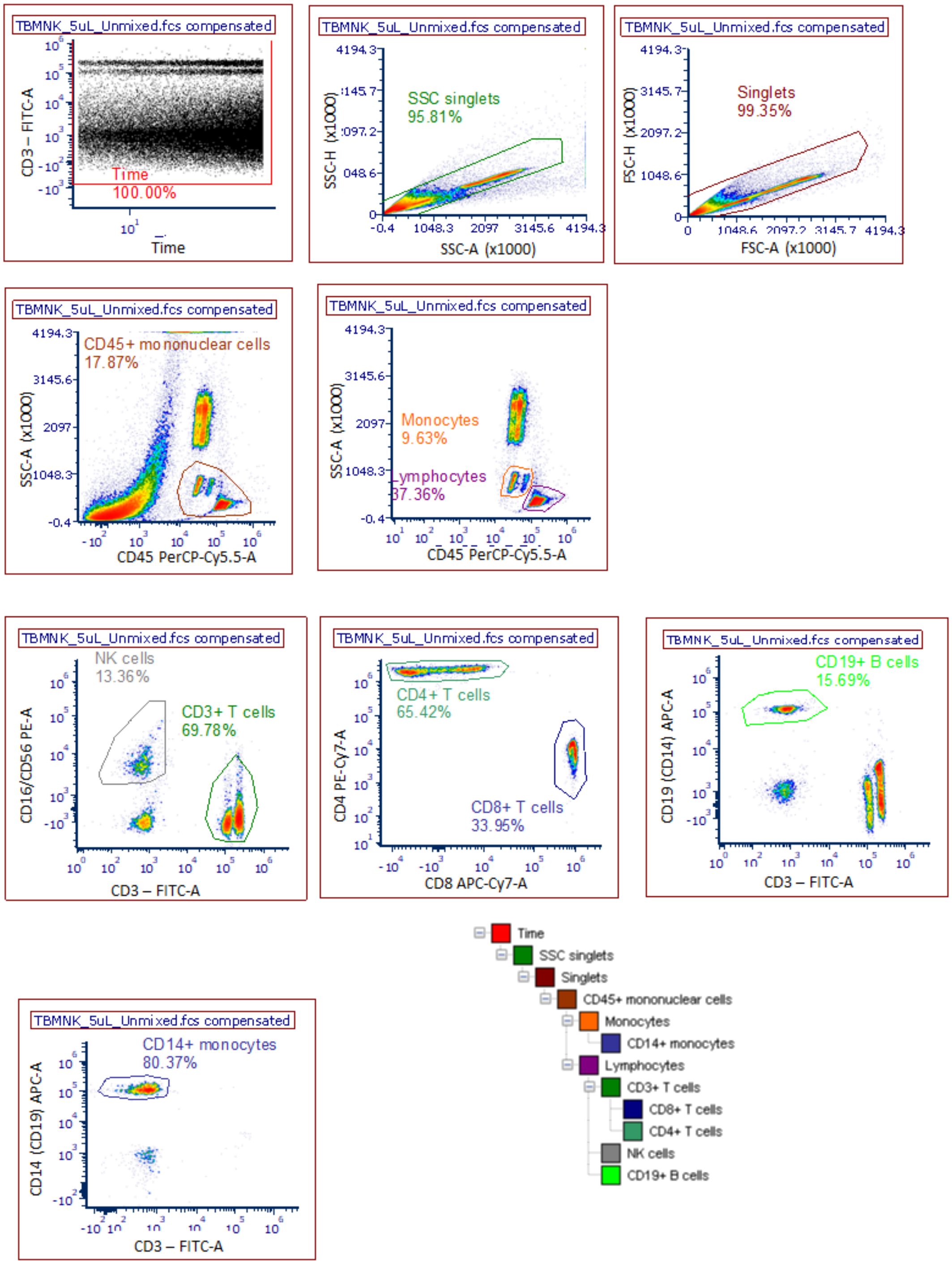

Supplementary Table 1: Number of different flow cytometer platforms used in the ILS and their manufacturers.

Flow Cytometer	N (Enrolled)	n (Passed QC)	n (Site PBMC Results)	n (Site TruCyte Results)	Detector Type	Manufacturer
NovoCyte Quanteon	1	1	1	1	SiPM	Agilent
NovoCyte Penteon	1	0	0	0	SiPM	Agilent
CytoFLEX	1	0	0	0	APD	Beckman Coulter Life Sciences
CytoFLEX S	3	1	1	1	APD	Beckman Coulter Life Sciences
CytoFLEX LX	3	1	1	1	APD	Beckman Coulter Life Sciences
FACSAria III	2	2	2	2	PMT	BD Biosciences
FACSCanto II	4	4	3	3	PMT	BD Biosciences
FACSCanto 10	1	1	1	1	PMT	BD Biosciences
FACSCanto SORP	3	2	2	2	PMT	BD Biosciences
FACSLyric	3	2	2	2	PMT	BD Biosciences
Fortessa	2	2	1	1	PMT	BD Biosciences
Fusion	2	2	0	0	PMT	BD Biosciences
Symphony A3	1	1	1	1	PMT	BD Biosciences
Symphony A5	1	1	1	1	PMT	BD Biosciences
ZE5	1	1	1	1	PMT	Bio-Rad
Aurora	5	5	5	3	APD	CyTek Biosciences
Northern Lights	1	0	0	0	APD	CyTek Biosciences
Amnis CellStream	3	2	2	2	CCD	CyTek Biosciences
Amnis ImageStreamX	1	1	1	1	CCD	CyTek Biosciences
MACSQuant Analyzer 10	2	2	2	2	PMT	Miltenyi Biotec
Attune NxT	1	1	1	1	PMT	Thermo Fisher Scientific

N (Instruments Enrolled): Total number of flow cytometers of this model enrolled in the ILS. n (Passed QC): Number of instruments from which FCS files passed all QC criteria. n (Site PBMC Results): Number of instruments from which site-reported manual data analysis results for the PBMC assay were submitted. n (Site TruCyte Results): Number of instruments from which site-reported manual data analysis results for TruCyte were submitted. Detector types — SiPM: silicon photomultiplier; APD: avalanche photodiode; PMT: photomultiplier tube; CCD: charge-coupled device.

Supplemental Table 2: ERF values assigned to the FC beads using reference fluorophores and under the standard filters commonly used in flow cytometer platforms and defined by NIST.

FC Beads	Standard Filter Ex. / Em. / Bandwidth	Reference Fluorophore	ERF
V450	405-450-50	Coumarin 30	4.62E+05
V500-C	405-525-50	Coumarin 30	2.15E+05
FITC	488-525-50	Fluorescein	2.10E+05
PE	488-585-42	Nile Red	4.44E+06
	561-585-42	Nile Red	3.20E+06
PerCP-Cy5.5	488-690-50	Nile Red	3.59E+05
PE-Cy7	488-780-60	Nile Red	2.74E+05
	561-780-60	Nile Red	6.49E+05
APC	633-670-30	APC	1.12E+05
APC-Cy7	633-780-60	Alexa Fluor 700 (AF700)	5.88E+05

Abbreviation: ERF: Equivalent number of Reference Fluorophore; NIST, National Institute of Standards and Technology.

Supplemental Table 3: ARI values assigned by NIST to the FC beads using reference fluorophores and under the filters used in the 8 FACSCanto flow cytometers by the study participants.

FC Beads	Reference	Canto1		Canto2		Canto3		Canto4		Canto5		Canto6		Canto7		Canto8	
	Fluorophore	EM Filter	ARI	EM Filter	ARI	EM Filter	ARI	EM Filter	ARI	EM Filter	ARI	EM Filter	ARI	EM Filter	ARI	EM Filter	ARI
V450	Coumarin 30	405_450_50	4.62E+05	405_450_50	4.62E+05	405_450_50	4.62E+05	405_450_50	4.62E+05	405_450_50	4.62E+05	405_450_50	4.62E+05	405_450_50	4.62E+05	405_450_50	4.62E+05
V500-C	Coumarin 30	405_525_50	2.15E+05	405_525_50	2.15E+05	405_525_50	2.15E+05	405_525_50	2.15E+05	405_510_50	2.08E+05	405_510_50	2.08E+05	405_510_50	2.08E+05	405_510_50	2.08E+05
FITC	Fluorescein	488_530_30	1.27E+05	488_530_30	1.27E+05	488_530_30	1.27E+05	488_530_30	1.27E+05	488_530_30	1.27E+05	488_530_30	1.27E+05	488_530_30	1.27E+05	488_530_30	1.27E+05
PE	Nile Red	488_586_15	1.54E+06	488_586_15	1.54E+06	488_586_15	1.54E+06	488_575_25	3.64E+06	488_585_42	4.44E+06	488_585_42	4.44E+06	488_585_42	4.44E+06	488_585_42	4.44E+06
PerCP-Cy5.5	Nile Red	488_710_50	2.51E+05	488_710_50	2.51E+05	488_710_50	2.51E+05	488_695_40	2.86E+05	488_715_90	4.43E+05	488_715_90	4.43E+05	488_715_90	4.43E+05	488_715_90	4.43E+05
PE-Cy7	Nile Red	488_780_60	2.74E+05	488_780_60	2.74E+05	488_780_60	2.74E+05	488_780_60	2.74E+05	488_780_60	2.74E+05	488_780_60	2.74E+05	488_780_60	2.74E+05	488_780_60	2.74E+05
APC	APC	640_670_30	1.12E+05	640_670_30	1.12E+05	640_670_30	1.12E+05	640_670_30	1.12E+05	633_660_20	9.26E+04	633_660_20	9.26E+04	633_660_20	9.26E+04	633_660_20	9.26E+04
APC-Cy7	Alexa Fluor 700	640_780_60	5.88E+05	640_780_60	5.88E+05	640_780_60	5.88E+05	640_780_60	5.88E+05	633_780_60	5.88E+05	633_780_60	5.88E+05	633_780_60	5.88E+05	633_780_60	5.88E+05

Supplemental Table 4: ARI values assigned by NIST to the FC beads using reference fluorophores and under the filters used in the 5 Aurora flow cytometers by the study participants.

FC Beads	Reference	Aurora1		Aurora2		Aurora3		Aurora4		Aurora5	
	Fluorophore	EM Filter	ARI	EM Filter	ARI	EM Filter	ARI	EM Filter	ARI	EM Filter	ARI
V450	Coumarin 30	405_458_15	1.72E+05	405_458_15	1.72E+05	405_458_15	1.72E+05	405_458_15	1.72E+05	405_458_15	1.72E+05
V500-C	Coumarin 30	405_525_17	7.69E+04	405_542_17	6.92E+04	405_542_17	6.92E+04	405_542_17	6.92E+04	405_542_17	6.92E+04
FITC	Fluorescein	488_525_17	8.58E+04	488_525_17	8.58E+04	488_525_17	8.58E+04	488_525_17	8.58E+04	488_525_17	8.58E+04
PE	Nile Red	561_577_20	2.19E+06	488_581_19	2.69E+06	561_577_20	2.19E+06	561_577_20	2.19E+06	561_577_20	2.19E+06
PerCP-Cy5.5	Nile Red	488_697_19	1.32E+05	488_697_19	1.32E+05	488_697_19	1.32E+05	488_697_19	1.32E+05	488_697_19	1.32E+05
PE-Cy7	Nile Red	561_780_30	4.71E+05	488_783_23	1.58E+05	561_780_30	4.71E+05	561_780_30	4.71E+05	561_780_30	4.71E+05
APC	APC	640_679_18	4.46E+04	640_661_17	8.24E+04	640_661_17	8.24E+04	640_661_17	8.24E+04	640_661_17	8.24E+04
APC-Cy7	Alexa Fluor 700	640_783_23	2.96E+05	640_783_23	2.96E+05	640_783_23	2.96E+05	640_783_23	2.96E+05	640_783_23	2.96E+05

Supplemental Table 5: Different flow cytometer platforms from which the fcs files have passed the QC step are equipped with red lasers with different wavelengths and different lasers for detecting PE and PE-Cy7.

Flow cytometer	Number	Red Laser Excitation Wavelength	Laser Excitation Wavelength for PE and PE-Cy7	Manufacturer
NovoCyte Quanteon	1	640 nm	561 nm	Agilent
CytoFLEX S	1	638 nm	561 nm	Beckman Coulter Life Sciences
CytoFLEX LX	1	638 nm	561 nm	Beckman Coulter Life Sciences
FACSAria III	2	633 nm	561 nm	BD Biosciences
FACSCanto II	4	633 nm	488 nm	BD Biosciences
FACSCanto 10	1	640 nm	488 nm	BD Biosciences
FACSCanto SORP	2	640 nm	488 nm	BD Biosciences
FACSLyric	2	640 nm	488 nm	BD Biosciences
Fortessa	1	628 nm	561 nm	BD Biosciences
Fortessa	1	640 nm	561 nm	BD Biosciences
Fusion	2	640 nm	561 nm	BD Biosciences
Symphony A3	1	640 nm	532 nm	BD Biosciences
Symphony A5	1	640 nm	532 nm	BD Biosciences
ZE5	1	640 nm	561 nm	Bio-Rad
Aurora	5	640 nm	488 nm (1 Instr.) & 561 nm (4 Instr.)	CyTek Biosciences
Amnis CellStream	2	642 nm	561 nm	CyTek Biosciences
Amnis ImageStreamX	1	642 nm	488 nm	CyTek Biosciences
MACSQuant Analyzer 10	2	640 nm	488 nm	Miltenyi Biotec
Attune NxT	1	638 nm	561 nm	Thermo Fisher Scientific

A.

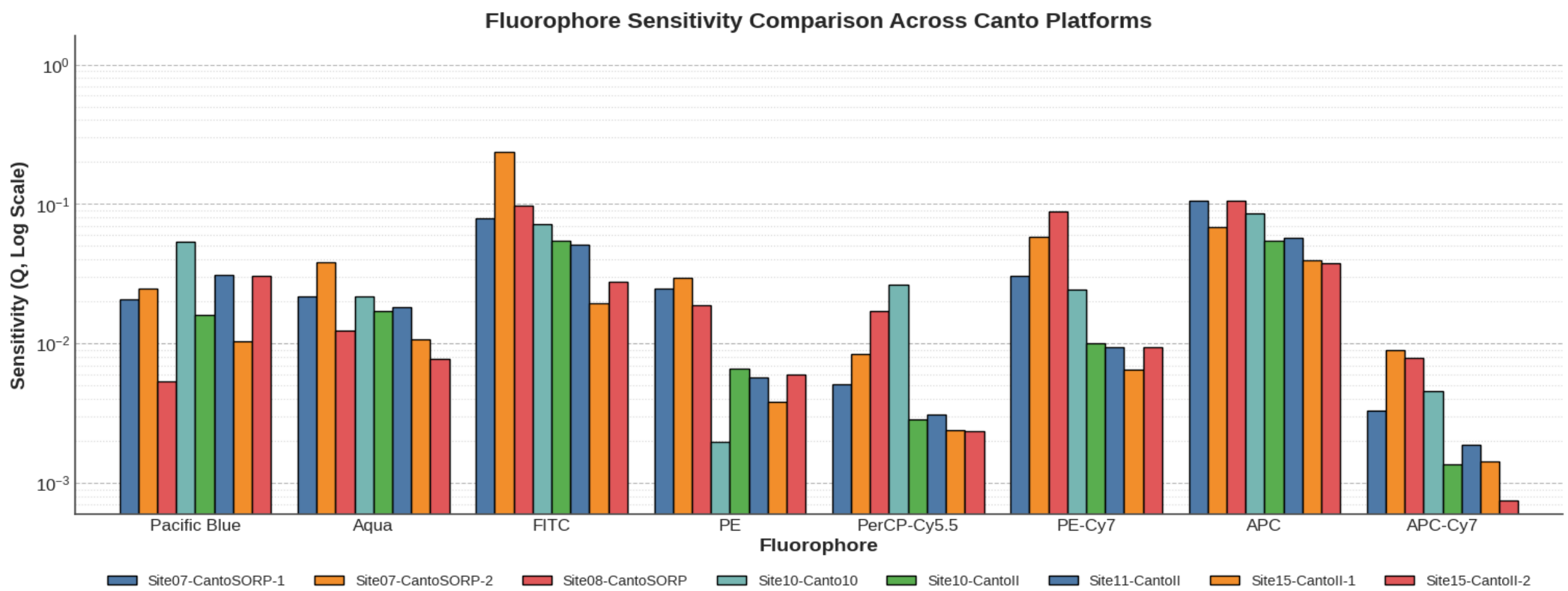

B.

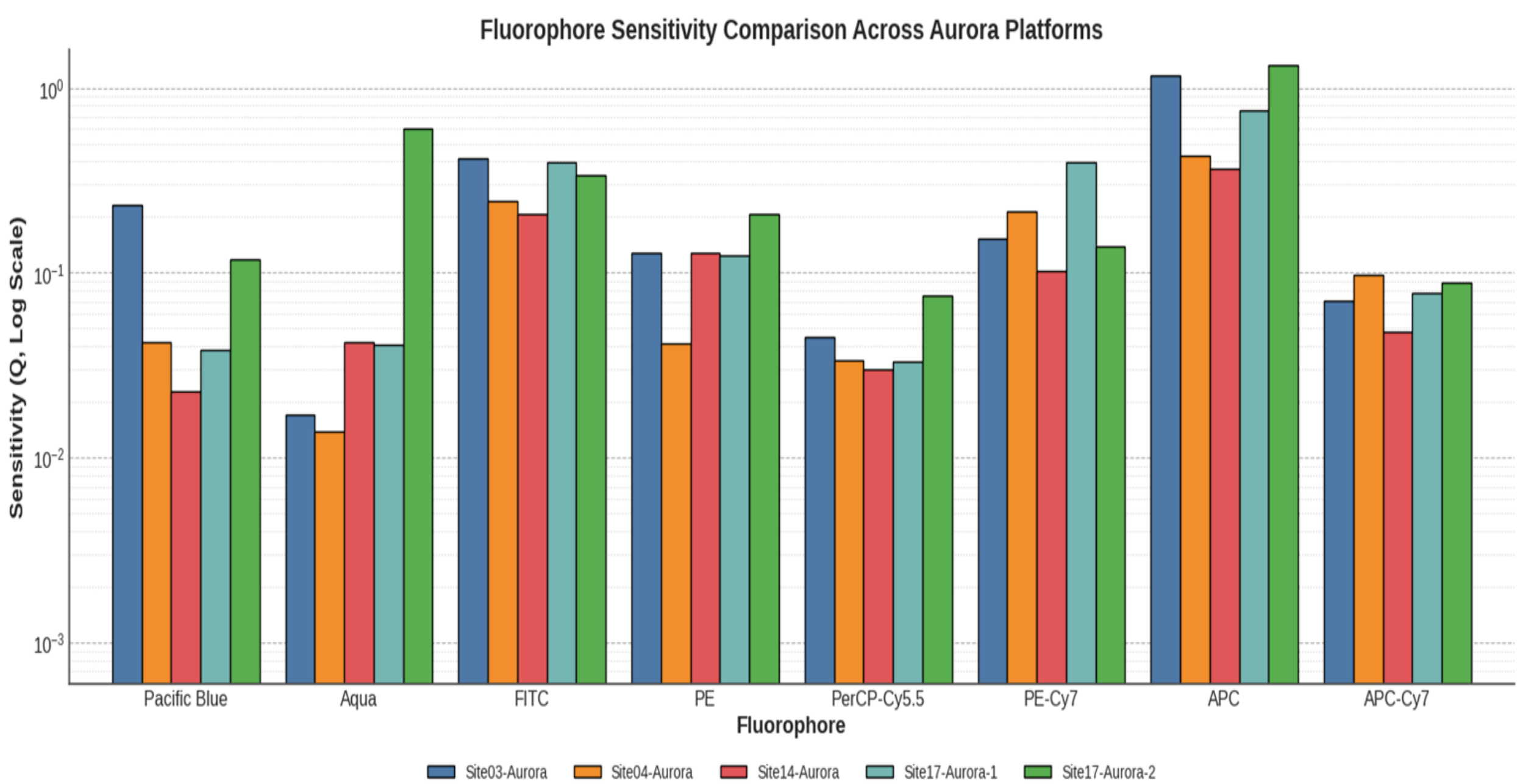

Supplemental Figure 1 - Comparison of Cytometer Detector Sensitivity (Q Value) in Standardized Spe/ARI Units for FACSCanto and Aurora Platforms

Detector sensitivity (Q value) expressed in standardized Spe/ARI units for each fluorophore channel, compared across individual instrument units within CantoII and Auroa platforms. The y-axis is displayed on a $\log_{10}$ scale. Differences in bar height across site-instruments within the same platform reflect inter-instrument variability in detector sensitivity, which motivates the calibration step in the centralized data analysis pipeline.
